# Effects of rootstocks and developmental time on the dynamic changes of main functional substances in ‘Orah’ (*Citrus reticulata* Blanco) by HPLC coupled with UV detection

**DOI:** 10.3389/fpls.2024.1382768

**Published:** 2024-08-27

**Authors:** Shuang Li, Lei Yang, Min Wang, Yang Chen, Jianjun Yu, Hao Chen, Haijian Yang, Wu Wang, Zhiyong Cai, Lin Hong

**Affiliations:** Research Institute of Pomology, Chongqing Academy of Agricultural Sciences, Chongqing, China

**Keywords:** mandarin, phenolic compounds, bioactive compounds, antioxidant activity, fruit quality

## Abstract

**Introduction:**

Citrus fruit is rich in important functional constituents such as flavonoids, phenolic acids terpenes and other functional substances that play an important role for treating clinical diseases or controlling major agricultural diseases and pests. Plant secondary metabolites have become one of the most important resources of novel lead compounds, especially young citrus fruits contain multiple functional substances. ‘Orah’, a type of citrus reticulata, is known for its fine appearance, productivity, delicious sweetness, late-maturing characteristics, and is widely cultivated in China. Fruit thinning and rootstock selection are commonly used agronomic measures in its production to ensure its quality and tree vigor. However, few studies have demonstrated the effects of these agronomic measures on the functional substances of ‘Orah’.

**Methods:**

In this study, we used HPLC coupled with UV to detect the dynamic changes of fruit quality, 13 main flavonoids, 7 phenolic acids, 2 terpenes, synephrine and antioxidant capacity in both peel and pulp of citrus fruits grafted on four rootstocks (Red orange *Citrus reticulata* Blanco cv. red tangerine, Ziyang xiangcheng *Citrus junos* Sieb. ex Tanaka, Trifoliate orange *Poncirus trifoliata* L. Raf, and Carrizo citrange *Citrus sinensis* Osb.×*P.trifoliate* Raf) at six different developmental stages (from 90 DAF to 240 DAF).

**Results:**

The results indicated that rootstock can significantly affect the contents of functional constituents and antioxidant capacity in ‘Orah’. Additionally, it was found that pruning at either 90 DAF (days after flowering) or 150 DAF produced the most favorable outcomes for extracting functional substances. We also identified rootstock ‘Trifoliate orange’ has the highest total soluble solids (TSS) and ‘Ziyang xiangcheng’ to be the optimal in terms of comprehensive sensory of fruit quality, while ‘Red orange’ and ‘Ziyang xiangcheng’ are optimal in terms of functional substance quality, and ‘Red orange’ excels in antioxidant capacity.

**Discussion:**

Overall, the findings demonstrate the important role of rootstocks and developmental stage in shaping fruit sensory quality and functional substance synthesis, providing valuable insights for guiding rootstock selection, determining thinning time, and utilizing pruned fruits in a more informed manner.

## Graphical Abstract

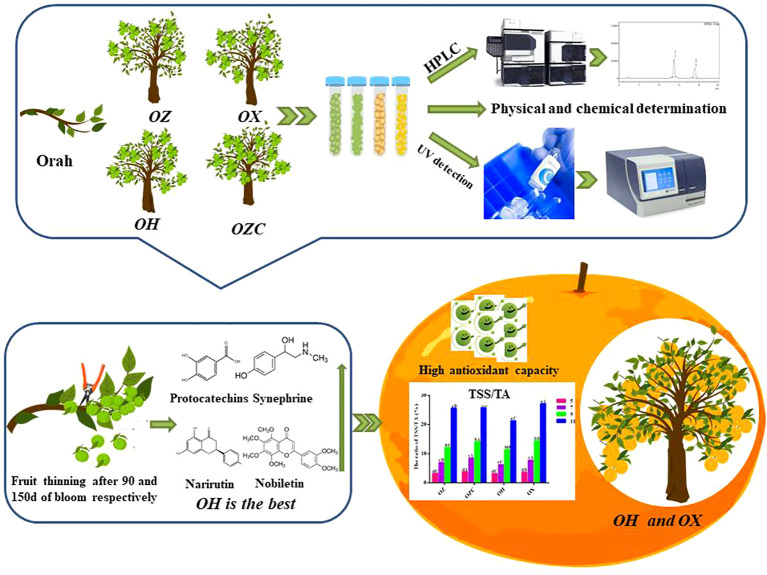

## Introduction

Most natural products of medicament come from plants, animals, and microorganisms. According to records, the history of humans using plants as pharmaceuticals could be traced back to at least 60000 years ago (Shi et al., 2010). After millions of years of evolution, natural products have unique chemical diversity, leading to a diversity of their biological activity and medical properties, with great potential for treating human diseases, especially critical illnesses (Simmonds, 2003; Lattanzio et al., 2006; Galm and Shen, 2007). As early as the 1930s, many scholars discovered that citrus serves as a natural source of multiple antioxidants and bioactive substances (Robbins, 1980), rich not only in primary metabolites such as organic acids, sugars and amino acids (Shi et al., 2010; Benjamin et al., 2013), but also in valuable secondary metabolites such as carotene, polyphenols (particularly flavanones and flavonoids, phenolic acids, normilin and limonin) (Tanaka et al., 2000; Goldenberg et al., 2018), which can treat and alleviate human cardiovascular, cerebrovascular, tumor, blood and other diseases. Among them, citrus flavonoids also can be used to control various pests such as grashoppers, *Spodoptera litura*, etc (Aboshi et al., 2018; Cui et al., 2019). Naringin, naringenin, and hesperidin have insecticidal activity (Franceschini Sarria et al., 2022). The pharmacological mechanism of naringin to play an anti-cancer role in blocking tumor cell cycle, inhibiting tumor cell proliferation, alleviate Adverse drug reaction of chemotherapy, activate and strengthen immunity (He and Zhang, 2023). Citrus fruits also contain a numerous phenolic acids such as ferulic acid, p-coumaric acid, sinapic acid, and caffeic acid, vanillic acid, etc., all of which serve as antioxidants that coordinate and stimulate metabolic transformations to protect tissues and body fluids from damages associated with the presence of reactive oxygen species (Silva et al., 2000; Czech et al., 2021). Therefore, planting citrus crops with high phenolic compounds can not only control human diseases, but also prevent and control pests, providing materials for the preparation of pesticides and medicine.

The chemical composition and biological activity of citrus fruits are influenced by various factors, including cultivation methods (water and fertilizer management, grafting), growing environment, and fruit ripeness (Li et al., 2022). Traditionally, using rootstocks to improve varieties is a normal practice in citrus cultivation, including good compatibility and adaptability, vigorous and healthy rootstocks are essential to improving fruit yield, functional substance content, overall quality, resistance, as well as the nutritional status of grafted plants. These factors ultimately affect the economic outcomes of orchards (Albrecht et al., 2018; Morales et al., 2023). Different rootstocks, citrus varieties, and even different combinations of rootstock strains exhibit significant differences, ultimately affecting their chemical compositions (Benjamin et al., 2013). For example, the scion variety Fremont mandarin, exhibited superior plant growth and fruit yield on common rootstocks (rough lemon or Karna khatta). Similarly, Pectinifera rootstock not only increases yield but also exhibited environmental stress (Albrecht et al., 2018). ‘Rough lemon’-1 (scion *C. Nobilis*× *C. deliciosa*) provided the necessary concentrations of sugars, acids, phenols, vitamins, and low limonin. Furthermore, rootstocks like ‘Lime’ and ‘Shekwasha’ induced high production of limonin in ‘Kinnow’, while the rootstocks such as ‘Shekwasha’, ‘Sour Orange’, and ‘Pectinifera’ led to higher concentrations of flavonoids, quercetin, and hesperidin in ‘Kinnow’ fruits (Albrecht et al., 2018; Saini et al., 2019). However, the distribution and content of these compounds are not uniform in the peel and pulp of fruit at different stages of development. A study has found that the concentration of individual functional substances is higher in the pulp than in the peel (Ordóñez-Díaz et al., 2020). While other scholars believe that antioxidants in citrus peel are higher than those in other fruits and the pulp of the citrus fruit (Singh et al., 2020). It is puzzling that the distribution of functional substances in the peel and pulp of citrus fruit is not clear at different stages of fruit development.

Introduced to China in 2004, ‘Orah’ is a variety bred by crossing ‘Temple’ tangor and ‘Dancy’ mandarin in Israel. ‘Orah’ possess the characteristics such as strong tree vigor, early fruiting and maximum yield, late ripening and storage capabilities, transportation resistance, crisp and sweet pulp, a rich and juicy flavor. These qualities significantly increased the income of fruit farmers in the region (Jiang and Cao, 2011). The rootstocks commonly used for grafting ‘Orah’ in China include Red orange (*Citrus reticulata* Blanco cv. Red tangerine) representing *‘H’ *(Liu et al., 2017), Ziyang xiangcheng (Citrus junos Sieb. ex Tanaka) representing ‘*X* ‘ (He et al., 2022), Trifoliate orange (*Poncirus trifoliata* (L.) Raf)) representing ‘*Z* ‘ (Sheng et al., 2009) and Carrizo citrange (*Citrus.sinensis* Osb.×*P.trifoliate* Raf) representing ‘*ZC’* (Morales et al., 2021), most of which have achieved significant success in the field of applied research. For example, ‘*Z’* is one of the most common traditional Chinese medicines, and various new anti-tumor drugs have been explored through the development and utilization of its active ingredients (Zhang et al., 2015). In 2012, ‘Orah’ had been widely planted and developed into the main variety of late-maturing mandarin oranges in China (Wu et al., 2021). In our preliminary research, UPLC-MS/MS (ultra-performance liquid chromatography-tandem mass spectrometry) was performed to analyze the metabolites of Orah’ grafted on four rootstocks (‘Trifoliate orange’, ‘Carrizo citrange’, ‘Red tangerine’ and ‘Ziyang Xiangcheng’), and a considerable difference between the different rootstocks was also observed in the accumulation of lipids, phenolic acids and flavonoids (Wang et al., 2024). Unfortunately, the lack of knowledge on the functional components of ‘Orah’s pulp and peel grafted on different rootstocks at each developmental stages obstructs the utilization of functional substances in young fruits. Furthermore, the inner qualities resulting from grafting onto different rootstocks, especially the chemical composition of ‘Orah’ such as its phenolic acids, flavones, limonin, and antioxidant capacity, have not been studied thoroughly. Previous studies have shown that multiple citrus varieties, especially citrus hybrids, require thinning during the young fruit stage to improve the quality of mature fruits and tree vigor (Ouma, 2012). As a citrus hybrid, the optimal thinning time of ‘Orah’ was still unclear.

Although research on rootstock-scion interactions has seen gradual progress in recent years (Tietel et al., 2020), our understanding of the extent on the functional substances content of ‘Orah’ at different developmental stages remains limited. Vitamins, polyphenols, and limonin, etc. are key metabolites that affect the quality of citrus (Gong et al., 2015). The selection of suitable rootstocks can promote fruit quality and functional components. So far, no study has been conducted on the influence of the rootstocks of ‘*H’*, ‘*X’*, ‘*Z’*, and ‘*ZC’* on the content of functional substances and antioxidant activity in the fruit and peel of citrus. On the other hand, the amount of fruit planted in ‘Orah’ is huge, thinning is necessary to ensure the normal growth of the tree and improve the fruit quality, but the optimal time of fruit-thinning has not been determined yet. Therefore, this study employs HPLC coupled with UV to evaluate the effects of rootstock on flavor, functional substances and antioxidant activity of ‘Orah’ fruits at different developmental stages. It aims to analyze the changes in functional and nutritional components of rootstock-scion combinations at different developmental stages, determine the suitable rootstock and fruit thinning time, and propose optimized utilization strategies for pruning fruits, providing direction for the acquisition of natural medicinal materials. At the same time, it also provides guidance for improving the the yield of ‘Orah’ by increasing income and reducing expenditure.

## Materials and methods

### Fruit sample and study site

From July 2019 to February 2020, fresh fruit samples of ‘Orah’ (*C.reticulata Blanco*) grafted onto ‘*H’*, ‘*X’*, ‘*Z’*, ‘*ZC’* rootstocks were obtained from a 5-year old experimental orchard located in the Jiangjin district of Chongqing. This area is one of the most suitable regions for citrus cultivation in Southwest China (N: 29°13′36.65″, E: 106°18′37.50″). Each rootstock was grafted with ‘Orah’ trees, there were 9 sample trees of each rootstock-scion. Fruits from each of the three trees was collected for a biological treatment, and this was repeated thrice. The experimental orchard’s soil type was a sandy and red soil, and a drip-irrigation system was used. According to the Chinese climate classification system, the local climate in the Xianfeng district is subtropical monsoon, with an annual rainfall of 1030.7 mm, primarily occurring from May to November. The temperature range varies widely throughout the year, with average temperatures of 7.7°C in winter and 28.5°C during spring and summer (https://czqxj.net.cn/qihou_814343).

Sampling of plants commenced on July 8, 2020, following the described and published protocol (Huang et al., 2009). Samples were collected at 90, 120, 150, 180, 210 and 240 days after full flower. Five samples were gathered from five different directions (East, South, West, North, and the middle of the crown periphery) in each plant. A total of 15 medium sized fruits without pests and diseases were collected from 3 sample trees as a biological replicate, and repeated 3 times. The collected samples were stored in an icebox and promptly transported to the laboratory. The peels (P) and pulps (R) were then separated using a sterilized blade, chopped and mixed, and then placed in a -80 °C ultra-low temperature refrigerator.

### Standards and reagents

The glassware and consumables used in the experiments included Eppendorf 100ml single channel pipette gun, Eppendorf 50-200 μL single channel pipette gun, dry nitrogen blowing instrument (Wuxi Woxin Instrument, China), a Shimadzu LC-20AT high performance liquid chromatograph, a Wufeng LC-100 high performance liquid chromatograph, a C18 column (250 mm×4.6 mm, 5 μm), and a cryogenic centrifuge TLG-16 (Hunan Xiangyi, China). The chemical standards and reagents used in the experiment included methanol (chromatographic grade, Shanghai Ampei), acetonitrile (chromatographic grade, Shanghai Amppo), acetic acid (chromatographic grade, Aladdin), sodium lauryl sulfonate (chromatographic grade, Shanghai Yuanye Co., Ltd.), phosphoric acid (chromatographic grade, Aladdin), formic acid (chromatographic grade, Aladdin), and potassium dihydrogen phosphate (analytical pure, Shanghai Yuanye Co., Ltd). Nomelin, limonin, synephrine, protocatechuic acid, p-hydroxbenzoic, vanillic acid, caffeic acid, sinapic acid, p-coumaric, ferulic acid, sinensetin, nobiletin, hesperidin, narirutin, tangeretin, eriocitrin, naringin, rhoifolin, vanillin, naringenin, hesperetin, neohesperidin and poncirin obtained from Sigma Corporation, USA were also used. The kit including amino acids, total phenols, total flavonoid, and free radical clearance ability (FRAP, ABTS, and DPPH), was procured from Suzhou Grace Biotechnology Co., Ltd. China.

### Physical and chemical determinations

Thirty ‘Orah’ were selected to determine their functional composition. Ten fruits served as one replicate, with the pulp and peel separated using a disinfected blade. Their weights were measured on an electronic balance (OHAUS AX223ZH/E) with 1mg accuracy. BRAun 4161 juicer was used to extract the juice for later use. The total soluble solids (TSS) and titratable acidity (TA) of ‘Orah’ mandarin fruit were determined using a digital refractometer (PAL-1; Atago, Tokyo, Japan) and volumetric neutralization, respectively, following the methods described by He and Zhang (2023). The TA and vitamin C contents were determined following GB8210-87 Chinese National Standard. Similarly, phenolic compounds, flavonoids, limonin, nomirin and synephrine were quantified using HPLC (Shimadzu LC-20AT and Wufeng LC-100).

#### Limonin and nomeline

Fresh samples of peel and pulp were separated using a sterilized blade. Portions of the samples were weighed (0.5 g), soaked in 1mL of acetonitrile, and subjected to 30 minutes of the ultrasonic treatment at 60°C using a 300 W ultrasonic equipment. The resultant supernatants were obtained, followed by centrifugation at 12,000 rpm for 10 minutes. This process was repeated 3 times and the supernatants were combined. The samples were then dried using nitrogen, after which 1mL of acetonitrile was added for proper dissolution. The obtained supernatant was filtered over a 0.22 μm organic filter membrane. The analysis was performed using HPLC (Shimadzu LC-20AT) on a C18 reverse phase chromatographic column (250 mm×4.6 mm, 5 μm). The running conditions consisted of phase A (methanol), and phase B (0.1% acetic acid water), at a ratio of A:B =35:65. The injection volume was 10 μL, with a flow rate of 1 mL min^-1^. The column temperature was maintained at 35°C, and the running time were 30 min. The UV detection was conducted at 210 nm.

#### Synephrine

Fresh samples of peel and pulp (0.5g each) were taken and soaked in 1 mL of 80% methanol water solution. The samples were then subjected to sonication at 300 W for 40 minutes, followed by centrifugation at 12,000 rpm for 10 minutes. The obtained supernatant was filtered with a 0.22 μm organic filter membrane. Analysis was performed using HPLC (Wufeng LC-100) with a C18 reverse-phase chromatography column (250 mm×4.6 mm, 5 μm). The running conditions consisted mobile phase A (methanol) and mobile phase B (0.1% potassium dihydrogen phosphate aqueous solution, containing 1% sodium dodecyl sulfonate and 2 mL acetic acid, at a ratio of A:B:=50:50. The column temperature was kept at 25°C. The running time was 30 minutes, and the wavelength of the UV detector wavelength was set at 275 nm.

#### Protocatechuic acid, p-hydroxbenzoic and vanillic acid

Fresh samples (0.5g) of peel and pulp were taken and soaked in 1mL of 60% methanol. The samples were extracted by ultrasonication at 60 °C for 30 minutes, heated at 80°C for 1.5 hours, then diluted to 1ml with 60% methanol water. Finally, they were centrifuged at 4°C, 12,000 rpm for 10 minutes. The resultant supernatant was filtered through a 0.22 μm filter membrane. The analysis was performed using HPLC (Wufeng LC-100) with a C18 reverse phase chromatography column (250 mm×4.6 mm, 5 μm). The running conditions included mobile phase A: (methanol) and mobile phase B: (0.1% phosphoric acid water), at a ratio of A:B=20:80. The injection volume was 10 μL and the flow rate was 1 mL min^-1^. The column temperature was kept at 25°C. The running time was 50 minutes, and UV detection maintained at 320 nm.

#### Caffeic acid, sinapic, acid ferulic acid and p-coumaric acid

Fresh samples (0.5 g) of peel and pulp were taken and soaked in 1 mL of 80% methanol. The samples were extracted by ultrasonication at 300 W for 40minutes. Samples were then centrifuged at 4°C, 12000 rpm for 10 minutes, to obtain supernatants. The resultant supernatants were filtered through a 0.22 μm filter membrane and analyzed using HPLC (Wufeng LC-100) following the same running conditions as explained for protocatechuic acid.

#### Sinensetin, nobiletin and tangeretin

Obtained fresh samples (0.5 g) of peel and pulp were mixed with 1 mL methanol DMSO (V/V=50:50) and placed at room temperature for 10 minutes. The samples were then centrifuged at 4°C, 9000 rpm for 15 minutes. This process was repeated 3 times and the resulting supernatants were combined. Subsequently, the samples were reduced under nitrogen to 0.5mL, after which 1mL of methanol was added. They were then filtered through a 0.22 μm filter membrane and analyzed using HPLC (Wufeng LC-100) on a C18 reverse phase chromatography column (250 mm×4.6 mm, 5 μm). The running conditions consisted of mobile phase A: (methanol) and mobile phase B: (0.1% formic acid water), at a ratio of A:B=45:55. The injection volume was 10μL and the flow rate was 1 mL min^-1^. The column temperature was kept at 25°C, running time of 60 minutes, and the UV detector’s wavelength of 330 nm.

#### Narirutin, hesperidin, neohesperidin, poncirin, hesperetin, eriocitrin, naringin, rhoifolin, vanillin and naringenin

Fresh peel and pulp samples (0.5 g) were dissolved in 1mL of 80% methanol water, and subjected to sonication at 300 W. The samples were then centrifuged at 4 °C, 12,000 rpm for 10 minutes. The supernatants were then filtered through a 0.22 μm filter membrane and analyzed using HPLC (Wufeng LC-100) following the same running conditions as those set for sinensetin, with UV detection at 283 nm.

#### Total phenols and flavonoids

First 0.1 g of fresh samples were obtained and ground on ice. Next, 1.5 mL of 60% ethanol was added and shaken for 2 hours at 60°C (when evaporated, it was diluted back to 1.5 mL with 60% ethanol). The detection method followed the instructions provided with the kit (Suzhou Geruisi Biotechnology Co., Ltd, for total phenols (G0117W), and flavonoids (G0118W) test kit respectively).

#### FRAP free radical clearance capacity

Firstly, 0.1g of fresh sample was weighed and ground in 1mL of 80% ethanol (provided). It was then transferred to a 2 mL centrifuge tube and subjected to ultrasonication at 60°C for 30 minutes at 200-300 W (with 5 min intervals of shaking and mixing). The resulting supernatant was subsequently centrifuged at 12,000 rpm for 10 minutes, cooled on ice, and assayed according to the provided.

#### ABTS free radical clearance ability and DPPH free radical clearing ability

A fresh sample weighing 0.1 g was obtained and uniformly ground with 1ml of methanol. Ultrasonic extraction was performed at 60°C, for 30 minutes (shaken and mixed every 5 minutes) using a 200-300W ultrasonic device. In case of any loss, the sample volume was adjusted with 80% methanol to 1mL. The samples were centrifuged at 12000 rpm for 10 minutes at room temperature to obtain the supernatant. Detection was carried out following the provided instructions with the ABTS free radical clearance ability (G0127W) kit of Suzhou Grace Biotechnology Co., Ltd. China, and DPPH free radical clearing ability (G0128W) test kit respectively).

#### Amino acid

0.1 g of fresh sample was weighed, to which 1mL of extract solution was added and homogenized at room temperature. Samples were then centrifuged at 12,000 rpm for 10 minutes at 4°C. The supernatant was placed on ice for detection, following the kit’s instructions for Amino acid (G0415W) of Suzhou Grace Biotechnology Co., Ltd. China.

#### Titratable acidity, TSS and vitamin C

The development of citrus fruits includes five stages: flowering, physiological fruit drop stage (0d-89d after flowering (DAF)), fruit enlargement stage (90-149 DAF), color transformation stage, and ripe stage (Singh et al., 2015). Citrus enters the fruit-expansion stage at 90 DAF, and the fruit diameter at 120 DAF is 3-4cm, with very little juice and cannot be detected. At 150 DAF, the fruit-expansion stage ends, and the fruit diameter is 5-6cm (Mo et al., 2019). Therefore, we start testing the main fruit quality assay of TSS, Acid and VC etc., from 150 DAF. The content of soluble solids (TSS) was measured by Atago digital practice saccharimeter (PAL-1, Atago, Japan). Similarly, titrable acids were determined by NaOH neutralization titration method (Titrette digital bottle neck titrator, Germany). The Vitamin C (VC) content (mg per 100mL by weight) was determined using 2,6-dichloroindophenol sodium titration method for VC content determination.

### Data analysis

Titratable acid content was calculated using citric acid with the formula = (V × M × 0.064) × 100, where ‘V’ representing the volume of 0.1 mol/L NaOH standard solution used to titrate the sample (mL), ‘M’ was the concentration of the NaOH standard solution (0.1 mol/L), and the factor ‘0.064’ represented the grams of citric acid required to neutralize 1 mL of 0.1 mol/L NaOH. The solid acid ratio (RTT) was determined by the ratio of soluble solid content to titratable acid content. The confidence test method of the binomial normal distribution was used to establish confidence intervals for the means of the main chemical substances. The Pearson correlation coefficient was employed to analyze correlations between the parameters of ‘Orah’ in different rootstock-scion combinations. Principal Component Analysis (PCA) was calculated using the ‘prcomp’ function within the ‘stat’ R package, and PCA results were visualized using the R package ‘factoextra’. All tests were conducted in triplicate, and the data was expressed as the mean ± standard deviation (SD) of the absolute content for each substance. Analysis of variance (Tukey method of ANOVA analysis) was performed using SPSS version 17.0, with statistical significance set at *P*<0.05.

## Results

### Influence of different rootstocks and development time on the quality of ‘Orah’

**Figure 1:**
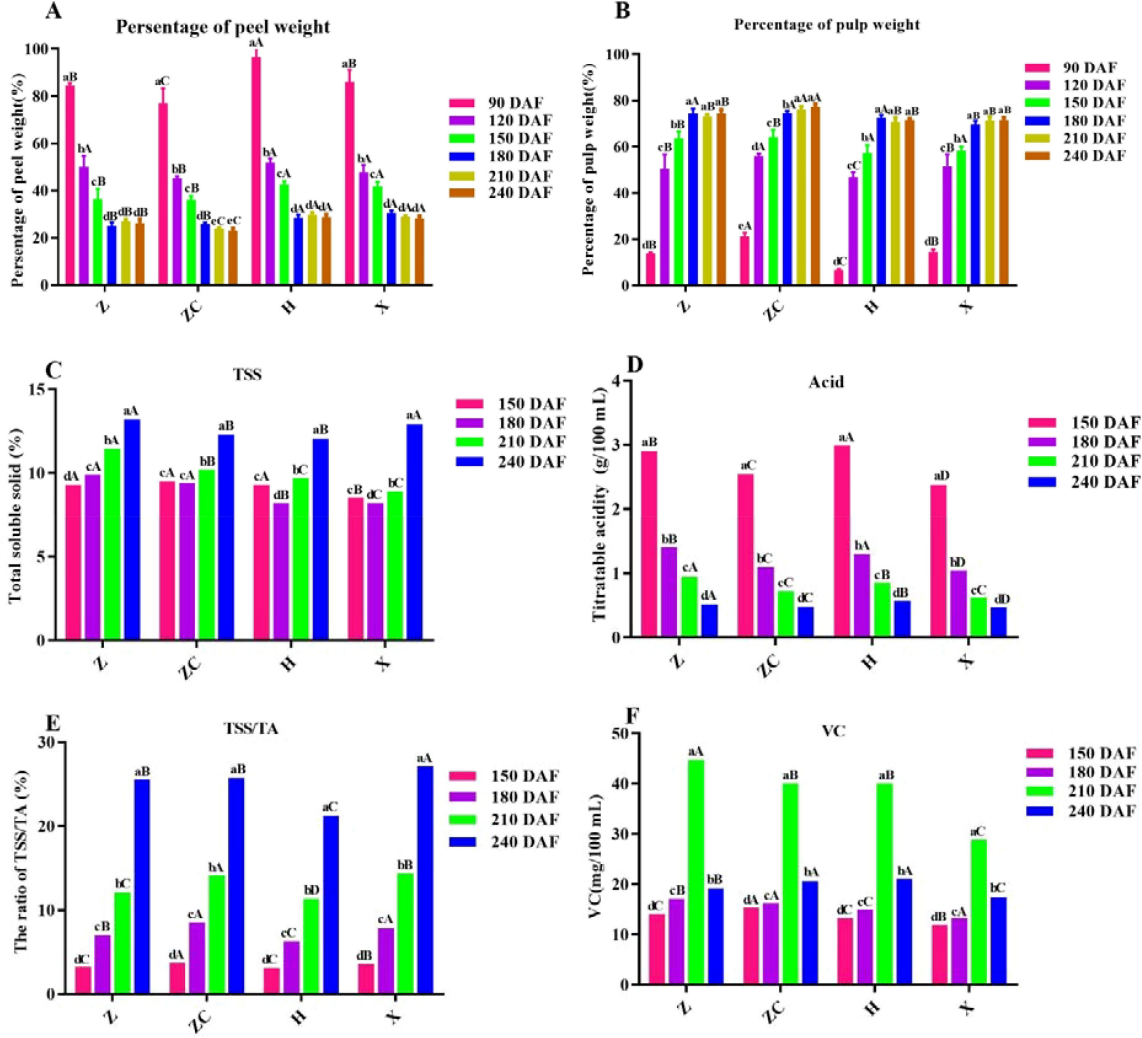
Effect of different rootstocks on fruit and quality of ‘Orah’. **(A)** persentage of peel weight. **(B)** persentage of pulp weight. **(C)** TSS. **(D)** Acid. **(E)** RTT. **(F)** VC. Z: *P. trifoliata* (L.) Raf, ZC: *C. sinensis* Osb.× *P. trifoliate* Raf. H: *C. reticulata* Blanco cv. Red tangerine, X: *C. junos* Sieb. ex Tanaka, 90, 120, 150, 180, 210, 240 DAF: Fruit at 90d, 120d, 150d, 180d, 210d, 240d after flowering. The samples in panels (**C–F**) were from the fruit pulp from 150 to 240 DAF. Different lowercase letters in the same rootstock at different times represented significant difference (*P*<0.05), while capital letters represent significant differences in the different rootstocks at the same time period (*P*<0.05).

In this study, we detected Indicators total soluble solids (TSS), titratable acid (TA), vitamin C, fruit weight and edible rate, for measuring the external quality of fruits. Based on the fruit development stage, we start testing the main fruit quality assay of TSS, Acid and VC etc., from 150 DAF. Results showed that the proportion of peel weight of ‘Orah’ on different rootstocks significantly decreased with the extension of developmental time (F= 92.13, *P*<0.05) in the early stage of fruit development. The proportion of peel weight of all rootstock-scion combinations tended to stabilize after 180 DAF except for ‘*ZC*’, while the proportion of pulp was opposite to the trend of peel proportion. As fruit development time extended, the proportion of fruit pulp increased significantly (F=56.63, *P*<0.05). The fruits ofon rootstock ‘*H*’ and ‘*X*’ were significantly heavier than those of ‘*Z*’ and ‘*ZC*’, while the pulp percentage of ‘*ZCR*’ was significantly heavier than the other threes (**Figures 1A**, **B**).

The TSS of the ‘*H’* and ‘*X’* were the lowest at 120 DAF, while that of the other rootstock continued to increase with fruits development. The TSS of the four rootstocks (‘*Z’*, ‘*ZC’*, ‘*H’*, ‘*X’*) reached its peak at 240 DAF, with values of 13.20%, 12.30%, 12.05%, and 12.90%, respectively. Therefore, the TSS of ‘*Z*’ was the highest in the same developmental period, significantly higher than the others (**Figure 1C**). And the trend of RTT was consistent with TSS, while that of total acid was opposite (**Figures 1C**–**E**).

The VC content under different rootstock grafting increased continuously with the fruit development. The highest content was observed at 210 DAF, with that of rootstock ‘*Z*’ (44.82 mg/100ml) was significantly higher than other rootstocks (*P*<0.05) (**Figure 1F**).

### Influence of different rootstocks and development time on the 3 types of total functional substances of ‘Orah’

**Figure 2:**
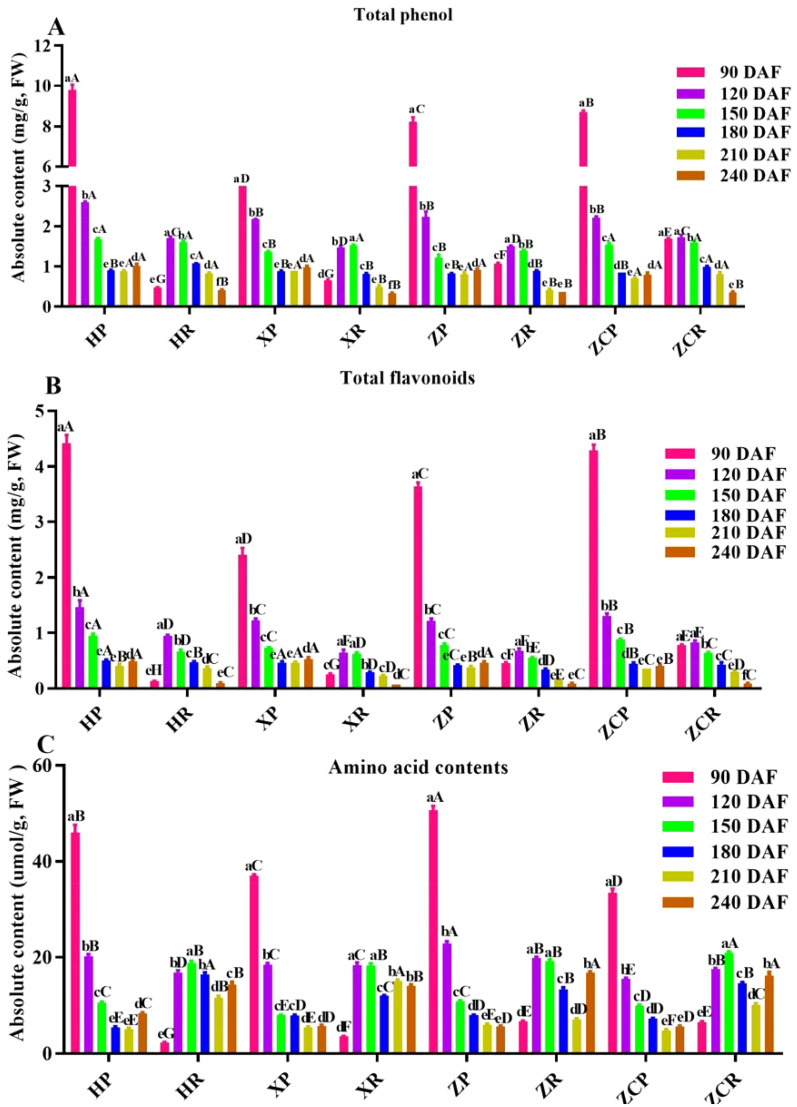
Changes in the content of functional substances in ‘Orah’ under grafting on different rootstocks. HP: Peel of ‘Orah’ grafted on *C. reticulata* Blanco cv. Red tangerine, HR: Pulp of ‘Orah’ grafted on *C. reticulata* Blanco cv. Red tangerine, XP: Peel of ‘Orah’ grafted on *C. junos* Sieb. ex Tanaka, XR: Pulp of ‘Orah’ grafted on *C. junos* Sieb. ex Tanaka, ZP: Peel of ‘Orah’ grafted on *P. trifoliata* (L.) Raf, ZR: Pulp of ‘Orah’ grafted on *P. trifoliata* (L.) Raf, ZCP: Peel of ‘Orah’ grafted on *C. sinensis* Osb.× *P. trifoliate* Raf, ZCR: Pulp of ‘Orah’ grafted on *C. sinensis* Osb.× *P. trifoliate* Raf. 90, 120, 150, 180, 210, 240 DAF: Fruit at 90d, 120d, 150d, 180d, 210d, 240d after flowering. All samples were fresh samples. Different lowercase letters in the same rootstock at different times represented significant difference (*P*<0.05), while capital letters represent significant differences in the different rootstocks at the same time period (*P*<0.05). **(A)** Total phenol. **(B)** Tatol flavonoids. **(C)** Tatole amino acid contents.

Phenolics are the most abundant secondary metabolites in plants. We detected the contents of total phenolic acid, total flavonoids and amino acid content in ‘Orah’ fruits under different development stages and rootstocks. Analysis of changes in the functional substances of the peel and pulp of ‘Orah’ grafted onto the four rootstocks, showed that the total phenol content of the peel in four rootstocks was relatively higher during the early stage of development (**Figure 2**). Among these, ‘*HP’* had the highest total phenolic content of 9.8 mg/g at 90 DAF, which decreased with the extension of the developmental period, reaching its the lowest point in the later stage. The total phenol content in ‘*HP’*, ‘*XP’*, ‘*ZP’*, and ‘*ZCP’* did not exhibit significant differences. However, except for ‘*ZCR’*, the total phenol content of the pulp in the other three combinations gradually increased with the extension of the developmental period. Among them, the rootstock *‘ZCR’* and *‘HR’* showed the highest levels at 120 DAF, with 1.7 mg/g and 1.51 mg/g, respectively (**Figure 2A**).

The changes in total flavonoid contents in the peel of the four rootstocks were consistent with the changes in total phenols. All decreased as the developmental period extended, with the highest total flavonoid content observed in ‘*HP’* at 4.42 mg/g. The highest total flavonoid content in the pulp of all four rootstocks was recorded at 120 DAF. Among these, the flavonoid content in ‘*HR’* had the highest value of 0.94 mg/g, which gradually decreased with the extension of developmental time (**Figure 2B**).

The results indicated that the changes in the total amino acid contents of the peel followed a pattern consistent with the changes in the total phenol content as the developmental period extended. The highest values were recorded in the early stages of development, gradually decreasing as time progressed. Of these, the total amino acid content of ‘*ZP*’ was the highest at 50.77 μmol/g. Except for the total amino acid content of ‘*HP*’ at its lowest point at 180 DAF, the rest were at their the lowest at 240 DAF. In the case of ‘Orah’ development, the total amino acid content in the pulp of ‘Orah’ grafted onto the four rootstocks showed an initial increasing trend, followed by a decreasing trend, with the highest levels at 150 DAF, 18.88 μmol/g, 18.29 μmol/g, 19.17 μmol/g and 20.97 μmol/g, respectively (**Figure 2C**).

### Influence of different rootstocks and development time on the 3 kinds of special functional substances of ‘Orah’

**Figure 3:**
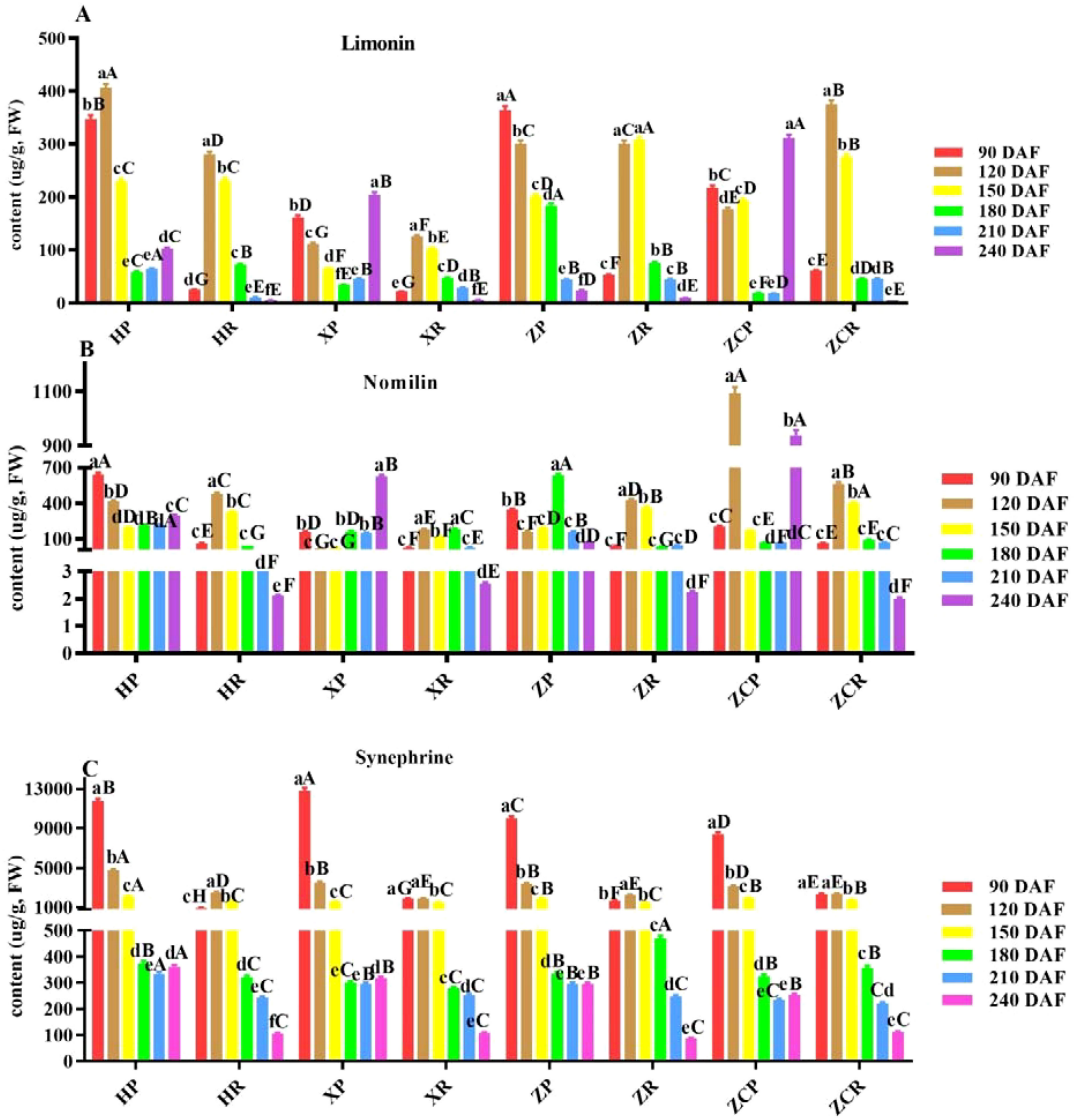
Changes of limonin, nomilin and synephrine content in ‘Orah’ under grafting of different rootstocks-scion combinations. HP: Peel of ‘Orah’ grafted on *C. reticulata* Blanco cv. Red tangerine, HR: Pulp of ‘Orah’ grafted on *C. reticulata* Blanco cv. Red tangerine, XP: Peel of ‘Orah’ grafted on *C. junos* Sieb. ex Tanaka, XR: Pulp of ‘Orah’ grafted on *C. junos* Sieb. ex Tanaka, ZP: Peel of ‘Orah’ grafted on *P. trifoliata* (L.) Raf, ZR: Pulp of ‘Orah’ grafted on *P. trifoliata* (L.) Raf, ZCP: Peel of ‘Orah’ grafted on *C. sinensis* Osb.× *P. trifoliate* Raf, ZCR: Pulp of ‘Orah’ grafted on *C. sinensis* Osb.× *P. trifoliate* Raf. 90, 120, 150, 180, 210, 240 DAF: Fruit at 90d, 120d, 150d, 180d, 210d, 240d after flowering. FW: Fresh weight. Different lowercase letters in the same rootstock at different times represented significant difference (*P*<0.05), while capital letters represent significant differences in the different rootstocks at the same time period (*P*<0.05). **(A)** Limonin. **(B)** Nomilin. **(C)** Synephrine.

Limonin is a secondary metabolite of triterpenoids and widely exists in citrus plants. As shown in **Figure 3A**; **Supplementary Figure S3**, the change in limonin content in the peel of different rootstocks varied with the fruit development stage. ‘*HP*’ exhibited the highest content of 405.5 μg/g at 120 DAF, while ‘*ZP*’ had the highest content of 364.49 μg/g at 90 DAF. The limonin content in the pulp initially increased and then decreased as the developmental time prolonged. It reached the lowest point at 240 DAF, with values of 2.11 μg/g, 2.54 μg/g, 2.23 μg/g, and 2.01 μg/g, respectively. Of these, ‘*ZR*’ and ‘*ZCR*’ reached their highest levels at 150 DAF (307.89 μg/g) and 120 DAF (374.86 μg/g), respectively (**Figure 3A**).

As the main limonin found in citrus fruits, nomilin has many pharmacological and health effects. Different rootstocks and developmental stages had different effects on the nomilin content in peel and pulp. The content of nomilin in ‘*HP*’ and ‘*XP*’ exhibited a trend of decreasing followed by increasing, and was significantly lower at 150 DAF compared to other developmental periods (F=12.53, *P*<0.05; F=32.21, *P*<0.05). The nomilin content of the ‘*ZCP*’ was significantly higher at 120 DAF (1093.59 μg/g) and 240 DAF (938.78 μg/g) respectively, compared to the other rootstocks at the same developmental stage. Howerver, ‘*ZP*’ reached its highest content at 180 DAF, significantly surpassing that of the other three rootstocks at the same developmental stage with 635.79 μg/g. The normilin content in the pulp of the four rootstocks initially increased, and then decreased, being significantly higher at 120 DAF, with ‘*ZCR*’ recording the highest at 565.59 μg/g (**Figure 3B**).

As an active ingredient with various biomedical functions, synephrine was found to be highly abundant in young citrus fruits. The synephrine content in the peel of the four rootstocks was significantly higher during the early stages of fruit development. ‘*XP*’ displayed the highest value of 12807.75 μg/g after 90 DAF. All values decreased to the lowest level at 210 DAF, with values of 334.87 μg/g, 293.83 μg/g, 294.95 μg/g, and 233.71 μg/g, respectively. The content of synephrine in both ‘*HR*’ and ‘*ZR*’ showed an initial increase, followed by a decrease during fruit development. Both reached their highest level at 120 DAF, with values 2484.56 μg/g and 2229.25 μg/g, respectively. ‘*ZR*’ and ‘*XR*’ exhibited consistent changes, with synephrine content decreasing at 120 DAF and being significantly lower at 240 DAF (F=45.76, *P*<0.05) (**Figure 3C**; **Supplementary Figure S4**).

### Influence of different rootstocks and development time on the seven kinds of phenolic acid of ‘Orah’

The dynamic changes of 7 main phenolic acids including protocatechuic acid, p-hydroxybenzoic acid, vanillic acid, caffeic acid, caprylic acid, ferulic acid and p-coumaric acid were quantitatively determined. The content of phenolic acids in young fruits grafting on different rootstocks was significantly different during the developmental stages.

**Figure 4:**
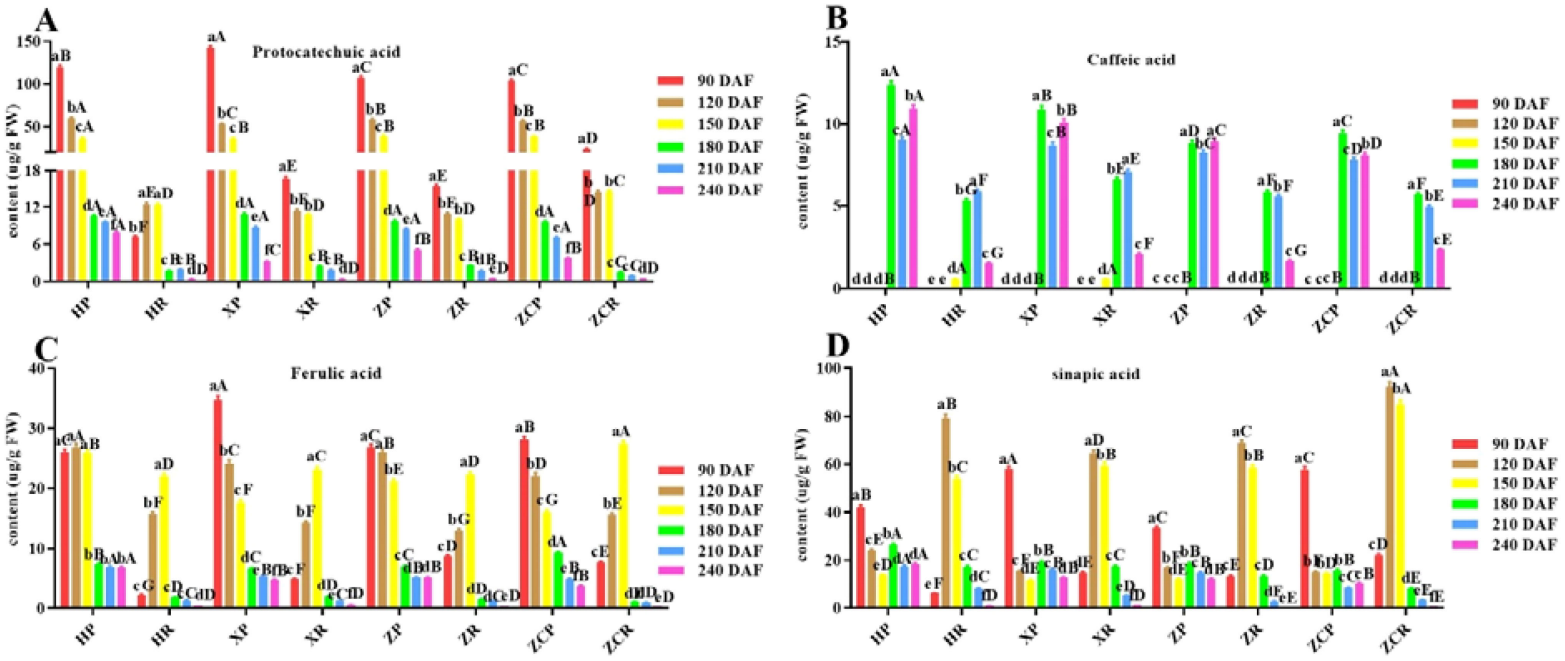
Changes in the content of four phenolic acid in ‘Orah’ under grafting on different rootstocks. HP: Peel of ‘Orah’ grafted on *C. reticulata* Blanco cv. Red tangerine, HR: Pulp of ‘Orah’ grafted on *C. reticulata* Blanco cv. Red tangerine, XP: Peel of ‘Orah’ grafted on *C. junos* Sieb. ex Tanaka,XR: Pulp of ‘Orah’ grafted on *C. junos* Sieb. ex Tanaka, ZP: Peel of ‘Orah’ grafted on *P. trifoliata* (L.) Raf, ZR: Pulp of ‘Orah’ grafted on *P. trifoliata* (L.) Raf, ZCP: Peel of ‘Orah’ grafted on *C. sinensis* Osb.× *P. trifoliate* Raf, ZCR: Pulp of ‘Orah’ grafted on *C. sinensis* Osb.× *P. trifoliate* Raf. 90, 120, 150, 180, 210, 240 DAF: Fruit at 90d, 120d, 150d, 180d, 210d, 240d after flowering. FW, Fresh weight. Different lowercase letters in the same rootstock at different times represented significant difference (*P*<0.05), while capital letters represent significant differences in the different rootstocks at the same time period (*P*<0.05). **(A)** Protocatechin. **(B)** Caffeic acid. **(C)** Ferulic acid. **(D)** Sinapic acid.

As shown in **Figure 4**; **Supplementary Figures S1**, **S6**, the four phenolic acids include protocatechin, caffeic acid, ferulic acid, sinapic acid had higher content and more consistent. The content of protocatechins in peel was higher than that in pulp at all developmental stages, and reached the maximum at 90 DAF (F=45.87, *P*<0.05). It gradually decreased as the developmental time extended. Of these, ‘*XP’* and ‘*ZCR’* exhibited the highest values (**Figure 4A**). Caffeic acid was detected in all four rootstocks after 180 DAF, with ‘*HP*’ having the highest content at 12.35 μg/g, surpassing that of the other rootstocks across all development stages (**Figure 4B**). The content of ferulic acid in the peel of the four rootstocks showed the highest level in the early stage of development, which continued to decrease as the developmental time extended. The highest ferulic acid content was measured in the pulp at 150 DAF, at 22.05μg/g, 23.13 μg/g, 22.27 μg/g, and 27.45 μg/g, respectively (**Figure 4C**).

The sinapic acid content in pulp was significantly higher than that in peel from 120 to 150 DAF, reaching its peak at 120 DAF. ‘*ZCR’* exhibited a significantly higher content compared to the remaining three rootstocks, with a value of 92.37 μg/g. In the peel, it was significantly higher within 90 DAF. Among these, ‘*XP’* exhibited a significantly higher sinapic acid content at 57.86 μg/g (**Figure 4D**). The content of other three kinds phenolic acids (p-hydroxbenzoic, vanillic acid and p-Coumaric acid) in the peel and pulp of ‘Orah’ grafted on four rootstocks was below 12 μg/g, which increased at 180 DAF (**Supplementary Figure S1**).

### Influence of different rootstocks and development time on the 13 kinds of flavonoids of ‘Orah’

**Figure 5:**
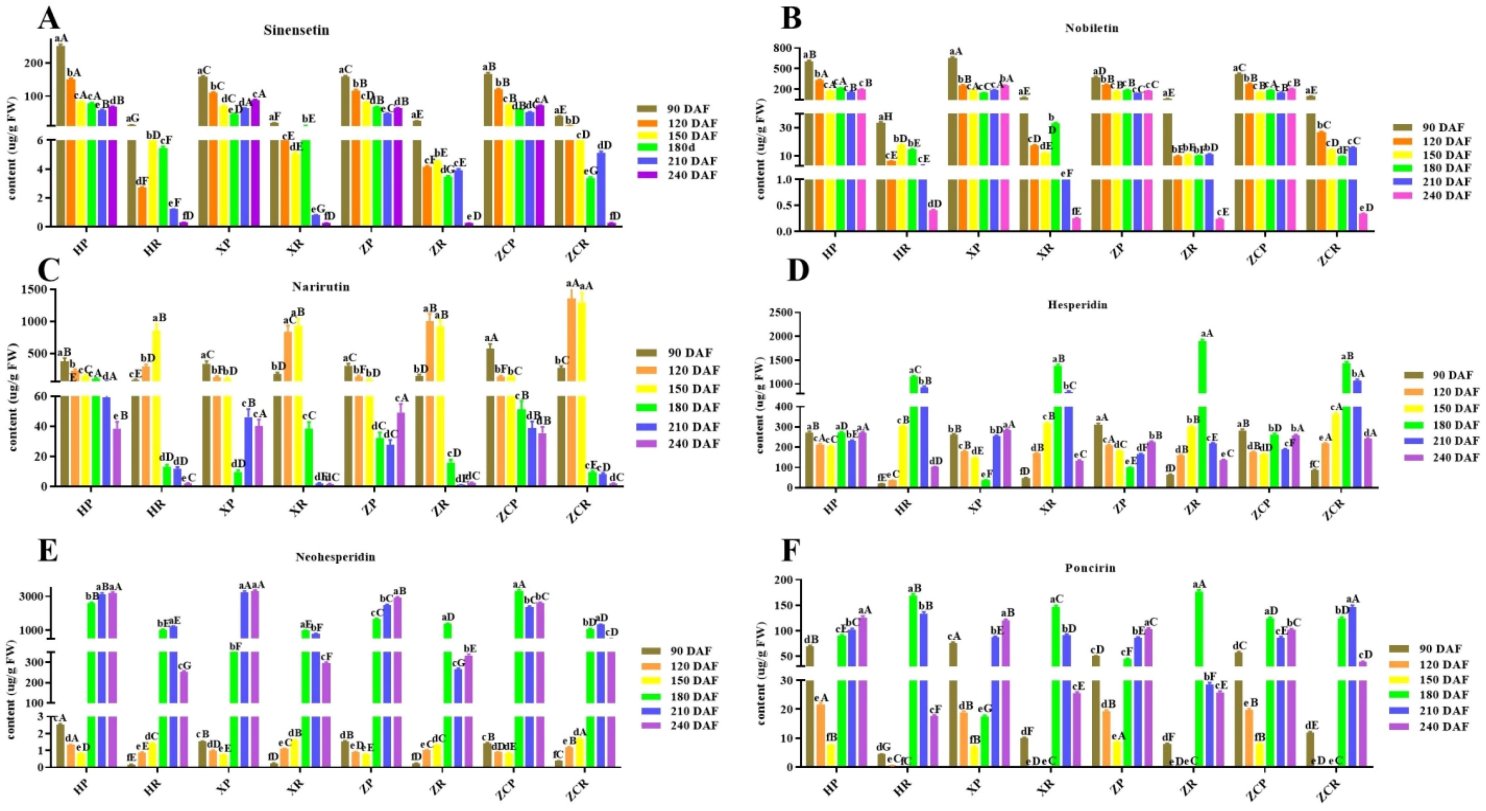
Changes in the content of six main flavonoids in ‘Orah’ under grafting on different rootstocks. HP: Peel of ‘Orah’ grafted on *C. reticulata* Blanco cv. Red tangerine, HR: Pulp of ‘Orah’ grafted on *C. reticulata* Blanco cv. Red tangerine, XP: Peel of ‘Orah’ grafted on *C. junos* Sieb. ex Tanaka,XR: Pulp of ‘Orah’ grafted on *C. junos* Sieb. ex Tanaka, ZP: Peel of ‘Orah’ grafted on *P. trifoliata* (L.) Raf, ZR: Pulp of ‘Orah’ grafted on *P. trifoliata* (L.) Raf, ZCP: Peel of ‘Orah’ grafted on *C. sinensis* Osb.× *P. trifoliate* Raf, ZCR: Pulp of ‘Orah’ grafted on *C. sinensis* Osb.× *P. trifoliate* Raf. 90, 120, 150, 180, 210, 240 DAF: Fruit at 90d, 120d, 150d, 180d, 210d, 240d after flowering. FW, Fresh weight. Different lowercase letters in the same rootstock at different times represented significant difference (*P*<0.05), while capital letters represent significant differences in the different rootstocks at the same time period (*P*<0.05). **(A)** sinensetin. **(B)** Nobiletin. **(C)** Narirutin. **(D)** Hesperidin. **(E)** Neohesperidin. **(F)** Poncirin.

Thirteen vital flavonoids (sinensetin, nobiletin, hesperidin, narirutin, tangeretin, eriocitrin, naringin, rhoifolin, vanillin, naringenin, hesperetin, neohesperidin and poncirin) in citrus were selected for detection in this study to provide reference for flavonoid utilization after fruit thinning. Among them, six main substances were selected for analysis according to the principle similar with phenolic acids (**Figure 5**; **Supplementary Figures S2**, **S5**). Under the same rootstock, the sinensetin content in the peel was generally higher than that in the pulp during the same period. The sinensetin content in the peel of ‘Orah’ decreased as the fruit developed. Except for ‘*XP’*, the content in the peels had lower levels at 210 DAF (58.52 μg/g, 47.92 μg/g, 51.72 μg/g). The sinensetin level of ‘*XR’* and ‘*ZCR’* was significantly higher than that of ‘*HR*’ and ‘*ZR*’. The sinensetin content in pulp was the lowest at 240 DAF, measuring 0.29 μg/g, 0.26 μg/g, 0.25 μg/g, and 0.27 μg/g, respectively (**Figure 5A**).

The content distribution of nobiletin in peel and pulp was similar to that of sinensetin. Nobiletin in the peel and pulp of the four rootstocks showed higher levels during the early stage, which decreased to the lowest during later developmental stages. The ‘*X*P’stock had the highest content of 110.95 μg/g at 90 DAF. Except for ‘*ZR’*, the level of nobiletin in the pulp of the other grafts was initially lower, then increased and and finally decreased (**Figure 5B**).

The narirutin content in ‘*HP’* and *‘ZP’* decreased with the extension of developmental time, stabilizing at 150 DAF. That of ‘*HR’* and ‘*ZR’* was significantly lower at 240 DAF than in other developmental periods, with recorded levels of 2.06 μg/g, 2.0 μg/g. ‘*XP’* and ‘*ZP’* had significantly higher at 90 DAF than in other developmental stages, while that of *XR* and *ZR* was significantly higher levels at 150 DAF compared to other developmental stages (F=37.82, *P*<0.05). The narirutin content of *ZCR* was significantly higher at 120 and 150 DAF than other rootstock-scion combinations, with recorded values of 1356.04 μg/g and 1298.85 μg/g, respectively (**Figure 5C**).

The content of hesperidin in the peel of the four rootstocks was significantly higher during the early stage of development than that in the late stages. ‘*ZP’* showed the highest value at 311.81 μg/g. The hesperidin content in the pulp of the four rootstocks reached its peak at 180 DAF (1149.22 μg/g, 1382.42 μg/g, 1894.64 μg/g, and 1439.6 μg/g, respectively). The content was lowest at 90 DAF, with ‘*HR’* (20.49 μg/g) significantly lower than the other three rootstocks (**Figure 5D**).

The neohesperidin content in the peel and pulp of the four rootstocks increased significantly after 150 DAF. Except for ‘*ZCP’*, the other rootstocks showed higher levels at 240 DAF, measuring 3192.35 μg/g, 3306.55 μg/g, and 2904.64 μg/g, respectively. The neohesperidin content in ‘*HR’* and ‘*ZCR’* reached the highest at 210 DAF (1217.67 μg/g and 3192.35 μg/g, respectively). While ‘*XR’* and ‘*ZR’* reached the highest level at 180 DAF (981.84 μg/g, and 1387.94 μg/g, respectively) (**Figure 5E**).

Except for the ‘*ZCP*’, the poncirin contents in the peels of three rootstocks were highest during the late developmental phase, measuring 125.65 μg/g, 119.79 μg/g, and 102.96 μg/g, respectively. The levels in the pulp were highest in the four rootstocks at 150 DAF, measuring 168.43 μg/g, 146.35 μg/g, 175.84 μg/g, and 123.78 μg/g, respectively (**Figure 5F**).

The tangeretin, hesperetin, eriocitrin, naringin, rhoifolin, vanillin and naringenin content in the peel and pulp of the four rootstocks was below 180μg/g (**Supplementary Figure S2**). There were no significant differences in multiple flavonoids content in the peel and pulp of the four rootstocks at the same developmental time period (**Supplementary Figures S2A–C**, **S2F, G**). Even more interestingly, with the exception of vanillin, the content of the other six kinds of flavonoids was higher in the peel and lower in the pulp (**Supplementary Figure S2**).

### The antioxidant activity in ‘Orah’fruits under four rootstock-scion combinations and development time

**Figure 6:**
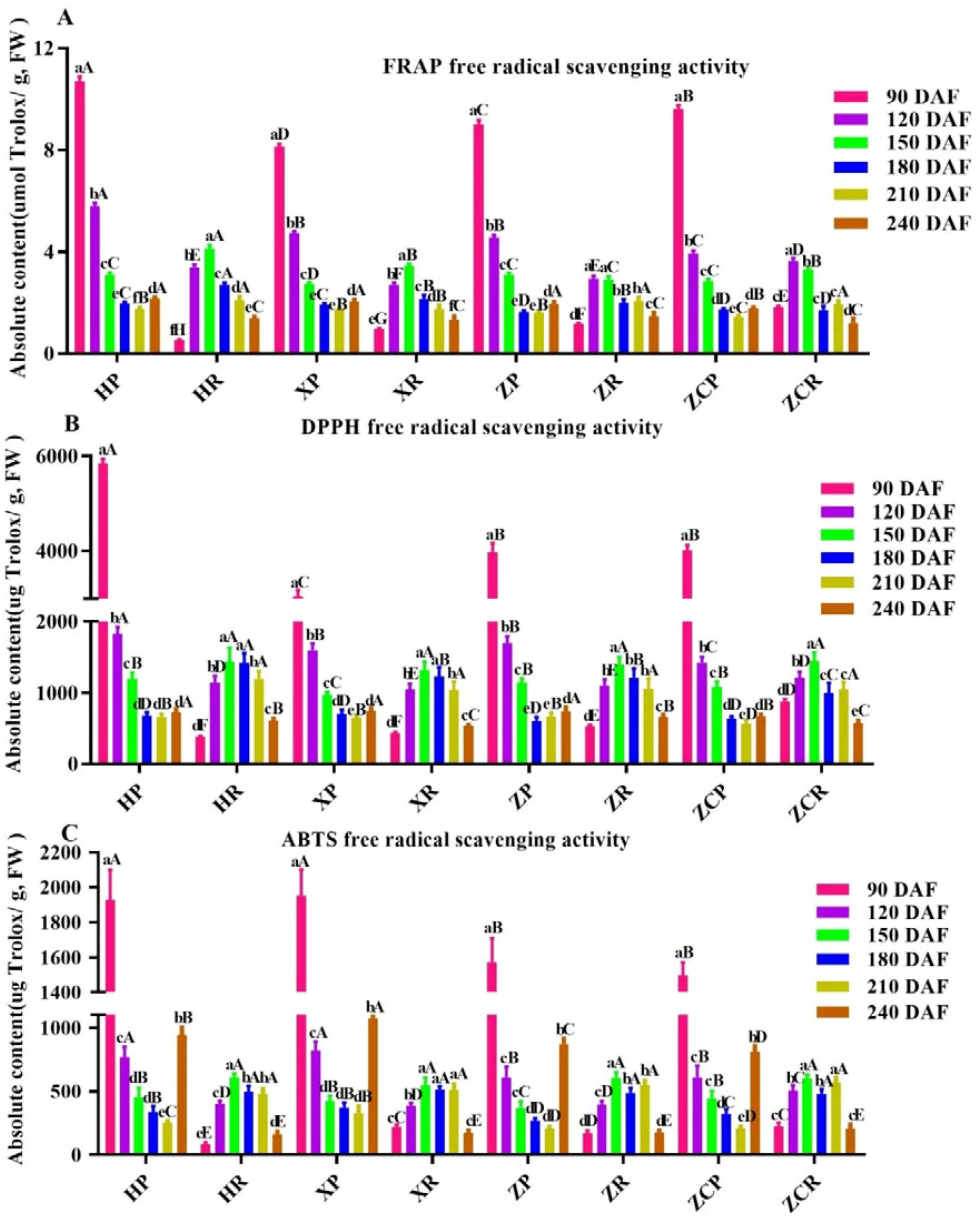
Changes in antioxidant capacity of ‘Orah’ under under grafting on different rootstocks. HP: Peel of ‘Orah’ grafted on *C. reticulata* Blanco cv. Red tangerine, HR: Pulp of ‘Orah’ grafted on *C. reticulata* Blanco cv. Red tangerine, XP: Peel of ‘Orah’ grafted on *C. junos* Sieb. ex Tanaka,XR: Pulp of ‘Orah’ grafted on *C. junos* Sieb. ex Tanaka, ZP: Peel of ‘Orah’ grafted on *P. trifoliata* (L.) Raf, ZR: Pulp of ‘Orah’ grafted on *P. trifoliata* (L.) Raf, ZCP: Peel of ‘Orah’ grafted on *C. sinensis* Osb.× *P. trifoliate* Raf, ZCR: Pulp of ‘Orah’ grafted on *C. sinensis* Osb.× *P. trifoliate* Raf. 90, 120, 150, 180, 210, 240 DAF: Fruit at 90d, 120d, 150d, 180d, 210d, 240d after flowering; FW, Fresh weight. Different lowercase letters in the same rootstock at different times represented significant difference (*P*<0.05), while capital letters represent significant differences in the different rootstocks at the same time period (*P*<0.05). **(A)** FRAP free radical scavenging capacity. **(B)** DPPH free radical scavenging activity. **(C)** ABTS free radical scavenging activity.

In order to evaluated the antioxidant capacity *in vitro* of citrus, we employed the DPPH, FRAP and ABTS method, which were relatively simple and feasible. The results showed that the antioxidant capacity of peel was significantly higher than that of pulp. FRAP free radical scavenging capacity in the peels was the highest at 90 DAF (**Figure 6**). Among these, ‘*HP*’ showed the strongest antioxidant capacity at 10.7 μmol Trolox/g, which then decreased with the extension of developmental time, reaching the lowest point at 210 DAF (F=234.21, *P*<0.05). The FRAP free radical scavenging capacity in the pulp was initially higher which later dropped. Overall, the antioxidant was the strongest at 150 DAF, except for ‘*ZC*’. The highest recorded value was for ‘*HR*’ at 4.12 μmol Trolox/g (**Figure 6A**).

The DPPH free radical scavenging activity in the peel showed an initial peak followed by a gradual decline. However, the scavenging activity in the pulp presented tendency of rising initially to the peak and then falling, where it was higher initially and then decreased, with the highest values at 150 DAF (1439.49 μg Trolox/g, 1317.95 μg Trolox/g, and 1395.04 μg Trolox/g, respectively), except for ‘*ZCR*’ (**Figure 6B**).

Results indicated that changes in ABTS free radical scavenging activity of ‘Orah’ grafted onto the other three rootstocks were consistent with the changes in DPPH free radicals activity, except for ‘*ZCR*’ (**Figure 6C**). Of these, ‘*XR*’ recorded the highest value at 1927.39 μg Trolox/g, while *HR*’ had the highest value at 90 DAF at 607.81 μg Trolox/g (**Figure 6C**).

### Correlation analysis of main functional substances in the fruits of ‘Orah’ under four rootstock-scion combinations and development time

Considering the dynamic changes of functional substances during different developmental periods of citrus fruits, the functional substances and antioxidant activities of each developmental period were individually subjected to principal component analysis and subordinate function analysis in this study, which could more scientifically reflect the optimal young fruit thinning time. The result showed a significant positive correlation (F=45.85, *P*<0.01) among the activities of antioxidant enzymes activities, as demonstrated by the FRAP, DPPH, and ABTS values (**Supplementary Table S1**). Additionally, a significant positive correlation was observed among three limonins (linimon, nomilin, and synephrine) and three phenolic acids (protocatechuic, sinapic, and ferulic) (F=37.92, *P*<0.01). A notable correlation was also found among the other substances (F=65.36, *P*<0.05), including 6 flavonoids. However, no significant correlations were observed between protocatechuic, sinenselin, narirutin, or between nobiletin, hesperidin, and the 3 antioxidant enzymes (FRAP, DPPH, and ABTS), as well as between nomilin, sinenselin, nobiletin hesperidin, or between poncirin and FRAP, DPPH, sinenselin, and nobiletin. Yet, a significant correlation was identified among the other substances (F=103.25, *P*<0.05).

The results of the principal component analysis revealed a high level of consistency in the functional substances of the ‘Orah’ grafted onto the four rootstocks (**Supplementary Table S2**). It is evident that the comprehensive evaluation outcomes from the principal component analysis and membership function for the functional substances of the fruits in the four rootstock-scion combinations were highly consistent. This consistency underscores the validity of the methods and indicators selected for this study.

**Figure 7:**
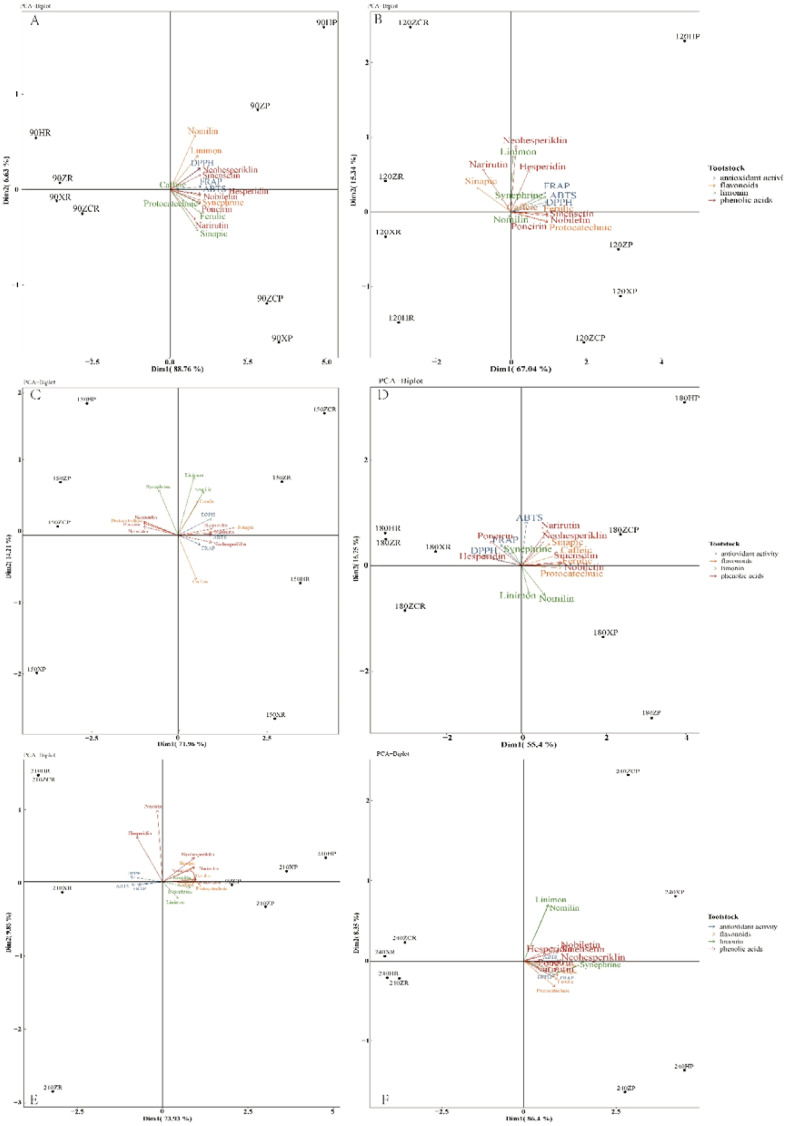
Principal component analysis (PCA) of the 16 parameters analyzed for ‘Orah’. **(A)**, fruit at 90d after flowering. **(B)**, fruit at120 d after flowering. **(C)**, fruit at 150d after flowering. **(D)**, fruit at 180d after flowering. **(E)** fruit at 210d after flowering. **(F)**, fruit at 240d after flowering. Antioxidant activity, limonin, flavone compounds and phenolic compounds are coloured as categories as specified in the figure legend.

To further analyze the variations among the tested rootstocks in terms of metabolite production, a multifactorial analysis was performed using data collected over two years. For the phenotyping (**Figure 7A**) conducted on July 8, 2020 (fruit at 90 DAF), the first two principal components accounted for 95.39% of the cumulative phenotypic variability (Dim1 = 88.76%, Dim2 = 6.63%). Dim1 primarily correlated with the quantity of functional metabolites produced (with Dim1>0 indicating higher production, as observed for nomilin, sinapic and neohesperiklin; **Figures 4**–**6**). Dim2 allowed a more precise differentiation based on various classes of metabolites. Rootstocks exhibiting high production of limonin, flavonoids, and antioxidant activity such as 1*ZP* and 1*HP* clustered in the upper-right quadrant (Dim1>0 and Dim2>0). Meanwhile, samples showing higher synthesis of phenolic compounds were situated (**Figure 7A**) in the lower-right quadrant (Dim1<0 and Dim2>0).

For the analysis performed 120 DAF, the first two principal components explained 82.38% of the total phenotypic variability. However, poncirin, sinentin, protocatechuic, nobiletin, and ferulic accessions showed a showing high accumulation of metabolites characterized by negative Dim1 values (**Figures 5**, **6**, **7B**). Moreover, at 120 DAF, the loadings related to the antioxidant activity exhibited strong consistency, all projecting towards the same PCA quadrant (upper-right). Specifically, ‘*HP*’ exhibited both high antioxidant capacity and high flavonoid content (**Figure 7B**).

Further analysis revealed that after 150 DAF, the first two principal components explained 86.17% of the cumulative phenotypic variability (Dim1 = 71.96%, Dim2 = 14.21%). Except for caffeic and neohesperiklin, the rest of the flavonoids and phenolic acids were characterized by positive Dim1 values (**Figures 5**, **7C**). Notably, synephrine stood out, being orthogonally projected compared to the linimon and nomilin (**Figure 7C**). Interestingly, ‘*ZR*’ and ‘*ZCR*’ exhibited neohesperiklin, linimon, nomilin, sinapic, and ferulic contents, clustering in the upper-right quadrant (Dim1>0 and Dim2>0) as portrayed (**Figures 5**, **6**, **7C**).

The first two principal components explained 72.15% of the cumulative phenotypic variability (Dim1 = 55.40%, Dim2 = 16.75%). Dim1 was mainly associated with the quantity of functional metabolites produced and strongly activated the antioxidant enzymes (with Dim1>0 associated with higher production, as observed for 3 phenolic acids and 7 flavonoeds excluding protocatechuic; **Figures 5**–**7D**). Notably, synephrine was characterized by a backward projection compared to the linimon and nomilin (**Figure 7D**). Rootstocks characterized by high production of flavonoids (narirutin, neohesperiklin, sinensetin, and nobiletin), phenolic acids (ferulic and caffeic) and a high ABTS free radical clearance ability (**Figure 7D**) in ‘180*ZCP*’ and ‘180*HP*’ clustered in the upper-right quadrant (Dim1>0 and Dim2>0).

For the analysis performed 210 DAF, the first two principal components (**Figure 7E**) explained 83.79% of the total phenotypic variability (Dim1 = 73.93%, Dim2 = 9.86%). Dim2 facilitated a more precise differentiation based on antioxidant activity (Dim2<0, **Figure 7E**). Notably, the upper-left quadrant (Dim1<0 and Dim2>0) exhibited high contents of poncirin and hesperidin (**Figures 6**, **7E**). Moreover, at 210 DAF, the loadings related to the ‘*XP*’ and ‘*HP*’ projected towards the same PCA quadrant (upper-right) (**Figure 7E**).

The first two principal components explain 94.75% of the cumulative phenotypic variability (Dim1 = 86.4%, Dim2 = 8.35%). The functional compounds and antioxidant activities were mainly located on the right of the PCA quadrant. Rootstocks characterized by high production of limonin, nomilin, flavonoids (sinensetin, nobiletin and hesperidin), as well as high ABTS free radical clearance ability in ‘240*XP*’ and ‘240*ZCP*’, clustered in the upper-right quadrant (Dim1>0 and Dim2>0), as shown (**Figures 4**, **6**, **7F**). Samples demonstrating higher synthesis of phenolic compounds (**Figure 6F**) were plotted in the lower-right quadrant (Dim1<0 and Dim2>0).

## Discussion

Rootstocks can significantly influence both internal and external quality parameters of citrus fruits, including bioactive compounds, shortened juvenile period, and improved fruit quality (Saini et al., 2019). Choosing rootstocks is an important tool for adapt crops to adverse environmental conditions or biotic (diseases, pests) and abiotic (drought, alkalinity, cold, etc) stresses, in order to improve fruit quality (Morales et al., 2023). In this study, we evaluated the impact of rootstocks of ‘*H*’, ‘*X*’, ‘*Z*’ and ‘*ZC*’ rootstocks on the quality and functional content of ‘Orah’ citrus. The results showed that the rootstocks affected the sugar and acid contents of the scion fruits. Similarly, the rootstock of the ‘sour’ orange increased the total soluble solids (TSS) and titratable acidity (TA) levels of different citrus varieties grafted onto the rootstock, thereby delaying fruit maturity in comparison to other rootstocks (Bassal, 2009; Singh et al., 2020). The effects of different rootstocks on ‘Marsh’ grapefruit indicated that citrus fruits grafted onto ‘*ZC*’ had the lowest TA content, while citrus fruits grafted onto the putative hybrid of *Citrus aurantium* had the lowest TSS content (McCollum and Bowman, 2002). It was also observed that the ‘*ZC*’ rootstock could reduce the TSS of scion (Clementine) fruits (Hussain et al., 2013). However, the effects of the four rootstocks on ‘Marisol’ clementine (*H*), with fruits grafted onto ‘*ZC*’, showed higher TSS and TA than those grafted onto ‘Sour’ orange (Bassal, 2009). Our results showed that the TSS and VC content of these four rootstocks continued to increase with the extension of developmental time, reaching the highest levels at maturity. In contrast, the trend of TA change was opposite, presumably due to environmental factors. The TSS/TA ratio serves as an important indicator of both citrus commercial maturity and sensory maturity (Goldenberg et al., 2018). Vitamin C is considered one of the most important nutrients in citrus fruits. It has been reported that rootstocks affect the production of vitamin C in citrus fruits (Magwaza et al., 2017). Studies have shown that rootstock ‘*H*’ (Khalifa and Hamdy, 2015) and ‘*ZC*’ (Cardeñosa et al., 2015) could not increase the VC content of citrus fruits. The rootstock ‘*Z*’ having was significantly higher content than the rest of the rootstock-scion combinations in our study. Qureshi et al. had also found that rootstock ‘*Z’* was also superior to rootstock ‘*ZC*’ in improving the VC content of scion ‘Kinnow’ fruit (Qureshi et al., 2021). Our results indicated that all four rootstocks were suitable for ‘Orah’, with the ‘*X*’ being the optimal rootstock in this study.

Citrus fruits serve as natural sources of antioxidants and bioactive compounds, including organic acids, phenolic compounds, and flavonoids (Sicari et al., 2017). Phenolic compounds not only enhance antioxidant capacity, inhibit some pests feeding, but also contribute to reduced risks of cardiovascular disease and certain cancers (Lattanzio et al., 2006; Sdiri et al., 2014). Studies have shown that the ‘Tavarini’ rootstock affects fruit quality through interactions between water, soil nutrients, and the synthesis of compounds like phenolic compounds (Tavarini et al., 2011). In our study, multiple phenols were identified in ‘Orah’ grafted onto the four rootstock-scion combinations, including the protocatechins, caffeic acid, ferulic acid, and sinapic acid, among others, most of which be used as pharmaceuticals. Changes in polyphenol content followed a trend consistent with ‘Kinnow’ citrus grafted on different rootstocks (Singh et al., 2020). We found that ferulic acid and protocatechins were abundant in the pre-development phase (before 150 DAF), while sinapic acid was higher between 120-150 DAF. Results showed that sinapic acid and protocatechins were widely present in citrus (Nićiforović and Abramovič, 2014). A small amount of caffeic acid was produced in ‘Orah’ grafted onto the four rootstocks after 180 DAF. Importantly, ‘*ZC*’ stood out as the best rootstock for ‘Orah’ in terms of phenolic compounds. This suggests that ‘*ZC*’ was selected as being tolerant, which might trigger the tree’s antioxidant defense system and, leading to higher phenol accumulation and providing raw materials for pharmaceuticals (Zandalinas et al., 2017). More importantly, our principal component analysis of the fruits after 90 DAF and 150 DAF also confirmed the hypothesis (**Figures 7A**, **C**).

Flavonoids are the primary class of phenolic substances that have granted special attention due to their potential health benefits. Studies have enlightened that ‘Osbeck’ citrus fruit grafted onto the rootstock *C. sinensis* [L.] exhibited flavonoid biosynthetic activity, while scion leaves on ‘Cleopatra mandarin’ citrus demonstrated stronger flavonoid biosynthesis activity (Souza et al., 2017). Our results indicate that the qualitative characteristics of ‘Orah’ grafted onto the four rootstocks were similar with significant changes in contents between 500-2000 μg/g. Flavonoid content in different species varied greatly with developmental time extension. The total flavonoid content of fruits grafted onto ‘*H*’ was higher than other rootstocks. Other studies have also reported higher phenolic content in lemon fruits grafted onto ‘sour’ orange rootstock (*Citrus aurantium* L.), while lemons on high-yielding ‘Volkamer’ rootstock have lower phenolic content (Emmanouilidou and Kyriacou, 2017). Overall, the total concentration of phenolic compounds in the four rootstocks ranged between 15 to 115 mg/100g fresh weight. These results align with previous research trends for other cultivars grafted onto the same four rootstocks (Li et al., 2022). Our research results also confirmed that the content of flavonoids and antioxidant capacity have higher content during the young fruit stage (90 and 150 DAF) especially in the ‘*Z*’. From the perspective of functional substances utilization, the ‘Orah’ fruit of 90 DAF and 150 DAF were the optimal thinning times. In practical production, fruit farmers often prethin many times from July to September. Previous study has also found that flavonoids and antioxidant capacity in grapefruit and lemon also exhibit higher levels at 90 DAF (Castillo et al., 1992; Zhu et al., 2020). Therefore, our results provide guidance for the fruits thinning, which could be carried out twice at 90 DAF and 150 DAF, reducing labor costs.

Citrus scion on the four rootstocks suggested that ‘Var. Shelmahalleh’ (*C. sinensis*) had the highest hesperidin content in the peel, followed by ‘Swingle’ citrus (Hemmati et al., 2018). In our study, ‘*Z*’ exhibited the highest hesperidin content among the four rootstock-scion combinations, followed by ‘*ZC*’, which is consistent with the research findings of Babazadeh-Darjazi et al (Babazadeh-Darjazi, 2018). Another study discussed the influence of six rootstocks on ‘Kinnow’ *Citrus Lour*. x *Citrus Deliciosa* Ten. & Pasq.) (Singh et al., 2020), and found that the highest levels of flavanones (hesperidin, naringin, pectin rutin, naringenin, and neocitric acid) and dihydroxy B-faravanol (rutin and quercetin) were induced by the ‘Sour’ orange rootstock. In comparision with the rootstocks ‘*H*’, ‘*Z*’ and ‘*ZC*’, the fruits of rough lemon rootstocks contained higher levels of naringenin, hesperitin, and eriodyctol (Feng et al., 2018). Most of them can be used as pest control agents and raw materials for treating human diseases (Lattanzio et al., 2006; Khalifa and Hamdy, 2015; Shi et al., 2021). Our study also identified naringenin and hesperetin as functional constituents of the above rootstocks, with naringenin content significantly lower than that of hesperidin. Additionally, consistent with the previous study (Zhu et al., 2020), the hesperidin and neohesperidin contents were significantly higher than other flavonoids in the later stage of development (after 180 DAF). Overall, our results showed that the ‘*Z’* rootstock induced higher flavonoid content in both peel and pulp.

In addition to phenolic compounds, citrus also contains various functional substances, such as synephrine, which is the main alkaloid in *Citrus aurantium* L. extracts and the main component of traditional Chinese medicine for treating indigestion and pharyngeal diseases (Arbo et al., 2008). And citrus fruits using ‘*Z*’ rootstocks can produce synephrine (Zhang et al., 2011). The content of synephrine in both rootstocks ‘*H*’ and ‘*Z*’ showed an initial increase, followed by a decrease during fruit development in our study. Citrus limonoids, the precursor for limonoids, are ascribed to fruit bitterness, but also possess favorable health properties including anti-oxidative, hypocholesterolemic and anti-carcinogenic effects (Manners, 2007). Interestingly, rootstocks could affect their content in citrus fruits. A study has found that the fruits of ‘*ZC*’ rootstocks could produce limonin and normilin (Tietel et al., 2020). Compared with other rootstocks, we also found that the content of normilin was highest in the early stage of fruit development (120 DAF) of ‘*ZC*’, while limonin performed the best on ‘*H*’ rootstock.

Rootstocks also play a role in regulating the antioxidant activity of scion fruits. Our results indicate that free radical scavenging activity in ‘Orah’ grafted onto the four different rootstocks was higher in the early stages of development compared to later stages, with higher activity observed in the peels compared to the pulp. In terms of antioxidant capacity, the ‘*H*’ rootstock was optimal. A study also indicated that grafting FA ‘Cleopatra mandarin’ on ‘*ZC*’. increased the concentration of several bioactive compounds, such as VC, diphenylamine, and naringenin, thus enhancing the antioxidant capacity (DPPH) of citrus fruits (Li et al., 2022).

## Conclusions

The citrus variety ‘Orah’ has garnered consumer interest due to its high yield and rich nutritional components. In this study, we determined the fruit quality, functional constituents and antioxidant capacity in both peel and pulp of ‘Orah’ at 90, 120, 150, 180, 210 and 240 DAF. Results showed that the content of functional constituents was highest 150 DAF, indicating that it could utilized as a suitable fruit thinning period. In terms of TSS, rootstock ‘*Z’* was demonstrated the highest performance. With regarding to TSS/TA, rootstock *‘X*’ emerged as the optimal choice. Then, ‘*ZC’* proved to be the most effective for VC. The highest levels of phenolic acid and flavonoids were observed at 90 DAF with the rootstock ‘*H*’ showing superior results, suggesting its potential use as an additional fruit thinning period. Moreover, high levels of sinensetin, nobiletin, protocatechins and ferulic acid were detected during the early stage of development. Furthermore, rootstock ‘*H’* and ‘*X’* exhibited the highest levels of functional substances, noteworthy some functional substances displayed higher content in the pulp than that in the peel. Our results also indicated that the greatest antioxidant capacity were recorded in the early stage of development, with the rootstock ‘*H’* identified as superior in this regard. In summary, our results suggested that rootstocks could significantly affect the functional constituents accumulating and antioxidant capability in fruit of ‘Orah’. We identified 90d and 150 DAF as the best time for fruit thinning, which can be used for the extraction of functional substances. Additionally, We identified ‘*X’* as the most commercially viable option, ‘*ZC’* in terms of functionality, ‘*H’* in terms of antioxidant ability. These findings can be used to determine the optimal rootstock and fruit thinning for ‘Orah’, based on the expected effect.

## Data Availability

The original contributions presented in the study are included in the article/**Supplementary Material**. Further inquiries can be directed to the corresponding author.

## Supplementary material

Supplementary Figure 1 Changes in the content of three kinds of phenolic acid in ‘Orah’ under grafting of different rootstocks-scion combinations. HP: Peel of ‘Orah’ grafted on C. reticulata Blanco cv. Red tangerine, HR: Pulp of ‘Orah’ grafted on C. reticulata Blanco cv. Red tangerine, XP: Peel of ‘Orah’ grafted on C. junos Sieb. ex Tanaka,XR: Pulp of ‘Orah’ grafted on C. junos Sieb. ex Tanaka, ZP: Peel of ‘Orah’ grafted on P. trifoliata (L.) Raf, ZR: Pulp of ‘Orah’ grafted on P. trifoliata (L.) Raf, ZCP: Peel of ‘Orah’ grafted on C. sinensis Osb.× P. trifoliate Raf, ZCR: Pulp of ‘Orah’ grafted on C. sinensis Osb.× P. trifoliate Raf. 90, 120, 150, 180, 210, 240 DAF: Fruit at 90d, 120d, 150d, 180d, 210d, 240d after flowering, FW: Fresh weight. FW: Fresh weight. Different lowercase letters in the same rootstock at different times represented significant difference (P<0.05), while capital letters represent significant differences in the different rootstocks at the same time period (P<0.05).

Supplementary Figure 2 Changes in the content of 7 flavonoids in ‘Orah’ under grafting of different rootstocks. HP: Peel of ‘Orah’ grafted on C. reticulata Blanco cv. Red tangerine, HR: Pulp of ‘Orah’ grafted on C. reticulata Blanco cv. Red tangerine, XP: Peel of ‘Orah’ grafted on C. junos Sieb. ex Tanaka,XR: Pulp of ‘Orah’ grafted on C. junos Sieb. ex Tanaka, ZP: Peel of ‘Orah’ grafted on P. trifoliata (L.) Raf, ZR: Pulp of ‘Orah’ grafted on P. trifoliata (L.) Raf, ZCP: Peel of ‘Orah’ grafted on C. sinensis Osb.× P. trifoliate Raf, ZCR: Pulp of ‘Orah’ grafted on C. sinensis Osb.× P. trifoliate Raf. 90, 120, 150, 180, 210, 240 DAF: Fruit at 90d, 120d, 150d, 180d, 210d, 240d after flowering, FW: Fresh weight. FW: Fresh weight. Different lowercase letters in the same rootstock at different times represented significant difference (P<0.05), while capital letters represent significant differences in the different rootstocks at the same time period (P<0.05).

## Appendix A

H Red orange *Citrus reticulata* Blanco cv. Red tangerine

X Ziyang xiangcheng (*Citrus junos* Sieb. ex Tanaka)

Z Trifoliate orange (*Poncirus trifoliata* L. Raf)

ZC Carrizo citrange (*Citrus.sinensis* Osb.×*P.trifoliate* Raf)

HP The peel of ‘Orah’ grafted on Red orange *Citrus reticulata* Blanco cv. Red tangerine

HR The pulp of ‘Orah’ grafted on Red orange *Citrus reticulata* Blanco cv. Red tangerine

XP The peel of ‘Orah’ grafted on Ziyang xiangcheng *Citrus junos* Sieb. ex Tanaka

XR The pulp of ‘Orah’ grafted on Ziyang xiangcheng *Citrus junos* Sieb. ex Tanaka

ZP The peel of ‘Orah’ grafted on Trifoliate orange *Poncirus trifoliata* L. Raf

ZR The pulp of ‘Orah’ grafted on Trifoliate orange *Poncirus trifoliata* L. Raf

ZCP The peel of ‘Orah’ grafted on Carrizo citrange *Citrus.sinensis* Osb.×*P.trifoliate* Raf

ZCR The pulp of ‘Orah’ grafted on Carrizo citrange *Citrus.sinensis* Osb.×*P.trifoliate* Raf

90d Fruit at 90d after flowering

120d Fruit at 120d after flowering

150d Fruit at 150d after flowering.

180d Fruit at 180d after flowering

210d Fruit at 210d after flowering

240d Fruit at 240d after flowering

VC Vitamin C

RTT ratio of TSS to TA
