# Supplementary figures and images for: Effects of rootstocks and developmental time on the dynamic changes of main functional substances in ‘Orah’ (Citrus reticulata Blanco) by HPLC coupled with UV detection
Source: Front Plant Sci. 2024 Aug 27;15:1382768. doi: 10.3389/fpls.2024.1382768 (PMC11388320; doi:10.3389/fpls.2024.1382768)

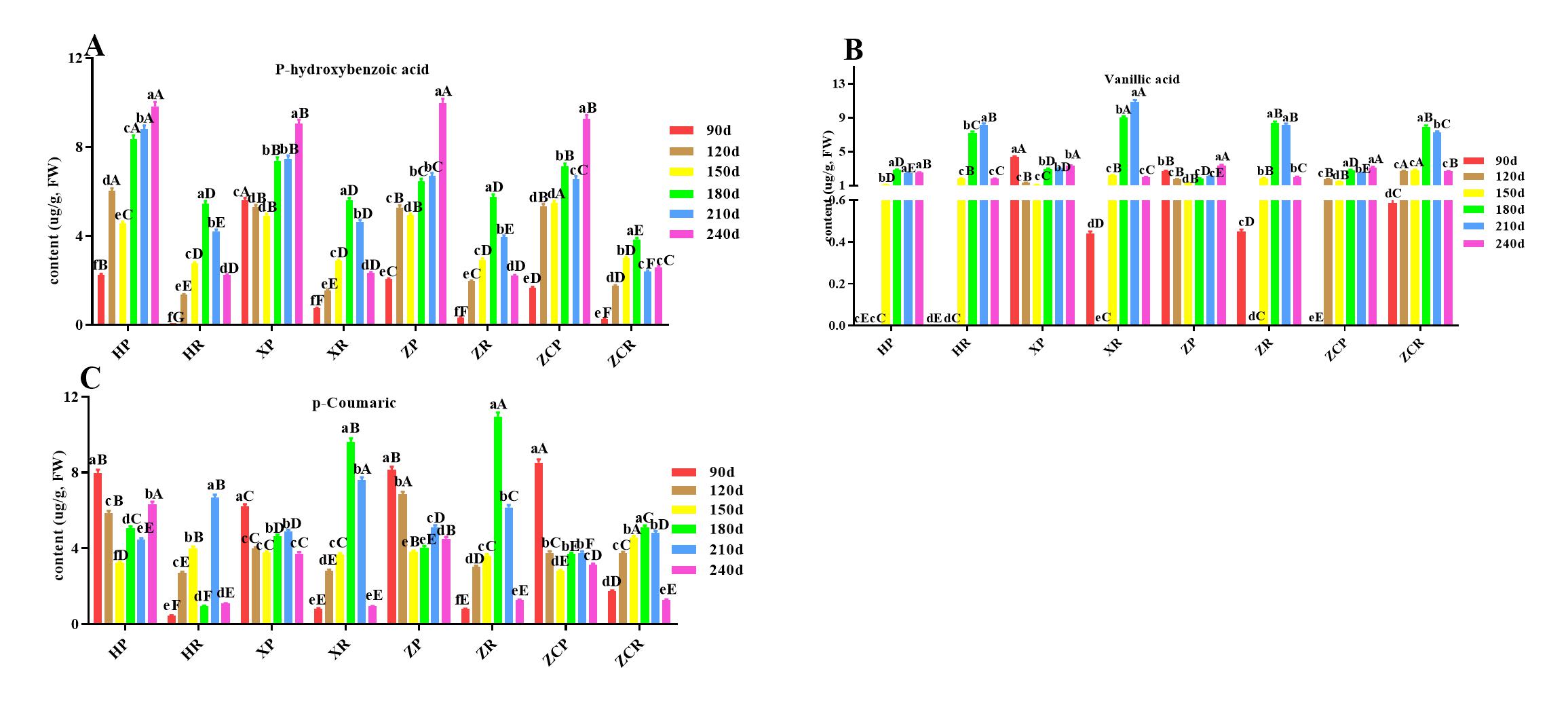

Supplement: Supplementary Figure 1 — Changes in the content of three kinds of phenolic acid in ‘Orah’ under grafting of different rootstocks-scion combinations. HP: Peel of ‘Orah’ grafted on C. reticulata Blanco cv. Red tangerine, HR: Pulp of ‘Orah’ grafted on C. reticulata Blanco cv. Red tangerine, XP: Peel of ‘Orah’ grafted on C. junos Sieb. ex Tanaka,XR: Pulp of ‘Orah’ grafted on C. junos Sieb. ex Tanaka, ZP: Peel of ‘Orah’ grafted on P. trifoliata (L.) Raf, ZR: Pulp of ‘Orah’ grafted on P. trifoliata (L.) Raf, ZCP: Peel of ‘Orah’ grafted on C. sinensis Osb.× P. trifoliate Raf, ZCR: Pulp of ‘Orah’ grafted on C. sinensis Osb.× P. trifoliate Raf. 90, 120, 150, 180, 210, 240 DAF: Fruit at 90d, 120d, 150d, 180d, 210d, 240d after flowering, FW: Fresh weight. FW: Fresh weight. Different lowercase letters in the same rootstock at different times represented significant difference (P<0.05), while capital letters represent significant differences in the different rootstocks at the same time period (P<0.05). [file Image1.jpeg]

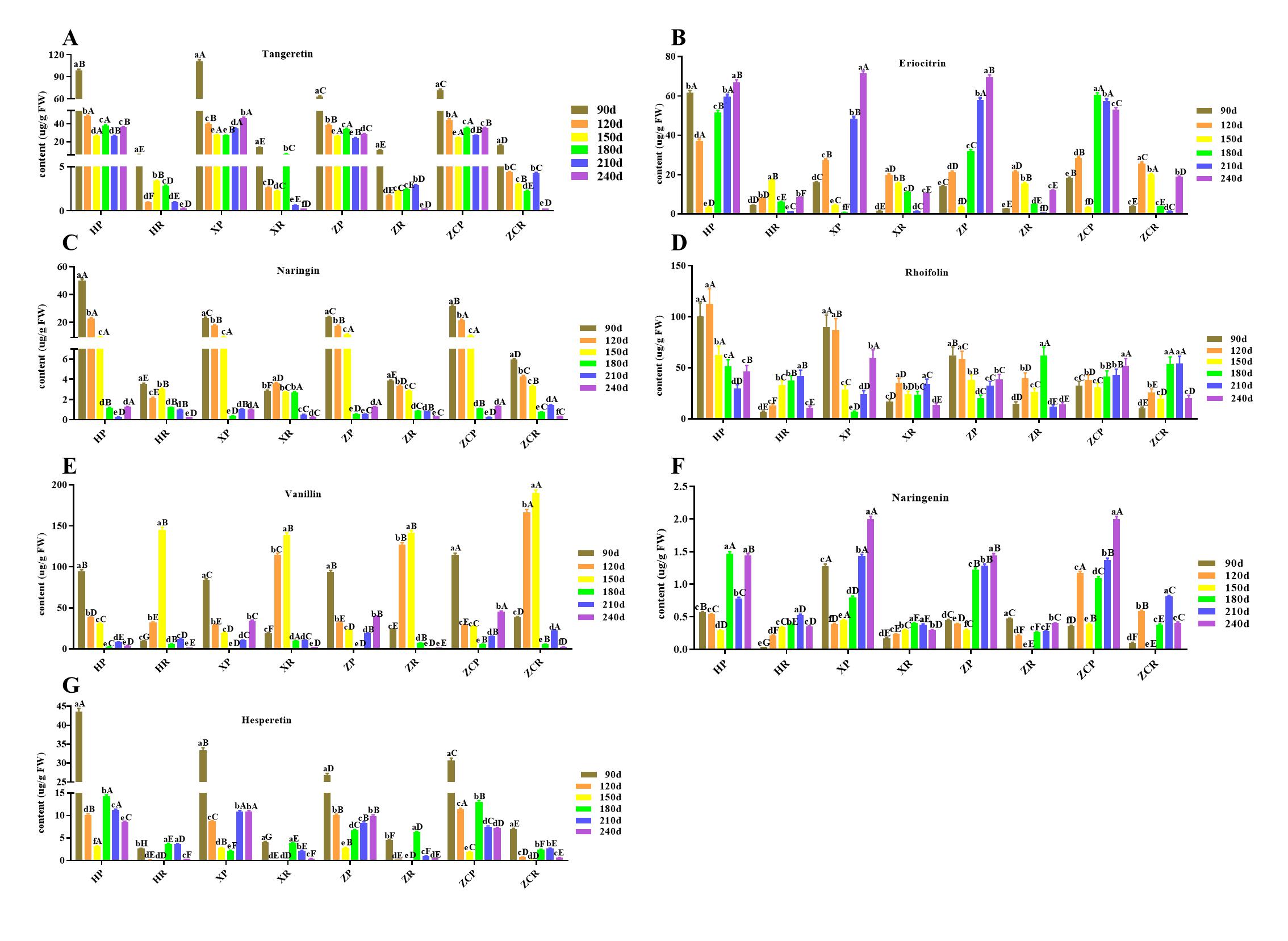

Supplement: Supplementary Figure 2 — Changes in the content of 7 flavonoids in ‘Orah’ under grafting of different rootstocks. HP: Peel of ‘Orah’ grafted on C. reticulata Blanco cv. Red tangerine, HR: Pulp of ‘Orah’ grafted on C. reticulata Blanco cv. Red tangerine, XP: Peel of ‘Orah’ grafted on C. junos Sieb. ex Tanaka,XR: Pulp of ‘Orah’ grafted on C. junos Sieb. ex Tanaka, ZP: Peel of ‘Orah’ grafted on P. trifoliata (L.) Raf, ZR: Pulp of ‘Orah’ grafted on P. trifoliata (L.) Raf, ZCP: Peel of ‘Orah’ grafted on C. sinensis Osb.× P. trifoliate Raf, ZCR: Pulp of ‘Orah’ grafted on C. sinensis Osb.× P. trifoliate Raf. 90, 120, 150, 180, 210, 240 DAF: Fruit at 90d, 120d, 150d, 180d, 210d, 240d after flowering, FW: Fresh weight. FW: Fresh weight. Different lowercase letters in the same rootstock at different times represented significant difference (P<0.05), while capital letters represent significant differences in the different rootstocks at the same time period (P<0.05). [file Image2.jpeg]
