# Supplementary material for: Effects of rootstocks and developmental time on the dynamic changes of main functional substances in ‘Orah’ (Citrus reticulata Blanco) by HPLC coupled with UV detection
Source: Front Plant Sci. 2024 Aug 27;15:1382768. doi: 10.3389/fpls.2024.1382768 (PMC11388320; doi:10.3389/fpls.2024.1382768)
Supplement: Supplementary file 3 [file Table1.docx]

Supplementary Table 1 Correlation analysis among the functional substances in fruits of ‘Orah’ grafted to the four rootstocks

|  | FRAP | DPPH | ABTS | Linimon | Nomilin | Synephrine | Protocatechuic | Sinapic | Ferulic | caffeic | Sinensetin | Nobiletin | Narirutin | Hesperidin | Neohesperiklin | Poncirin |
| --- | --- | --- | --- | --- | --- | --- | --- | --- | --- | --- | --- | --- | --- | --- | --- | --- |
| FRAP | 1 |  |  |  |  |  |  |  |  |  |  |  |  |  |  |  |
| DPPH | .897** | 1 |  |  |  |  |  |  |  |  |  |  |  |  |  |  |
| ABTS | .725** | .830** | 1 |  |  |  |  |  |  |  |  |  |  |  |  |  |
| Linimon | .569** | .543** | .481** | 1 |  |  |  |  |  |  |  |  |  |  |  |  |
| Nomilin | 0.204106 | 0.256276 | .334* | .689** | 1 |  |  |  |  |  |  |  |  |  |  |  |
| Synephrine | .854** | .898** | .813** | .541** | 0.241184 | 1 |  |  |  |  |  |  |  |  |  |  |
| Protocatechuic | 0.216108 | .360* | .290* | 0.190833 | 0.107136 | .438** | 1 |  |  |  |  |  |  |  |  |  |
| Sinapic | -0.03135 | -0.03507 | -0.04124 | -0.143 | -0.00392 | -0.11365 | .277** | 1 |  |  |  |  |  |  |  |  |
| Ferulic | -0.0401 | 0.121586 | 0.125718 | -0.095 | 0.00049 | 0.104791 | .779** | .597** | 1 |  |  |  |  |  |  |  |
| caffeic | -.500** | -.378** | -0.06837 | -.431** | -0.02652 | -.491** | -0.25809 | 0.111453 | 0.17852 | 1 |  |  |  |  |  |  |
| Sinensetin | 0.179448 | .333* | .323* | 0.083188 | 0.063761 | .410** | .861** | 0.050555 | .628** | -0.11691 | 1 |  |  |  |  |  |
| Nobiletin | 0.2209 | .385** | .337* | 0.155375 | 0.059585 | .468** | .877** | 0.084176 | .601** | -0.21221 | .963** | 1 |  |  |  |  |
| Narirutin | -0.04478 | -0.13725 | -0.0913 | -0.19605 | -0.06066 | -0.18416 | 0.143579 | .901** | .542** | 0.072843 | -0.08143 | -0.08572 | 1 |  |  |  |
| Hesperidin | 0.046331 | 0.132393 | 0.141015 | -0.03748 | 0.061258 | 0.150136 | -.199* | -0.16158 | -.312** | 0.002864 | -.263** | -.252** | -.170* | 1 |  |  |
| Neohesperiklin | 0.175607 | .308* | .315* | -0.06133 | 0.052869 | .339* | -.382** | -.338** | -.487** | 0.029084 | -0.01017 | 0.044324 | -.415** | 0.14782 | 1 |  |
| Poncirin | .296* | .435** | .336* | 0.267251 | 0.114768 | .536** | -.168* | -.374** | -.469** | -.397** | 0.006235 | 0.068666 | -.483** | .719** | .704** | 1 |

*. The correlation is significant at level 0.05 (double tailed)

**. The correlation is significant at level 0.01 (double tailed)

Supplementary Table 2 Scores of principal components and membership function of functional substances in citrus reticulata under four rootstock ear combinations

| Species | Principal component score | Principal component ranking | Subordination score | Subordination ranking |
| --- | --- | --- | --- | --- |
| 90d-HP | 5.597585063 | 1 | 0.757963661 | 1 |
| 90d-HR | -1.736160439 | 48 | 0.107054127 | 48 |
| 90d-XP | 4.447995293 | 2 | 0.643139906 | 2 |
| 90d-XR | -1.361437981 | 43 | 0.138747393 | 43 |
| 90d-ZP | 4.042040613 | 3 | 0.613524525 | 3 |
| 90d-ZR | -1.251296434 | 41 | 0.147059384 | 42 |
| 90d-ZCP | 3.549058224 | 4 | 0.569636704 | 4 |
| 90d-ZCR | -0.891550246 | 35 | 0.179501204 | 40 |
| 120d-HP | 1.894510823 | 5 | 0.426614613 | 5 |
| 120d-HR | -0.035050699 | 19 | 0.261318638 | 24 |
| 120d-XP | 0.766347137 | 8 | 0.319822619 | 11 |
| 120d-XR | -0.430028085 | 25 | 0.221353016 | 34 |
| 120d-ZP | 1.172510318 | 7 | 0.357476235 | 9 |
| 120d-ZR | 0.006317595 | 18 | 0.265816172 | 23 |
| 120d-ZCP | 1.316740642 | 6 | 0.388908252 | 6 |
| 120d-ZCR | 0.479634093 | 10 | 0.311215698 | 12 |
| 150d-HP | 0.215751396 | 13 | 0.275089723 | 18 |
| 150d-HR | 0.101797535 | 15 | 0.272909086 | 20 |
| 150d-XP | -0.426091525 | 24 | 0.21652511 | 36 |
| 150d-XR | -0.259355927 | 22 | 0.235866679 | 32 |
| 150d-ZP | 0.023276834 | 17 | 0.258866024 | 25 |
| 150d-ZR | 0.192880851 | 14 | 0.282591443 | 16 |
| 150d-ZCP | -0.093078403 | 21 | 0.2503228 | 29 |
| 150d-ZCR | 0.48804111 | 9 | 0.307090288 | 13 |
| 180d-HP | -0.054359853 | 20 | 0.30267607 | 14 |
| 180d-HR | -0.561645639 | 28 | 0.274543778 | 19 |
| 180d-XP | -0.94251814 | 37 | 0.185176055 | 39 |
| 180d-XR | -0.582625519 | 29 | 0.275364283 | 17 |
| 180d-ZP | -0.299439402 | 23 | 0.266273013 | 22 |
| 180d-ZR | -0.546362784 | 27 | 0.294449966 | 15 |
| 180d-ZCP | -0.600979831 | 30 | 0.257854125 | 26 |
| 180d-ZCR | -0.796495223 | 33 | 0.252106689 | 27 |
| 210d-HP | -0.64382381 | 31 | 0.251873398 | 28 |
| 210d-HR | -0.968067119 | 38 | 0.228659897 | 33 |
| 210d-XP | -0.6645995 | 32 | 0.248049534 | 31 |
| 210d-XR | -1.020641618 | 39 | 0.211136961 | 37 |
| 210d-ZP | -0.895946 | 36 | 0.219563545 | 35 |
| 210d-ZR | -1.322855156 | 42 | 0.158010036 | 41 |
| 210d-ZCP | -1.030218657 | 40 | 0.205861238 | 38 |
| 210d-ZCR | -0.840842326 | 34 | 0.248292006 | 30 |
| 240d-HP | 0.045767691 | 16 | 0.324559242 | 10 |
| 240d-HR | -1.687578279 | 47 | 0.117395809 | 47 |
| 240d-XP | 0.475265995 | 11 | 0.371638913 | 7 |
| 240d-XR | -1.679703624 | 46 | 0.120667979 | 46 |
| 240d-ZP | -0.445429634 | 26 | 0.26977796 | 21 |
| 240d-ZR | -1.602795014 | 45 | 0.12762209 | 45 |
| 240d-ZCP | 0.424018955 | 12 | 0.363599037 | 8 |
| 240d-ZCR | -1.568553261 | 44 | 0.136631849 | 44 |
