# Supplementary material for: Effects of rootstocks and developmental time on the dynamic changes of main functional substances in ‘Orah’ (Citrus reticulata Blanco) by HPLC coupled with UV detection
Source: Front Plant Sci. 2024 Aug 27;15:1382768. doi: 10.3389/fpls.2024.1382768 (PMC11388320; doi:10.3389/fpls.2024.1382768)
Supplement: Supplementary file 5 [file Table3.docx]

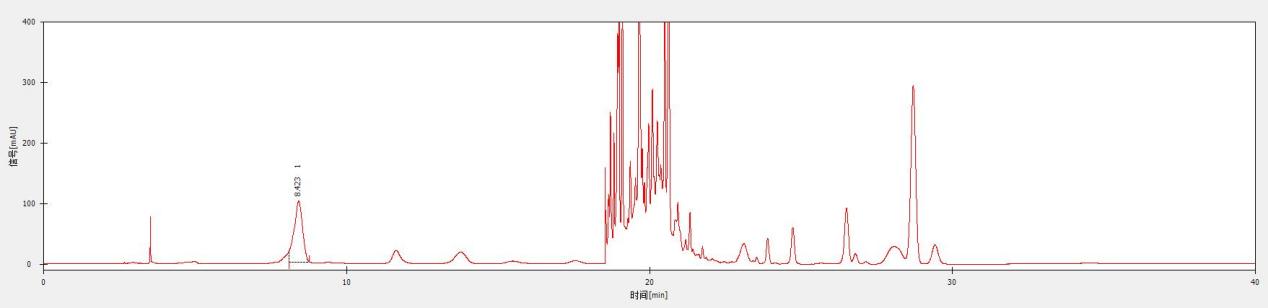


90d-HP


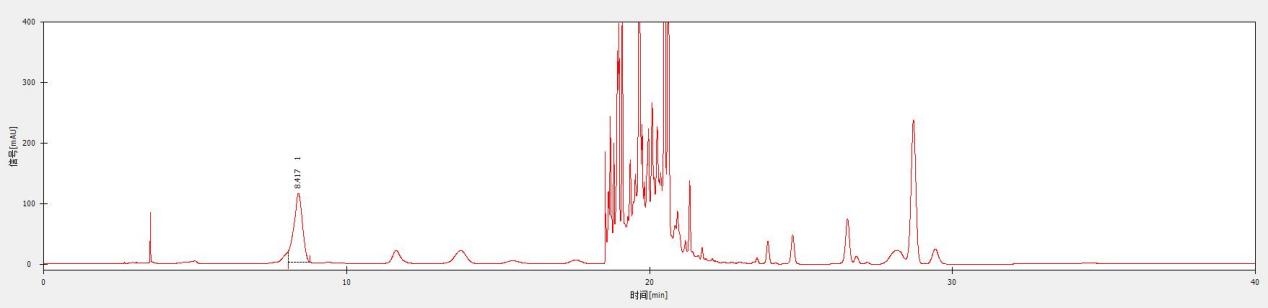


90d-HR


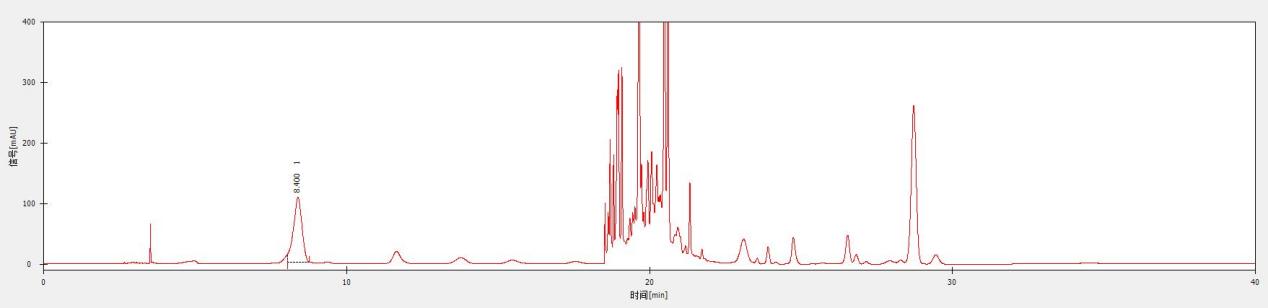

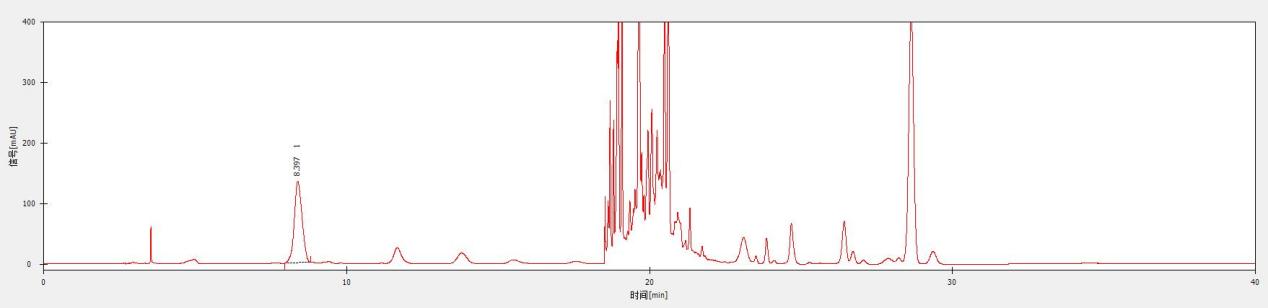


90d-XR

90d-XP


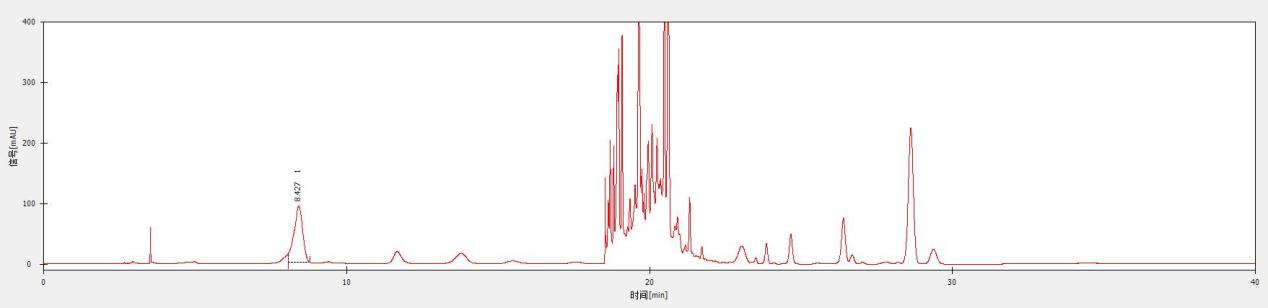


90d-ZP


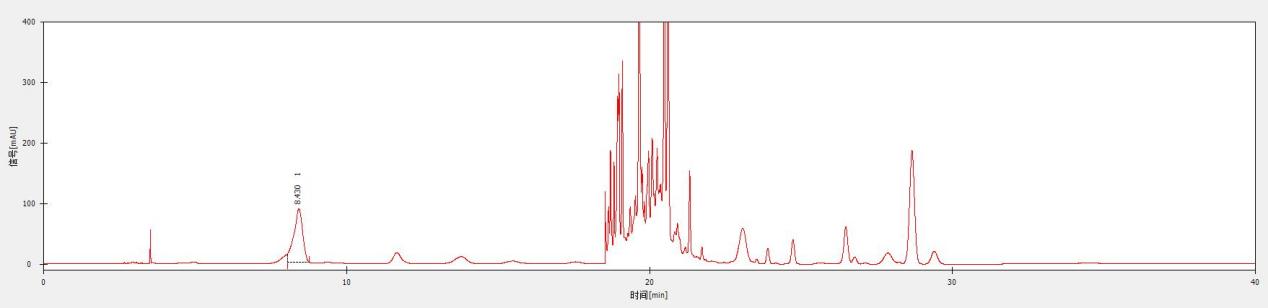


90d-ZR


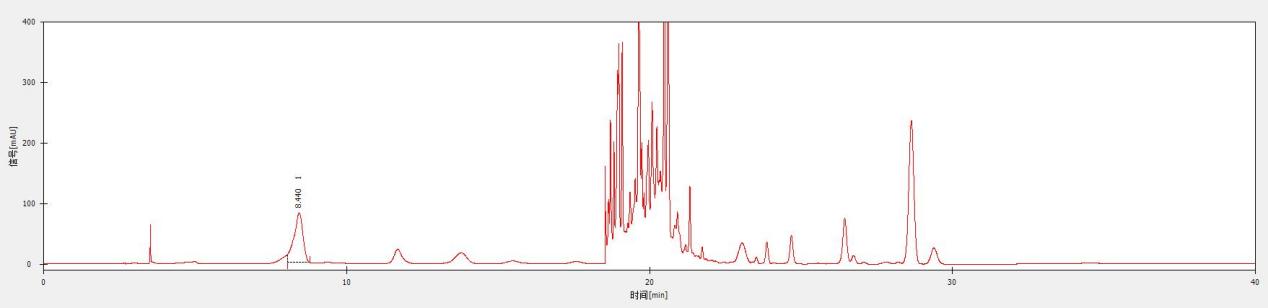


90d-ZCP


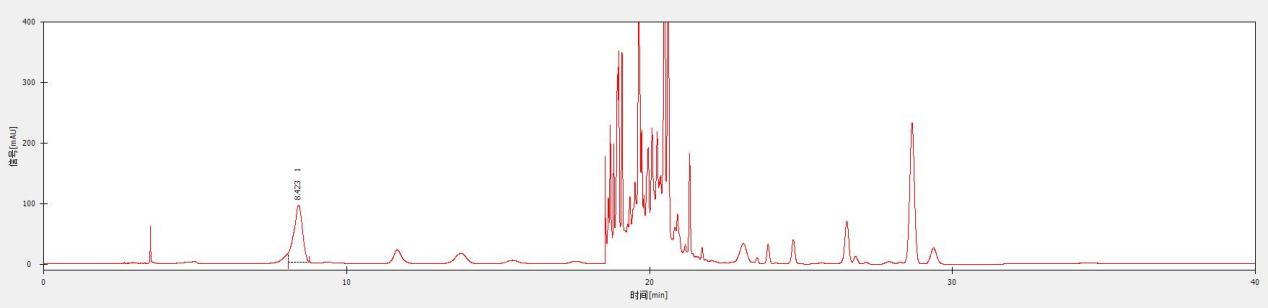


90d-ZCR


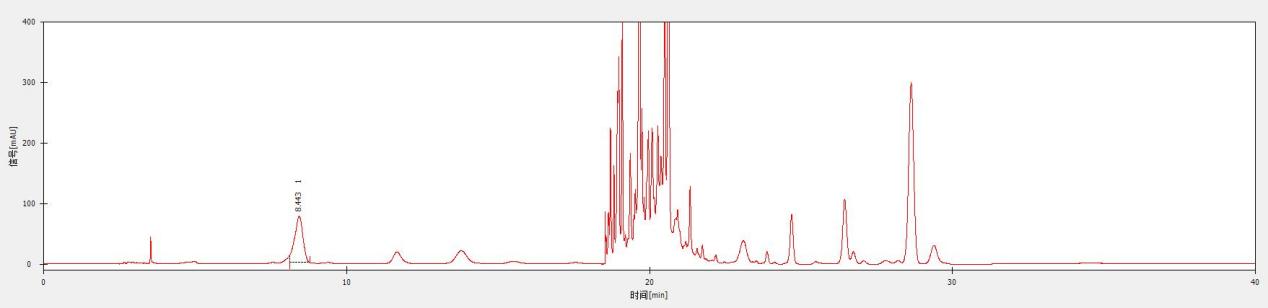


120d-HP


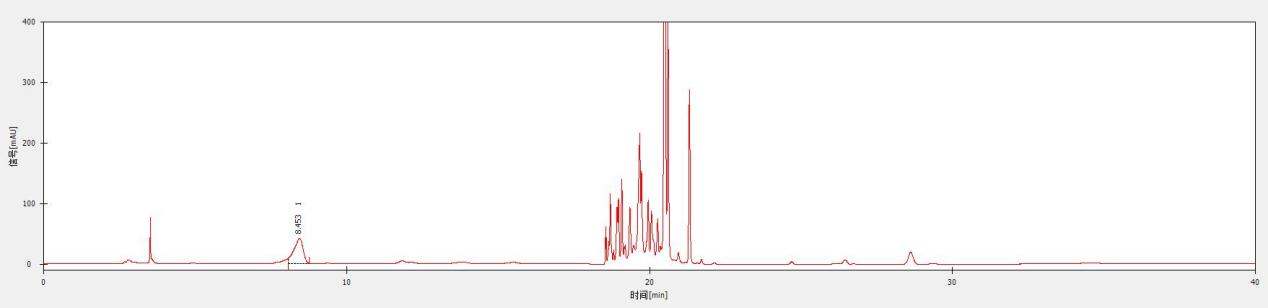


120d-HR


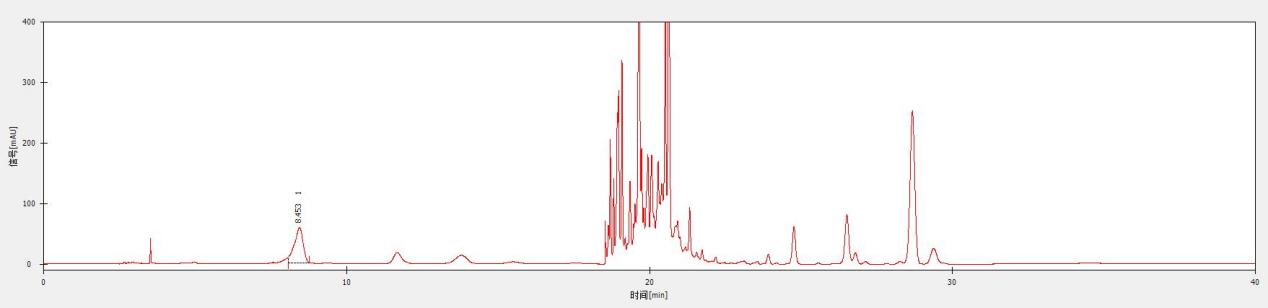


120d-XP


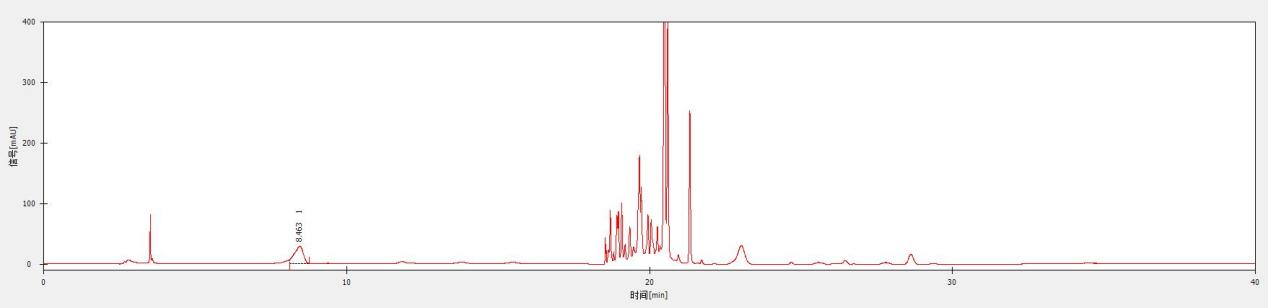


120d-XR


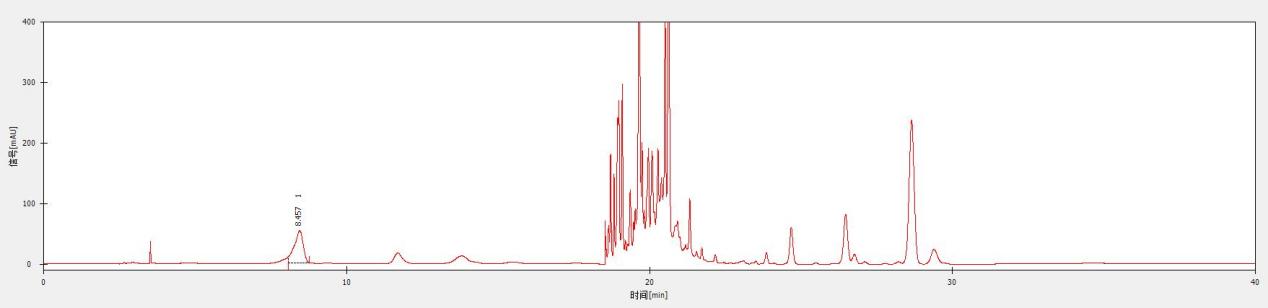


120d-ZP


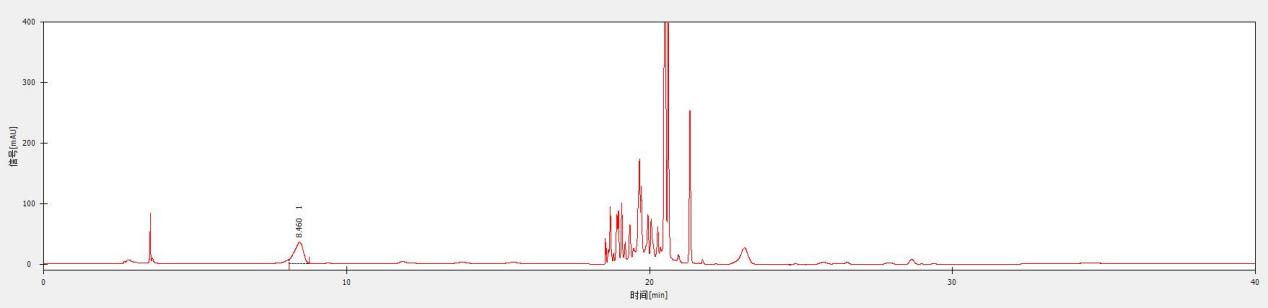


120d-ZR


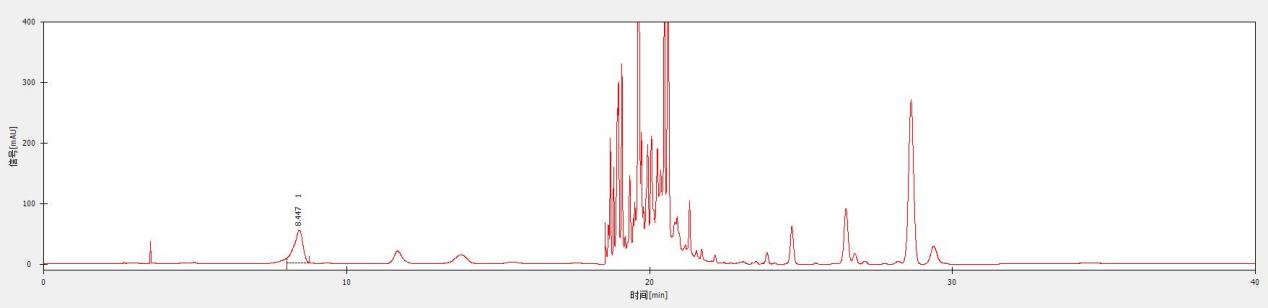


120d-ZCP


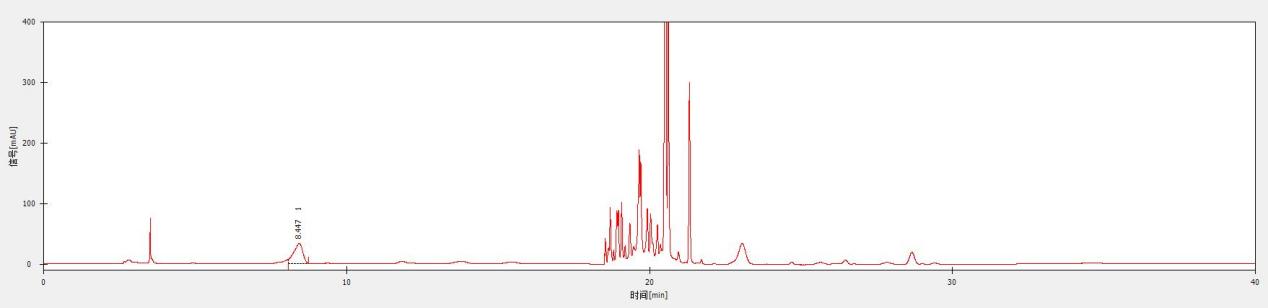


120d-ZCR


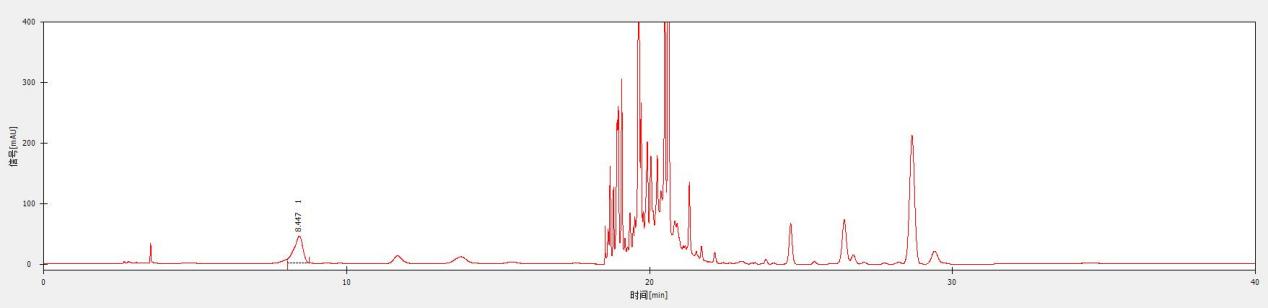


150d-HP


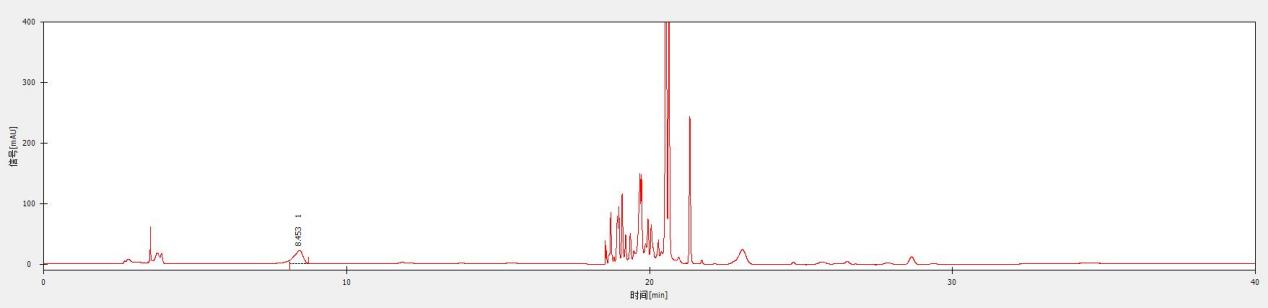


150d-HR


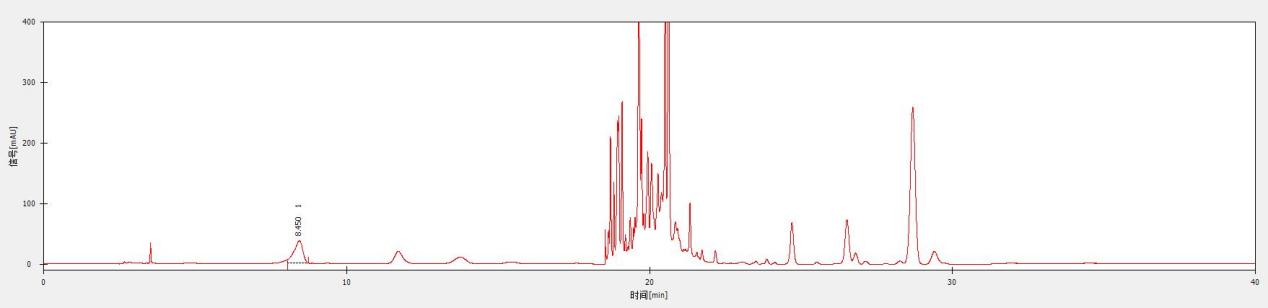


150d-XP


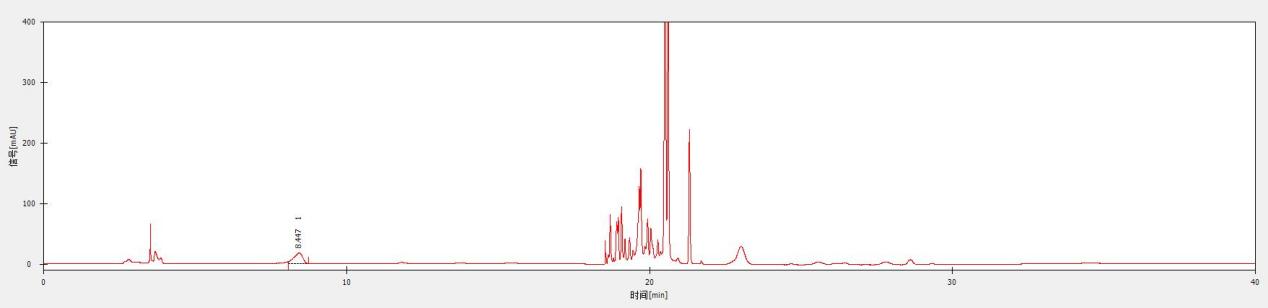


150d-XR


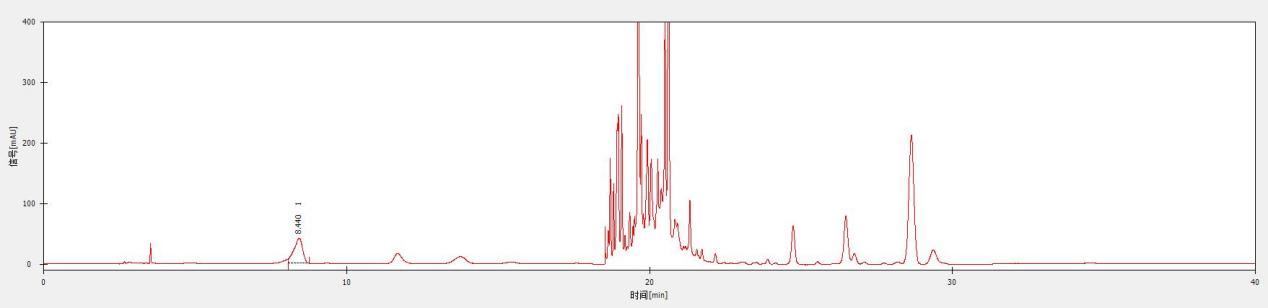


150d-ZP


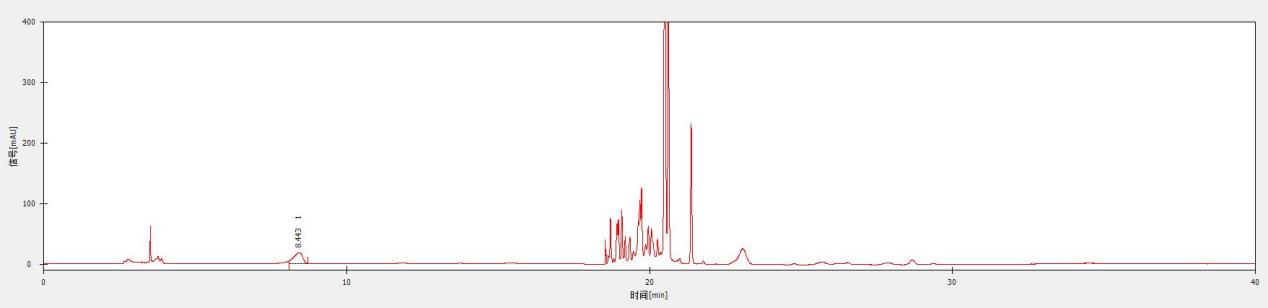


150d-ZR


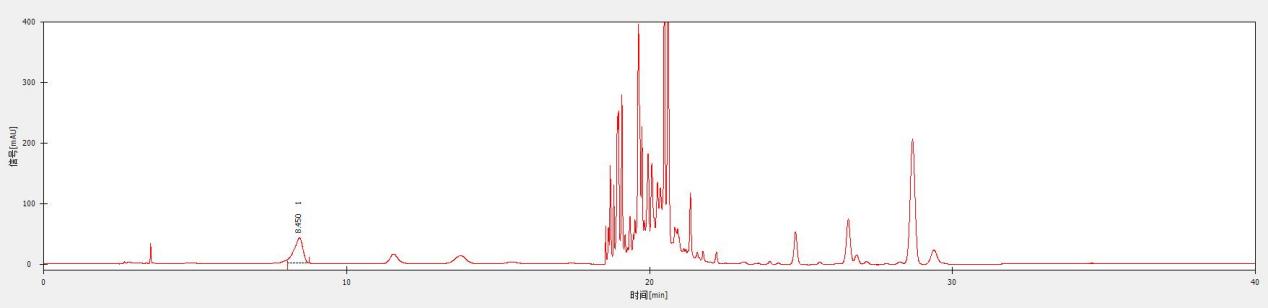


150d-ZCP


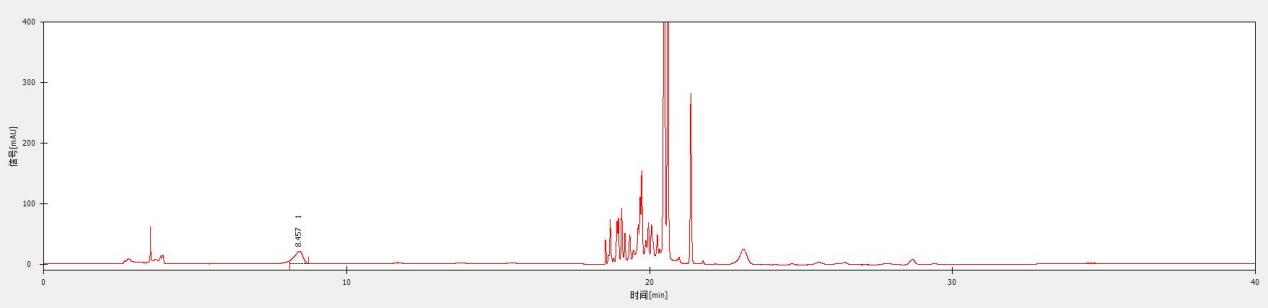


150d-ZCR


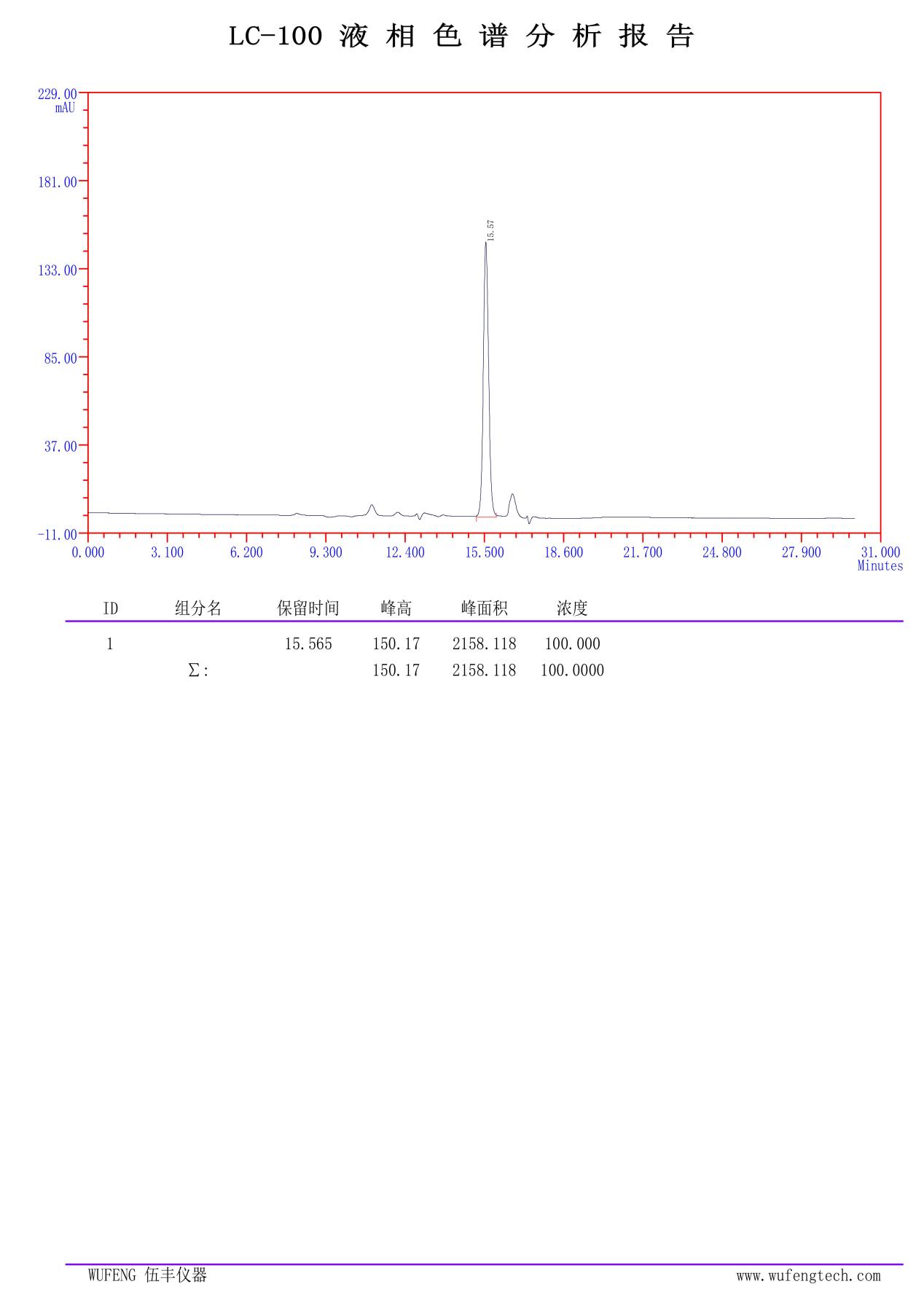


180d-HP


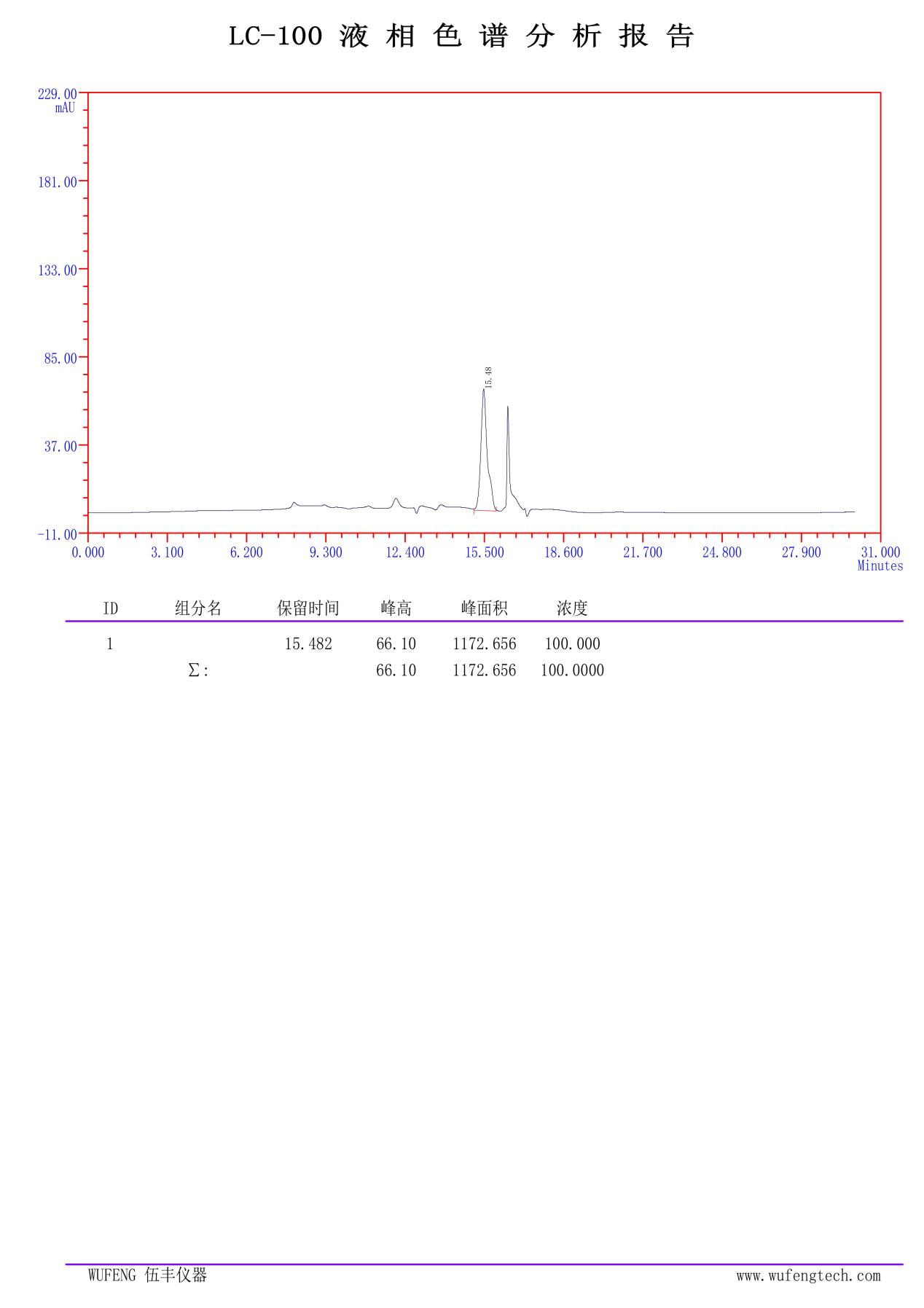


180d-HR


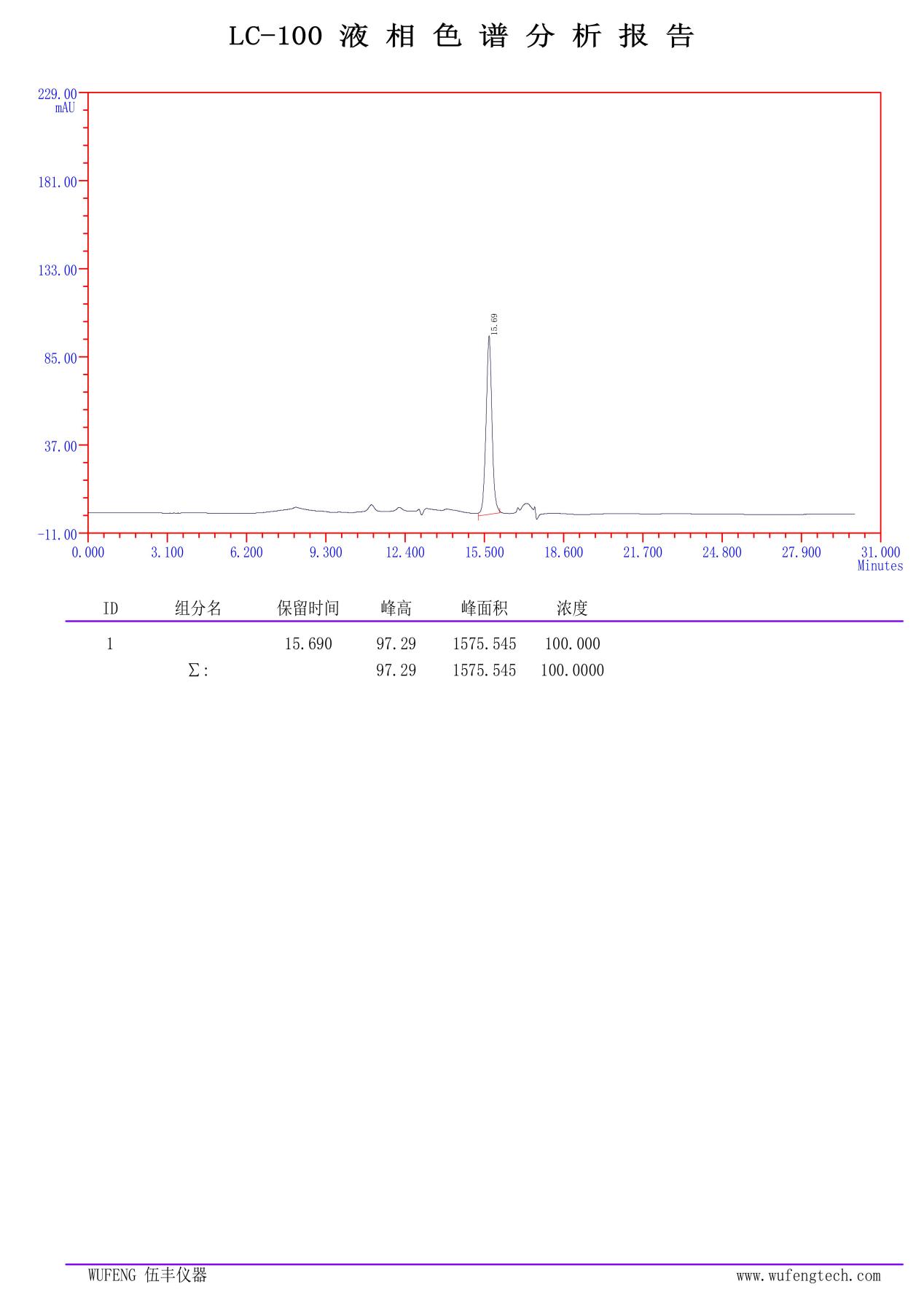


180d-XP


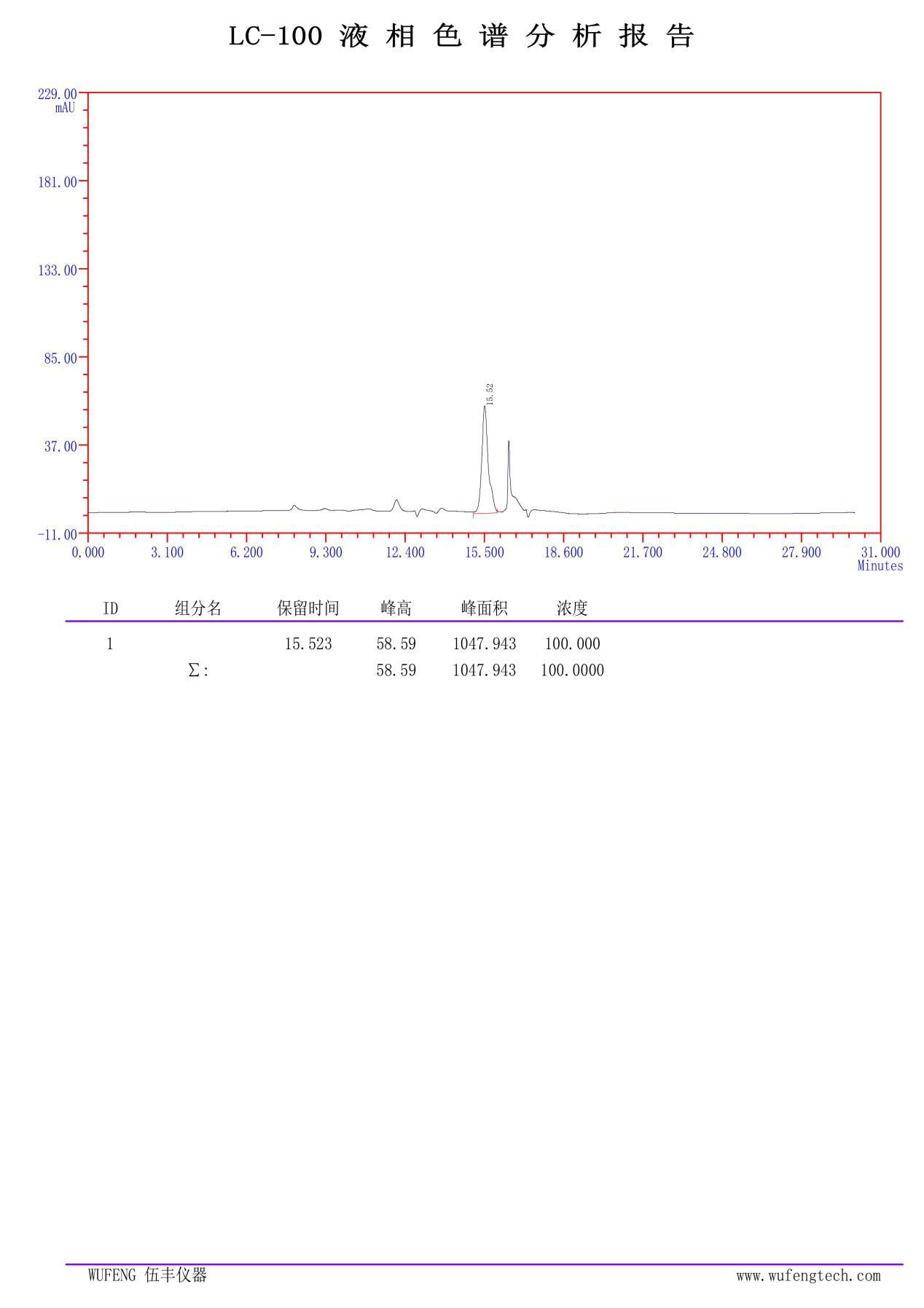


180d-XR


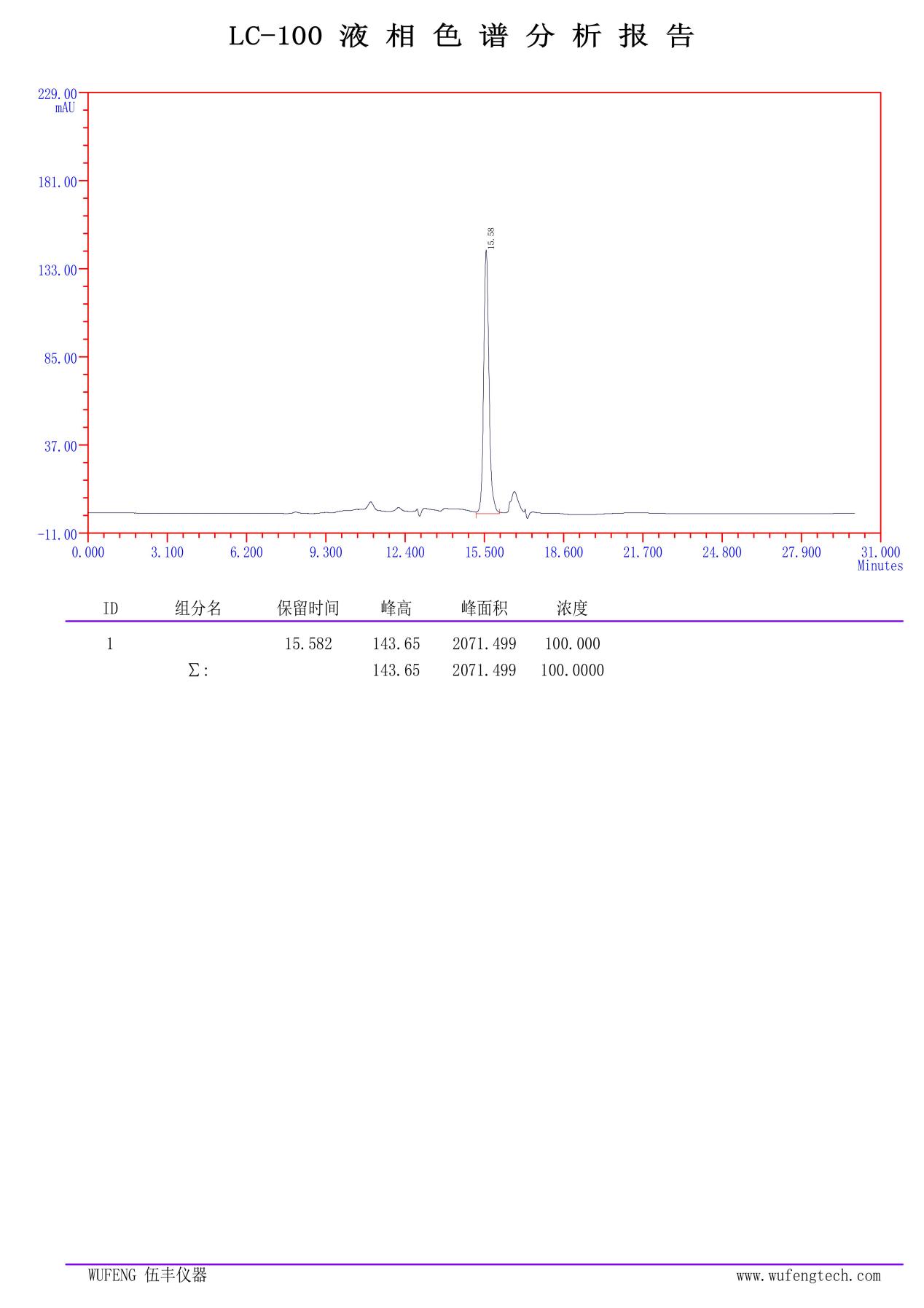


180d-ZP


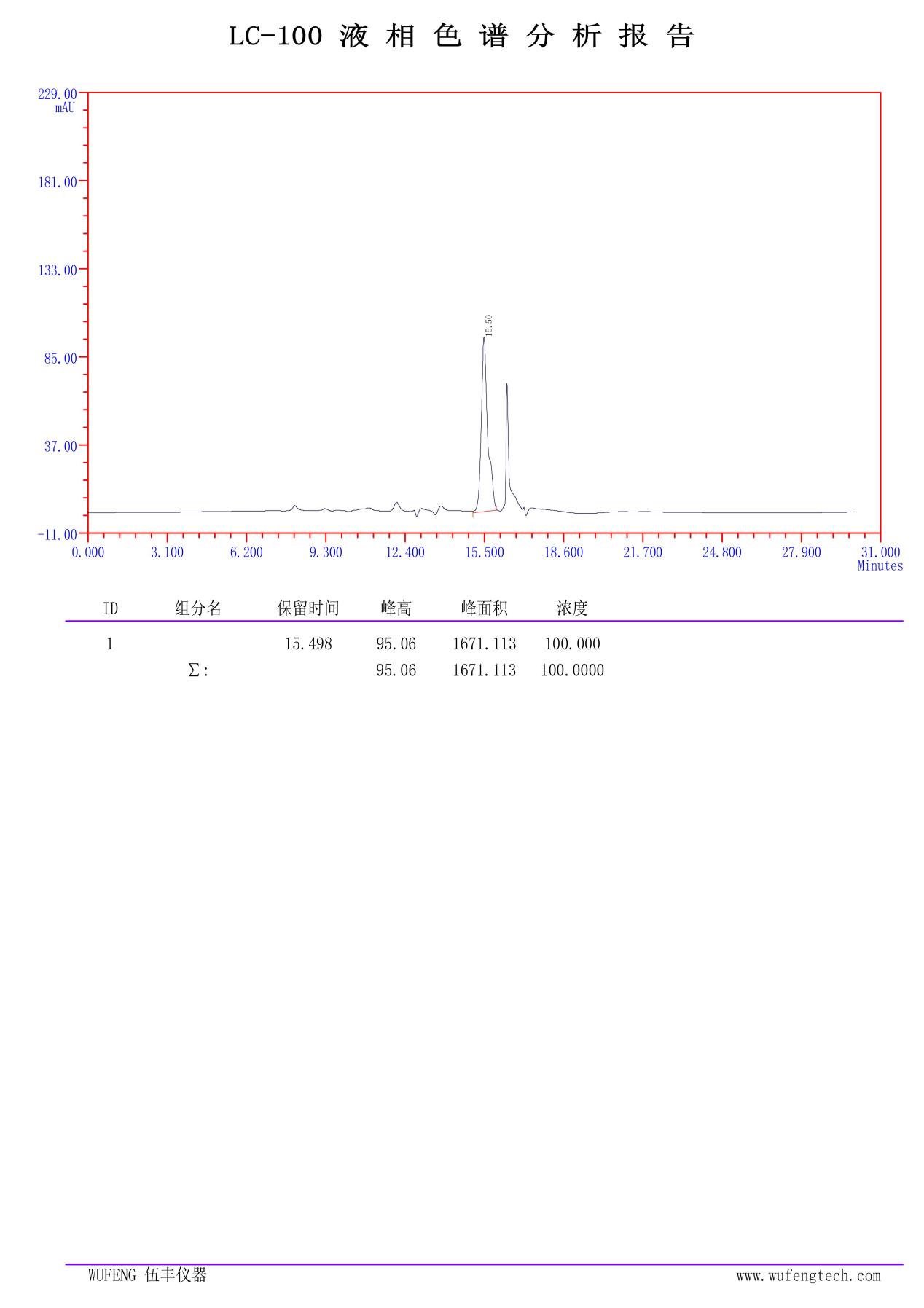


180d-ZR


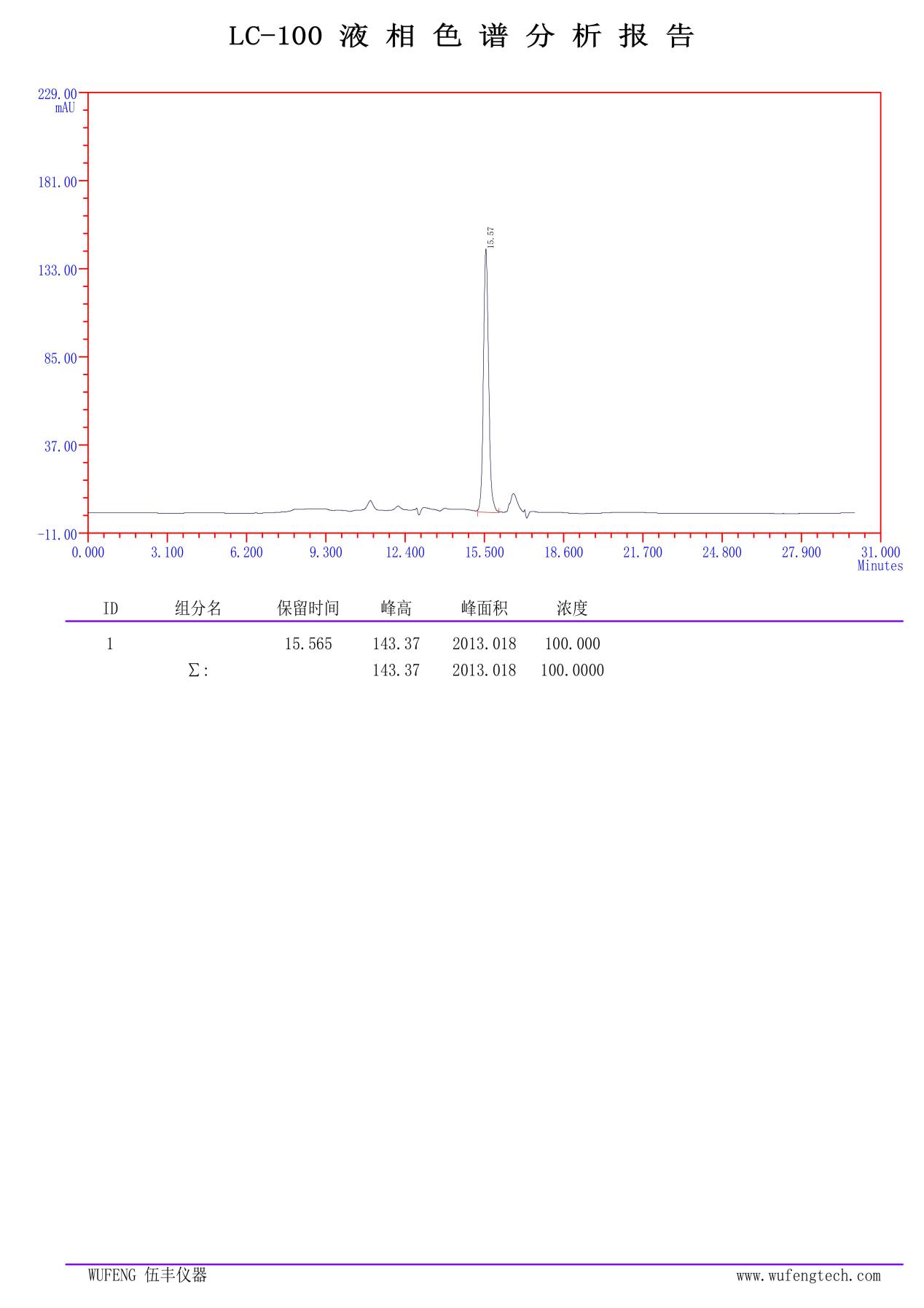


180d-ZCP


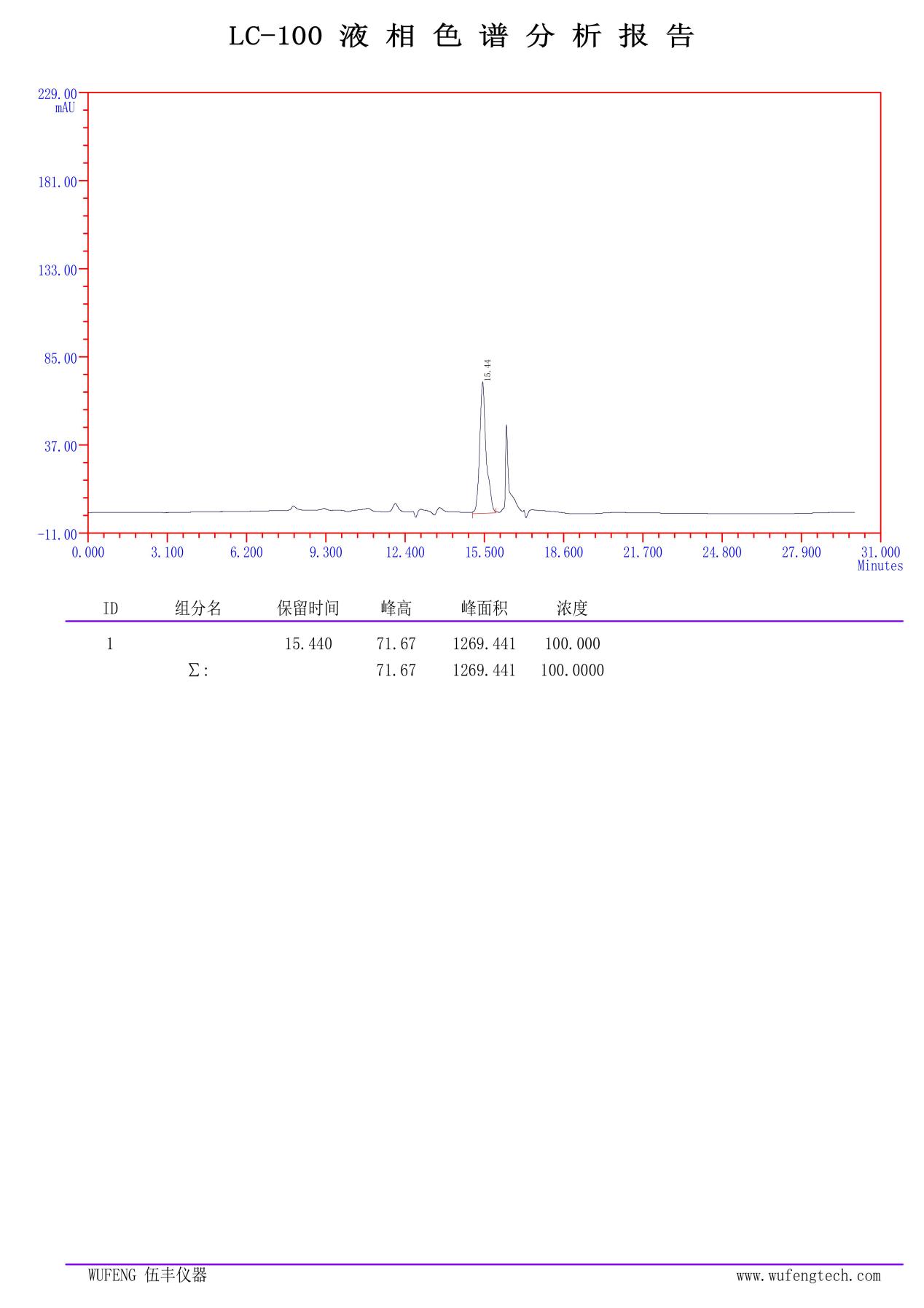


180d-ZCR


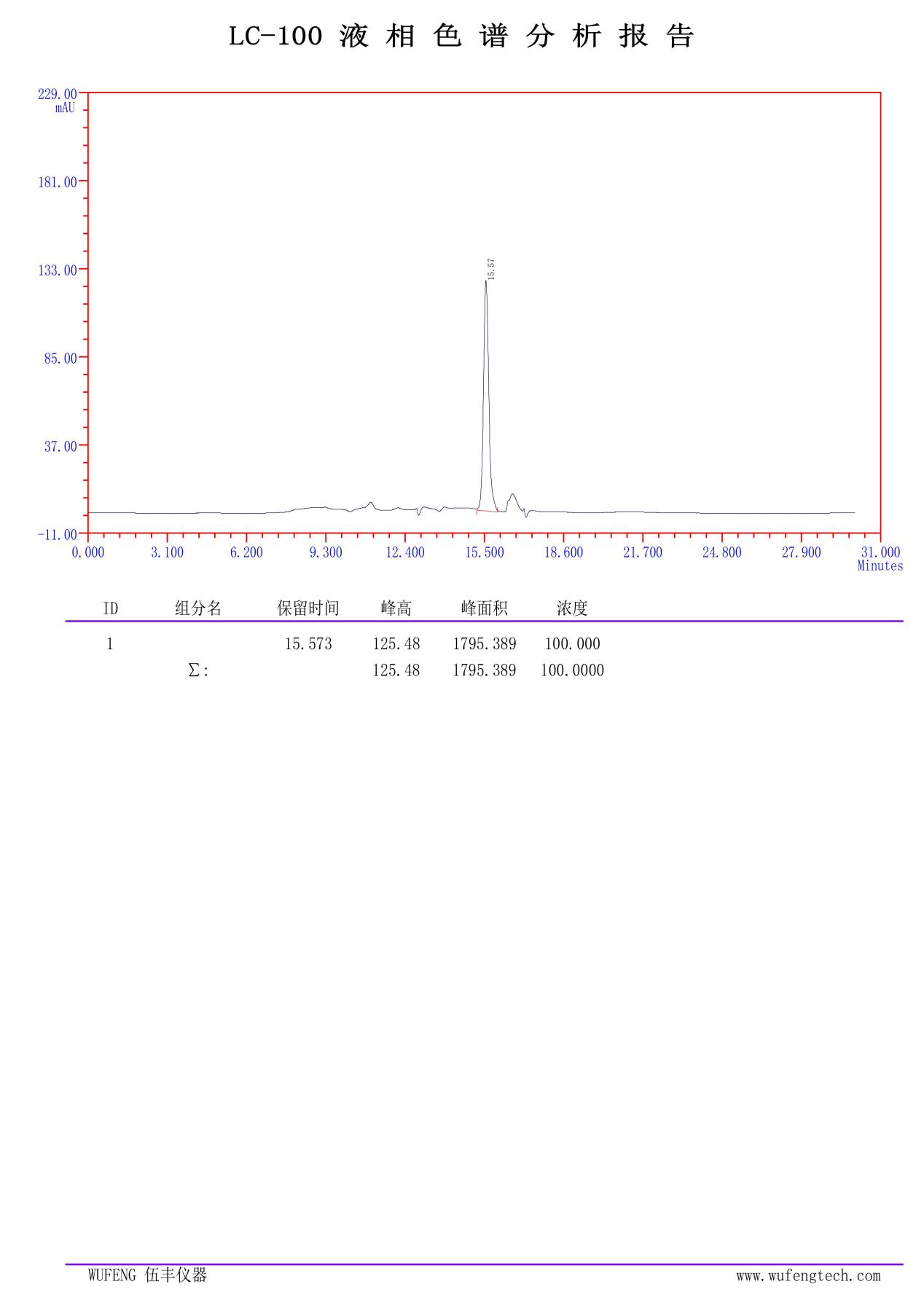


210d-HP


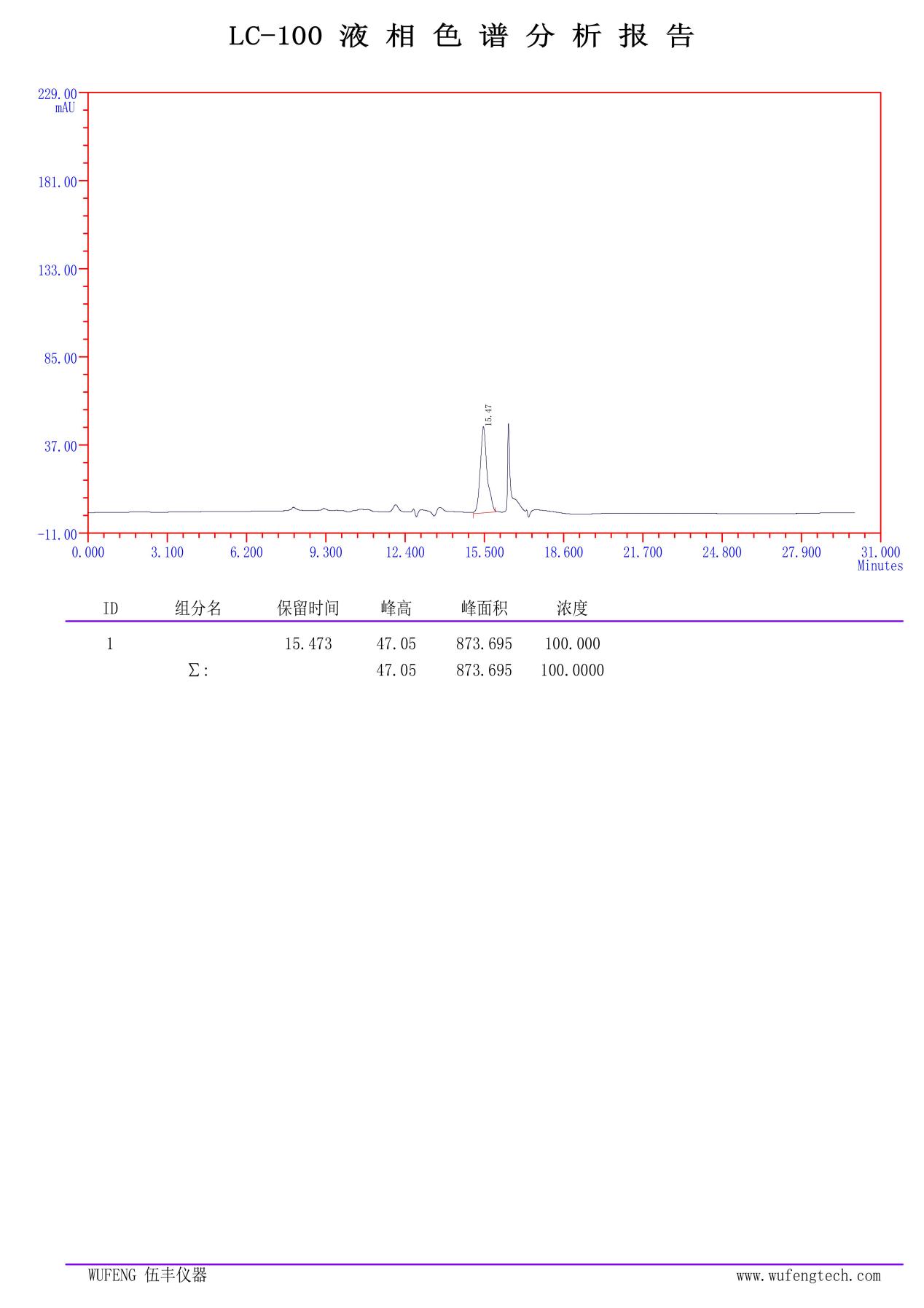


210d-HR


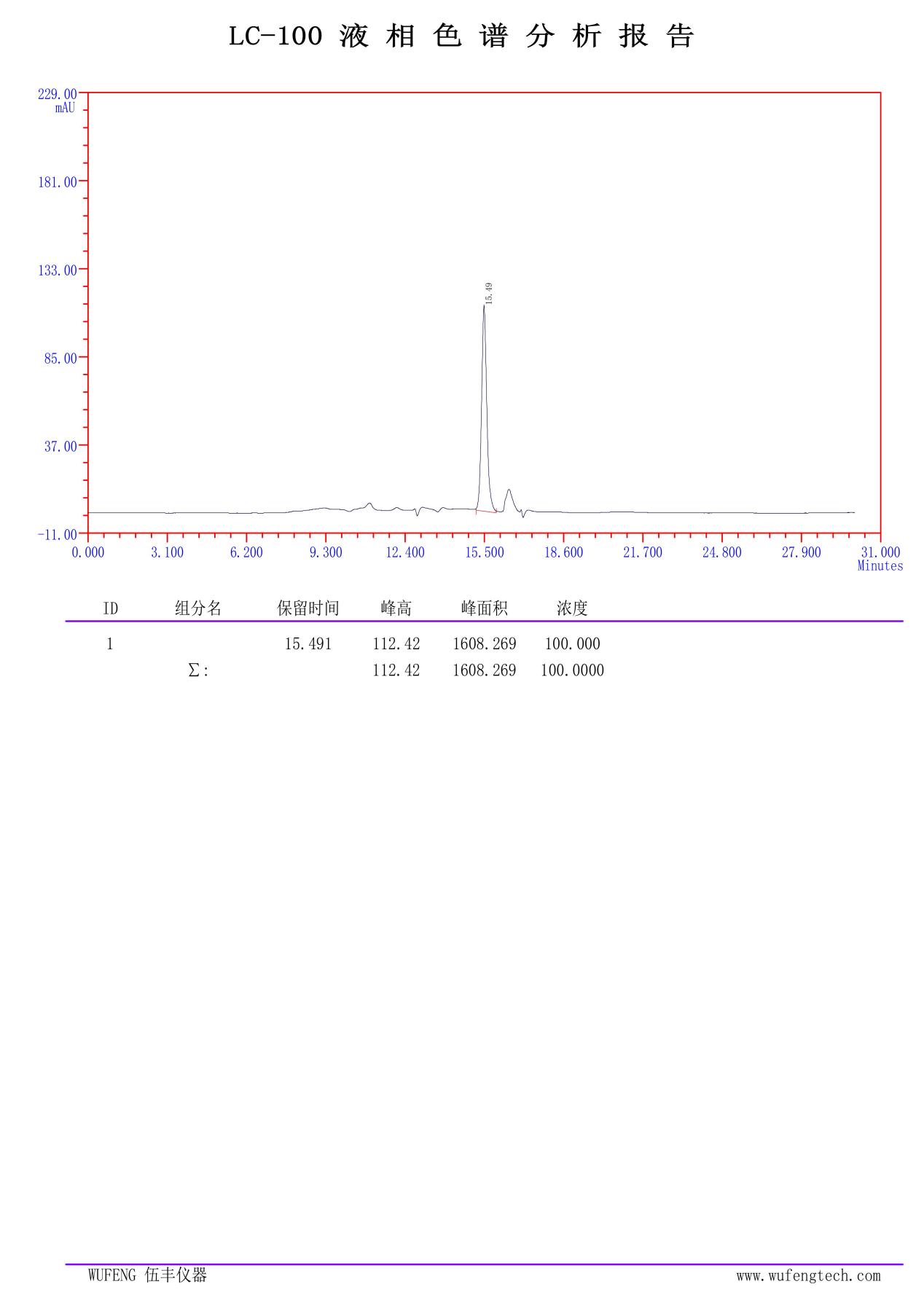


210d-XP


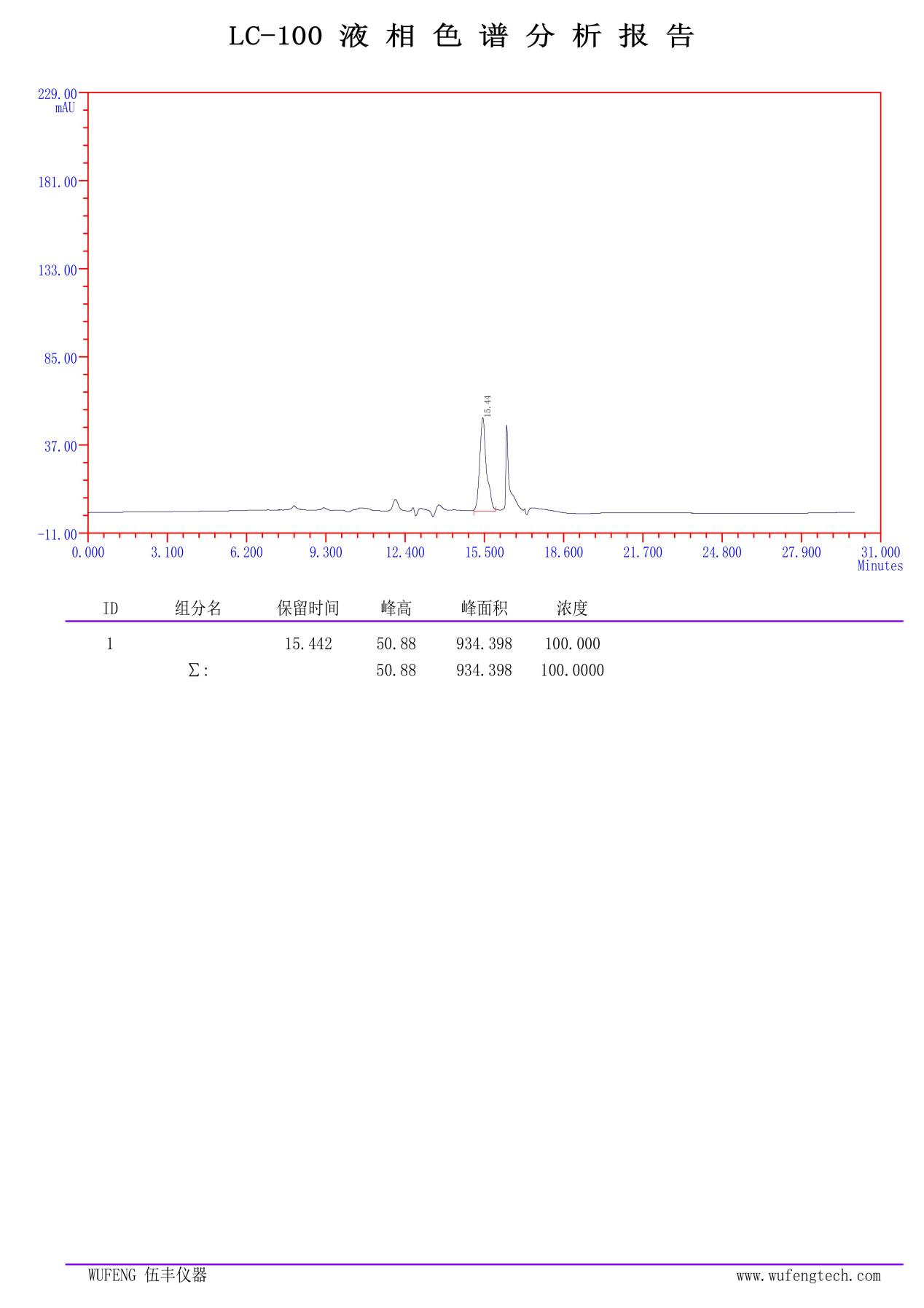


210d-XR


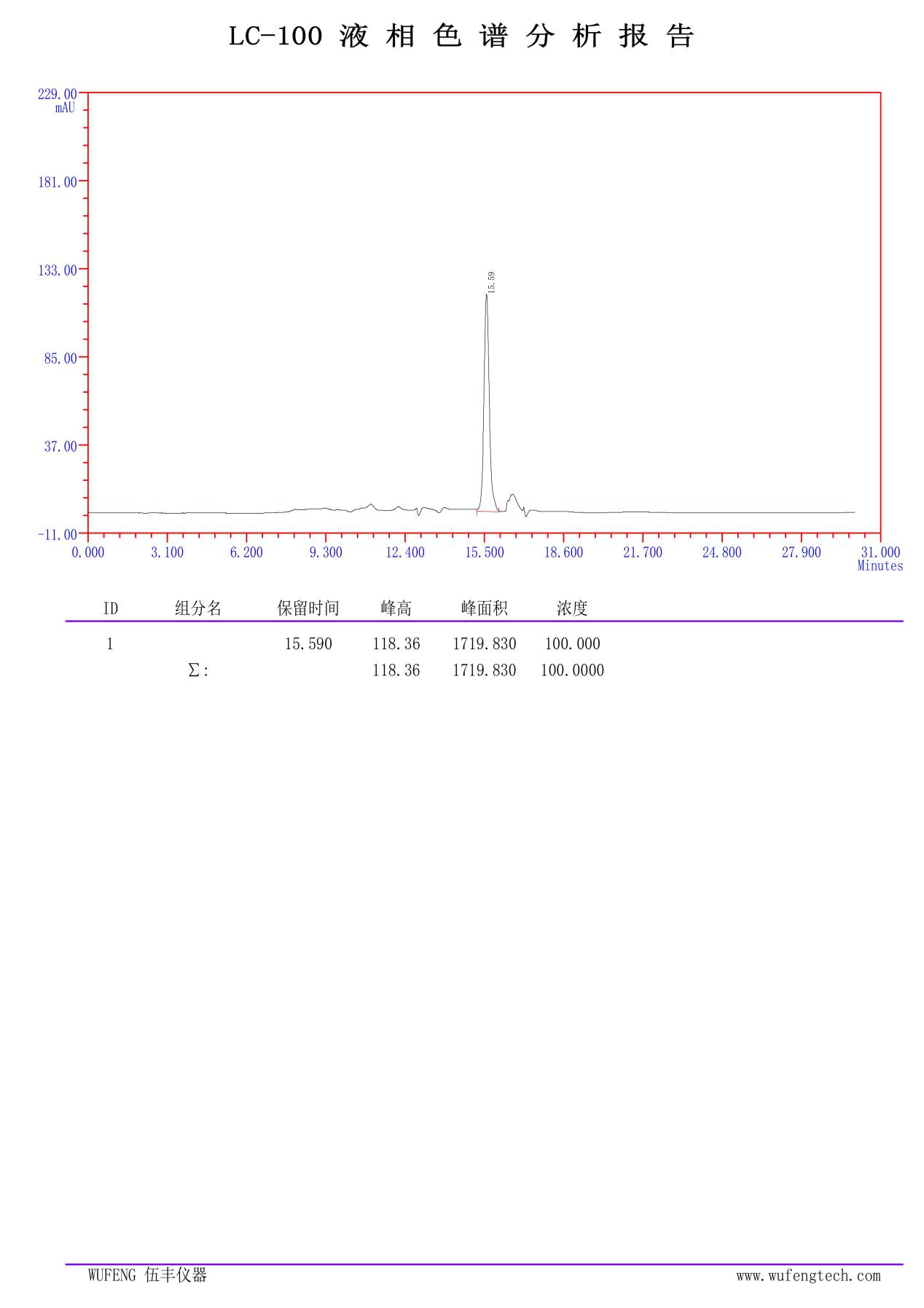


210d-ZP


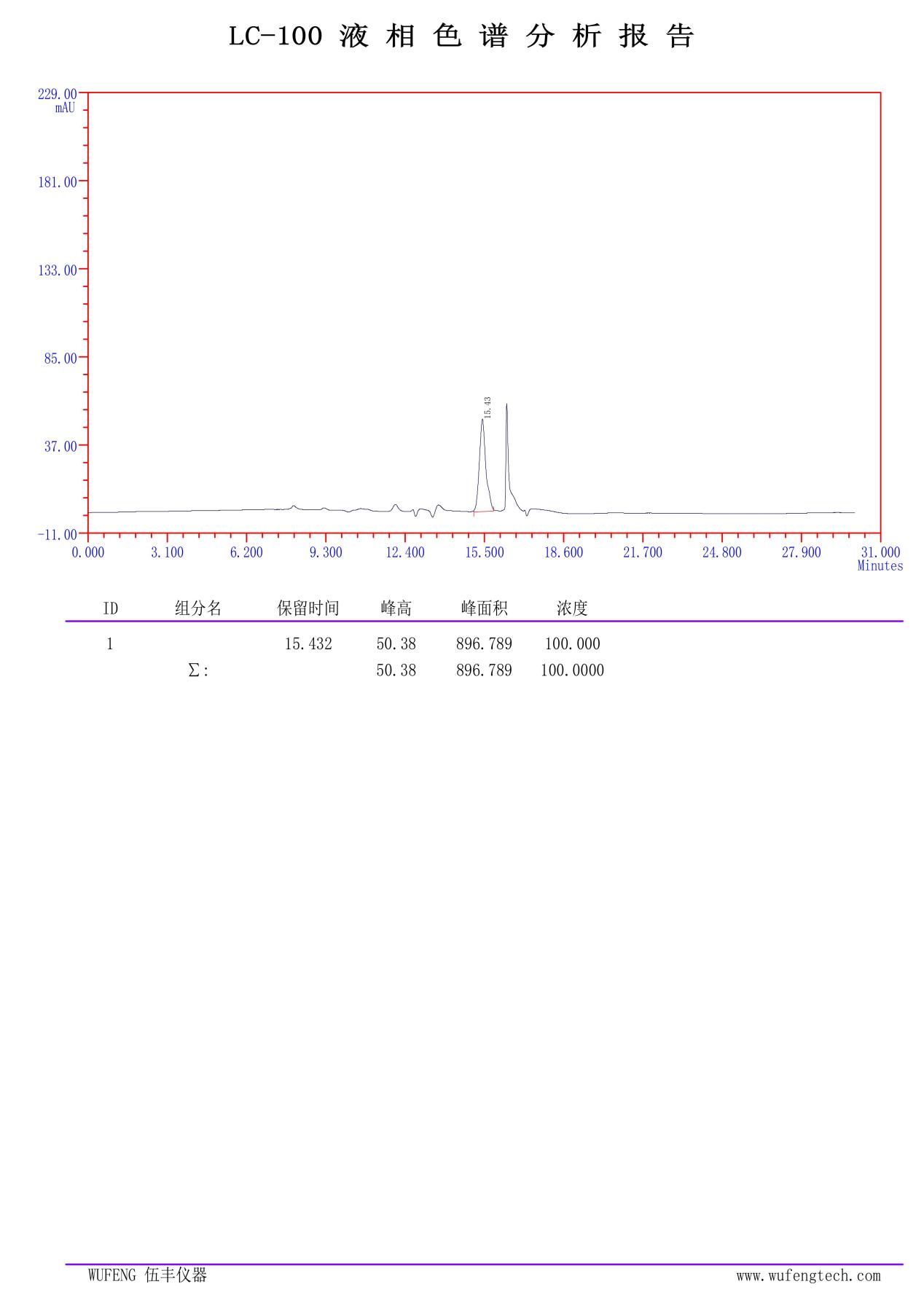


210d-ZR


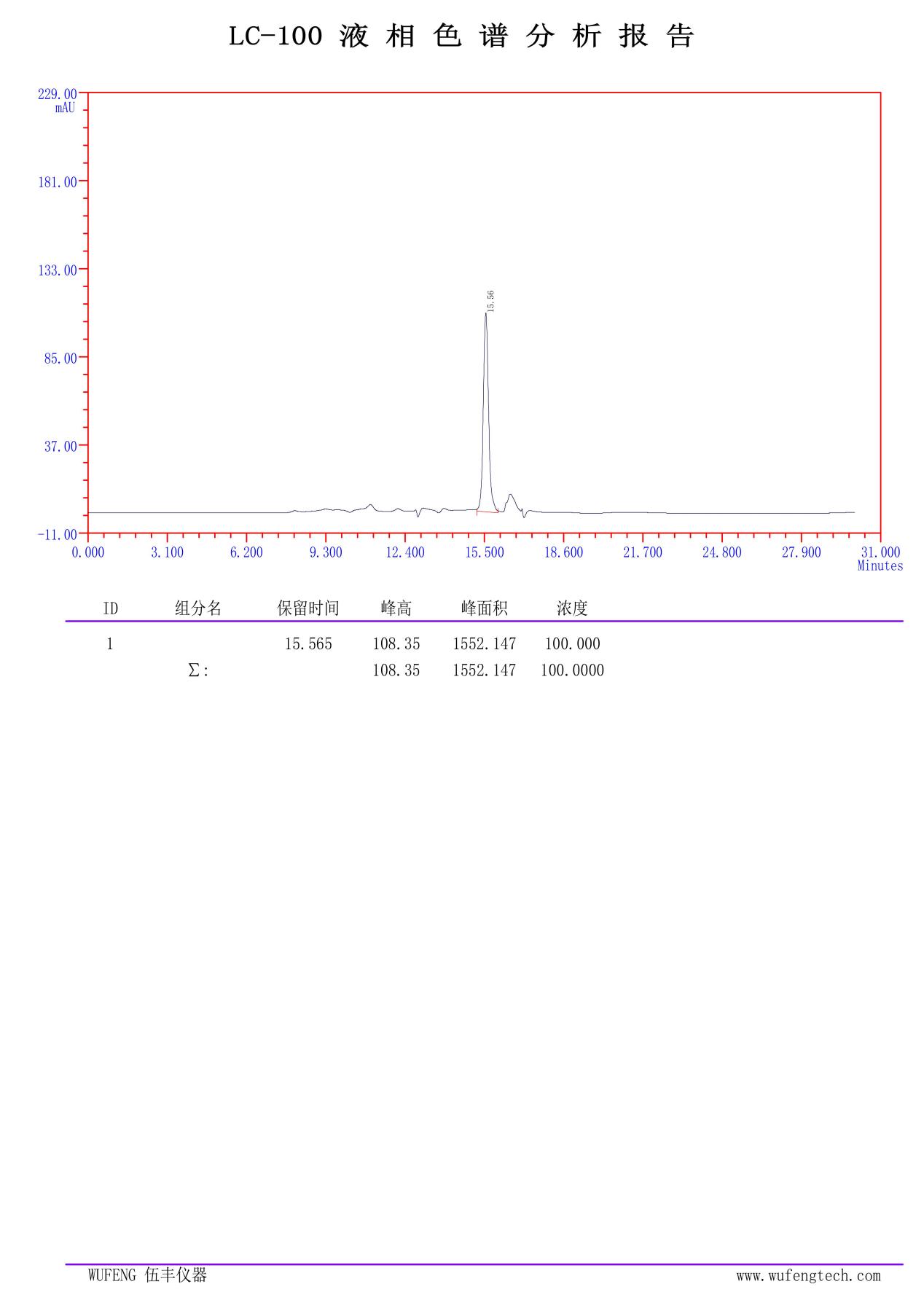


210d-ZCP


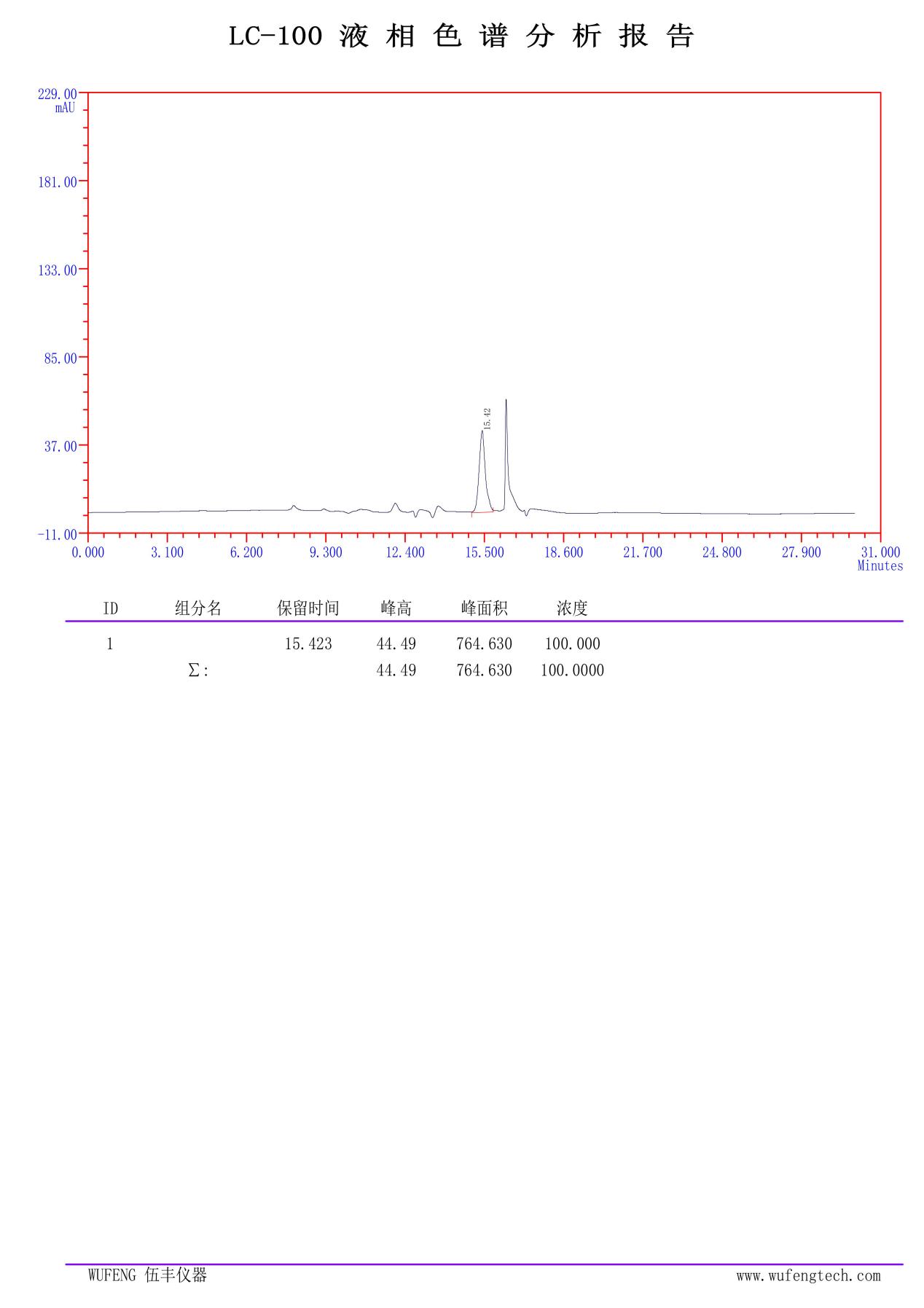


210d-ZCR


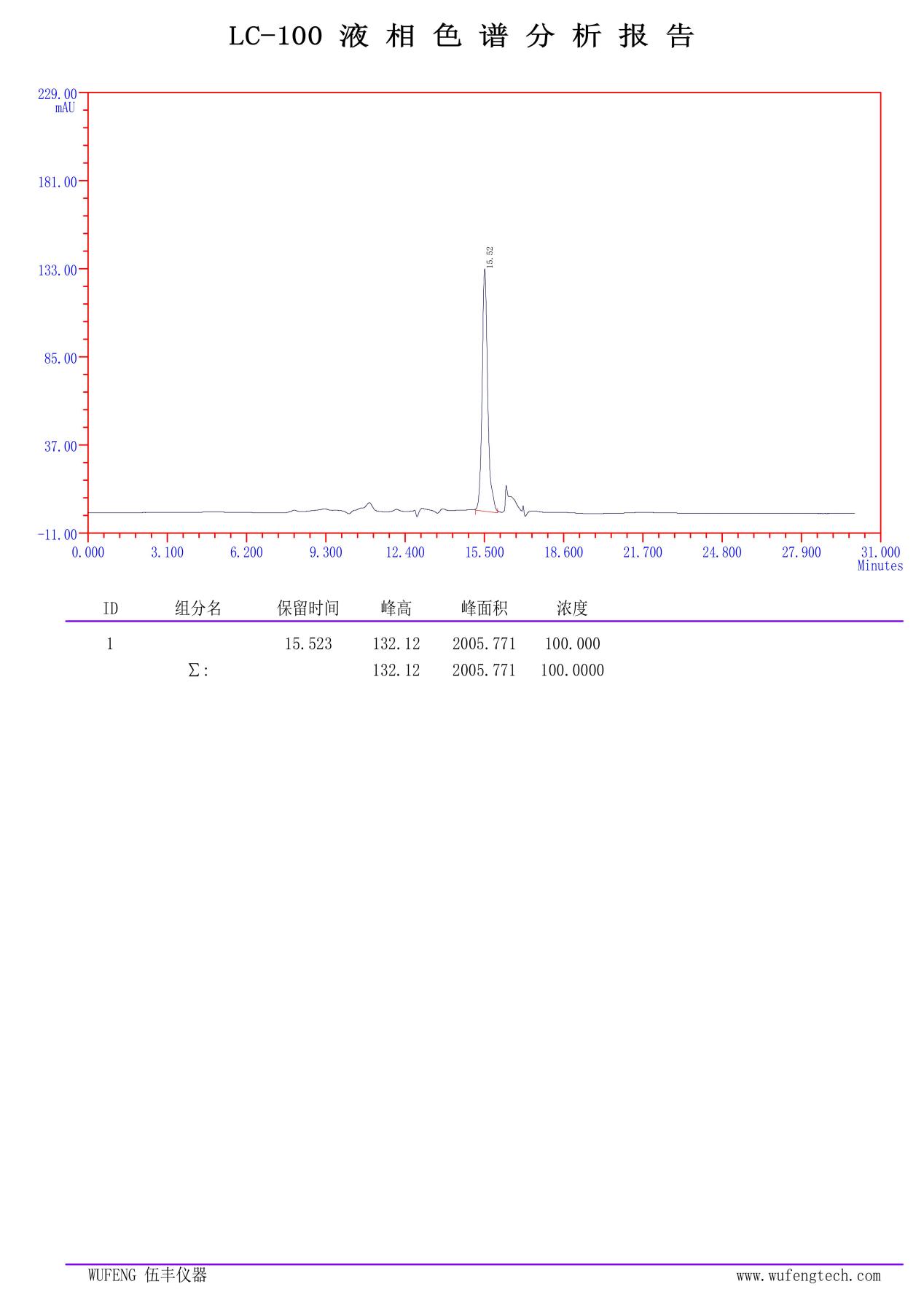


240d-HP


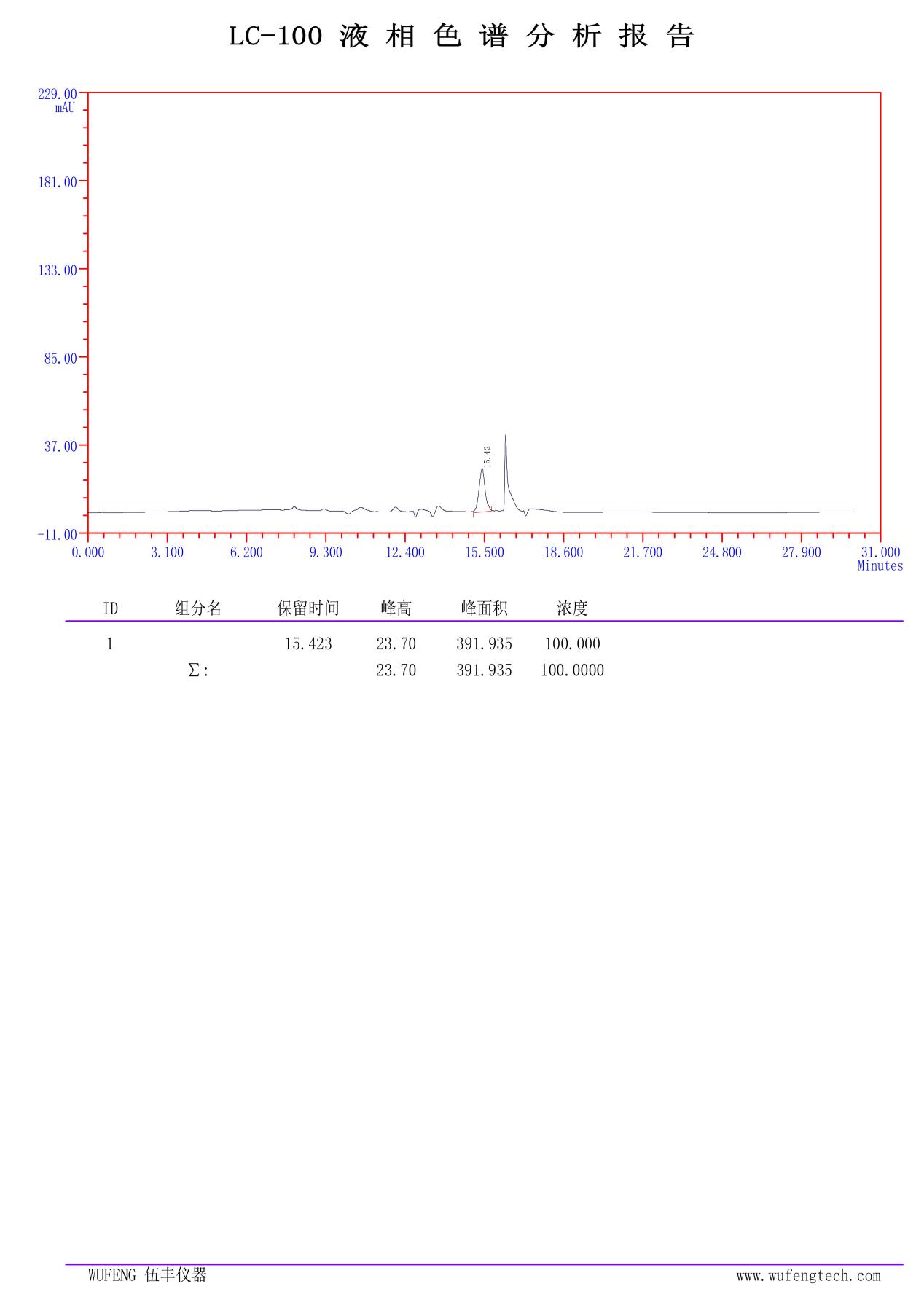


240d-HR


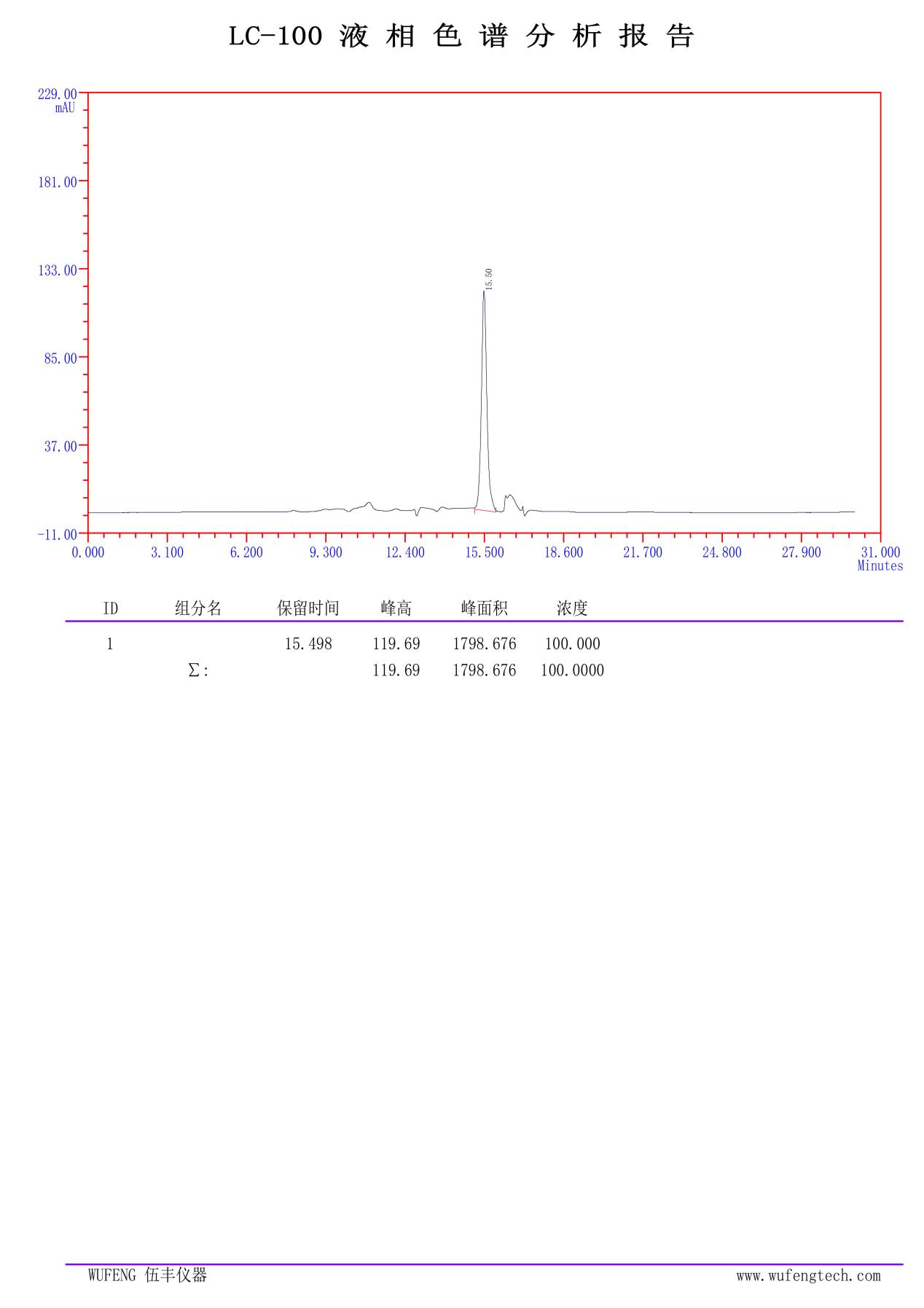


240d-XP


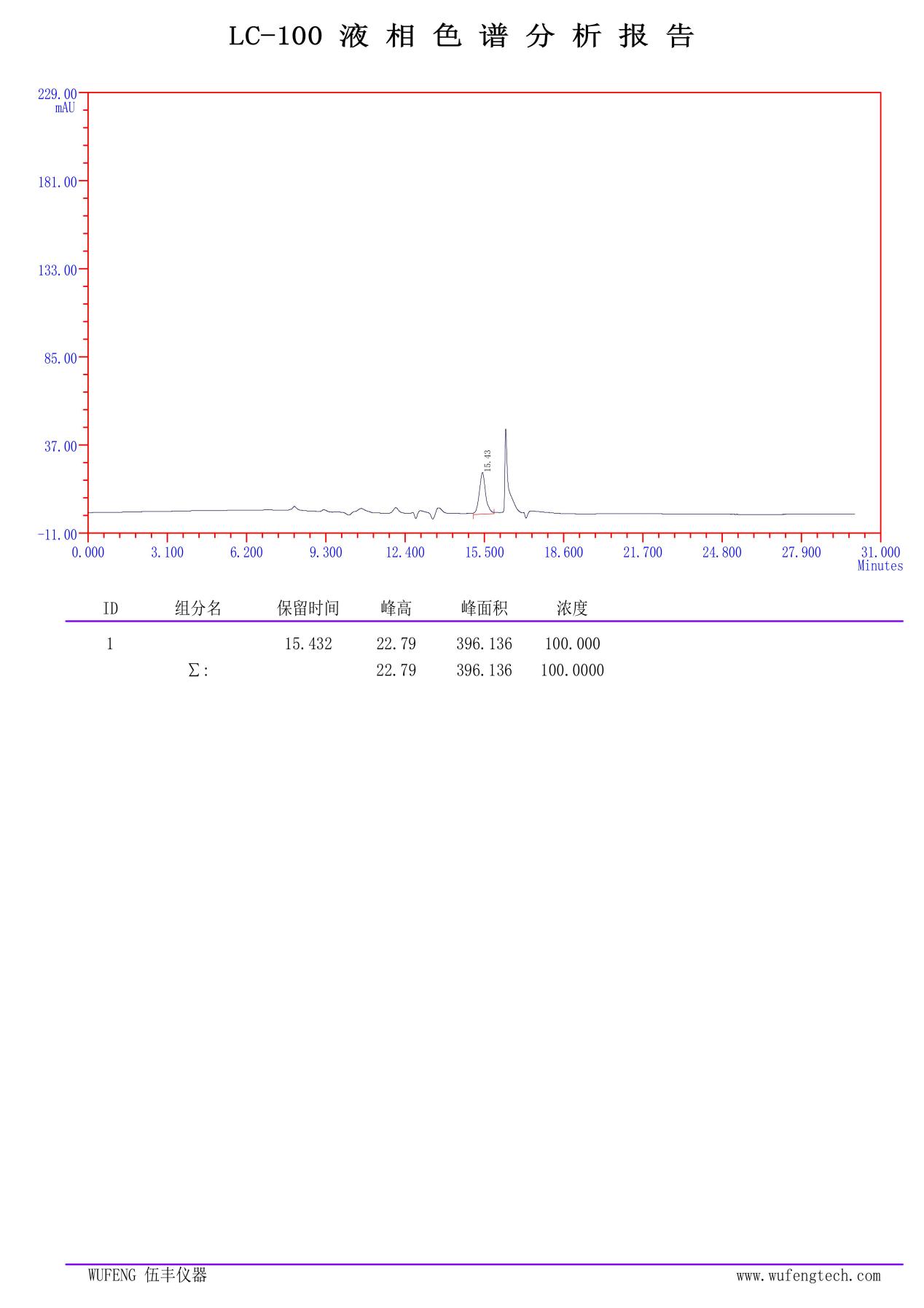


240d-XR


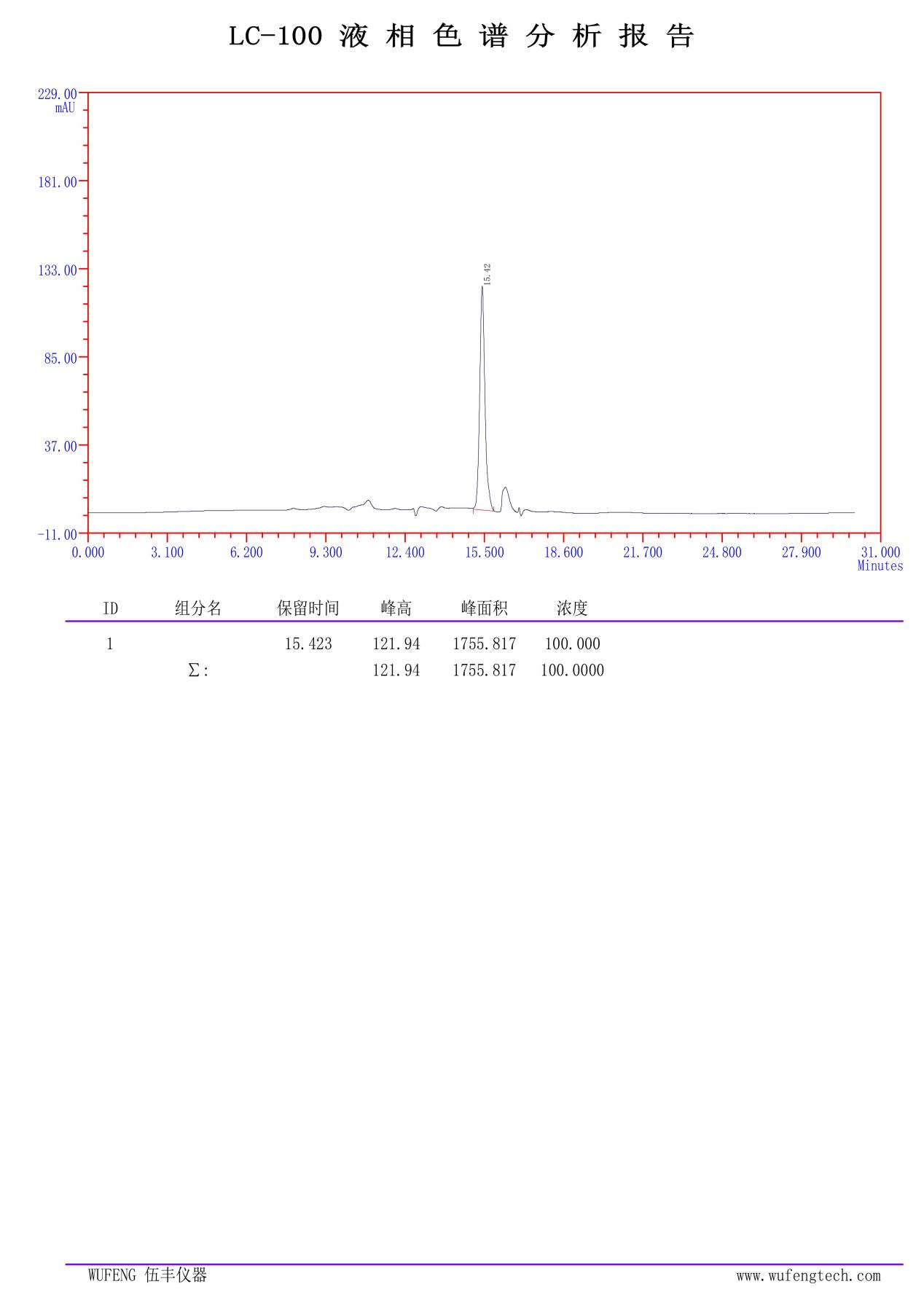


240d-ZP


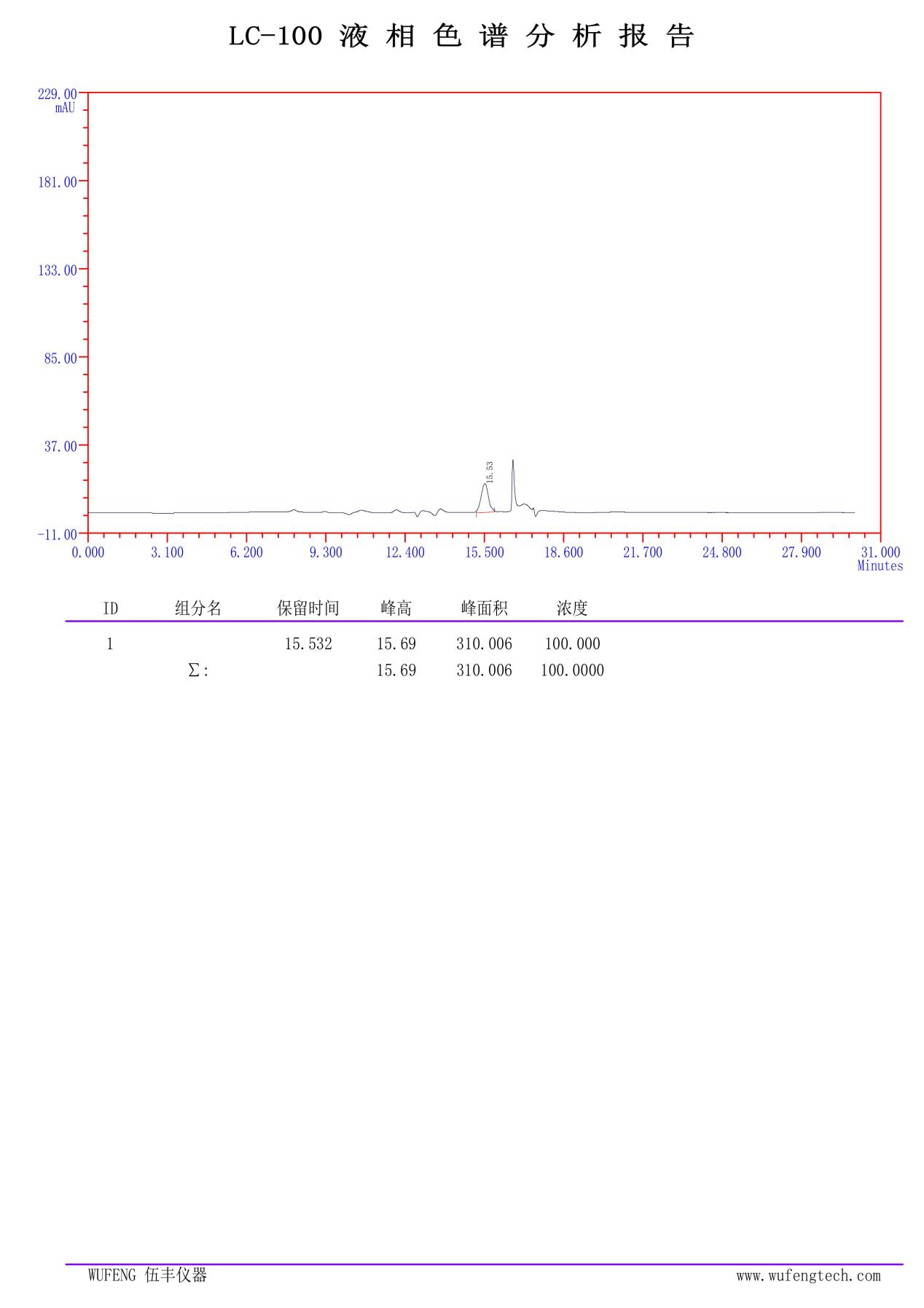


240d-ZR


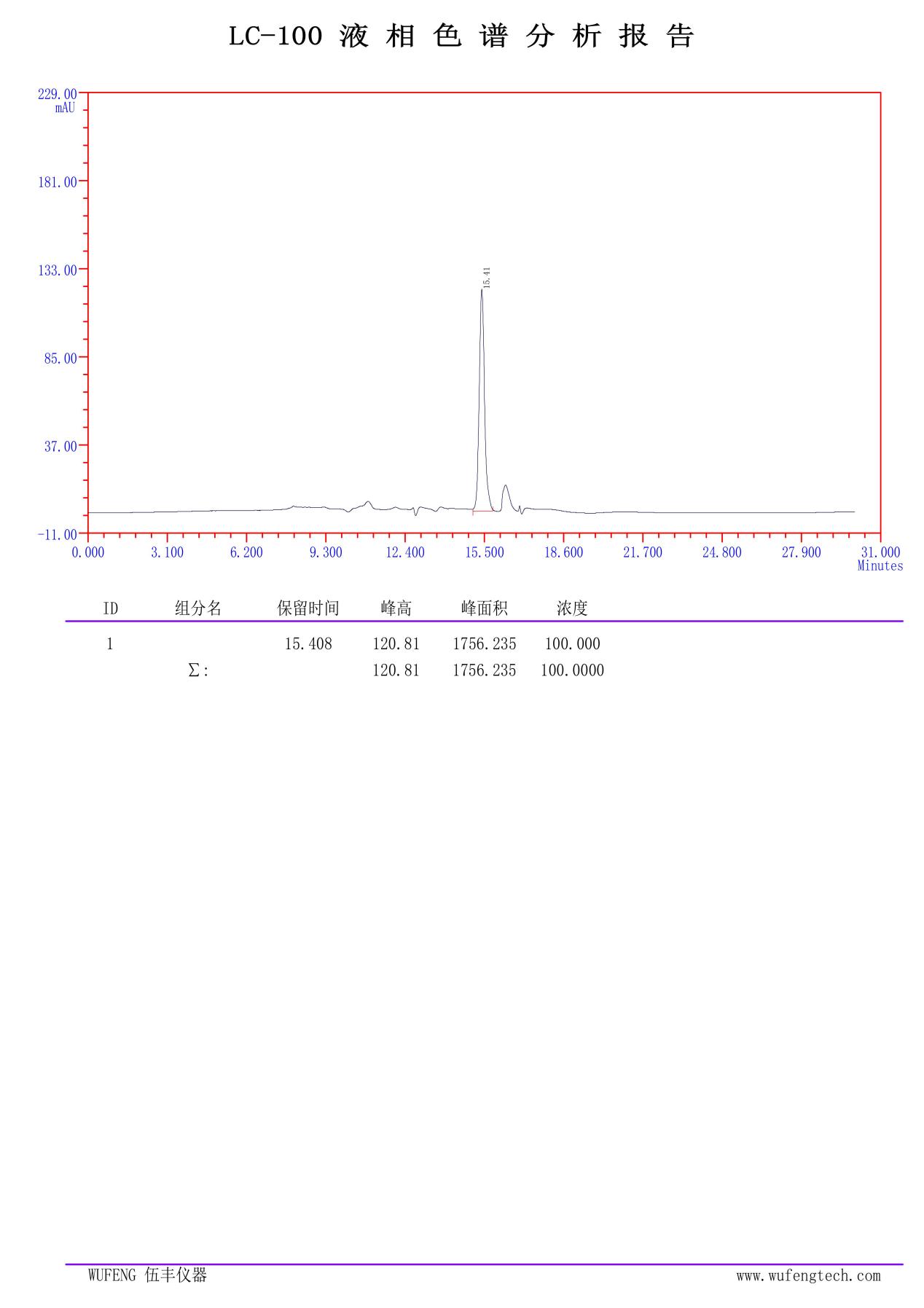


240d-ZCP


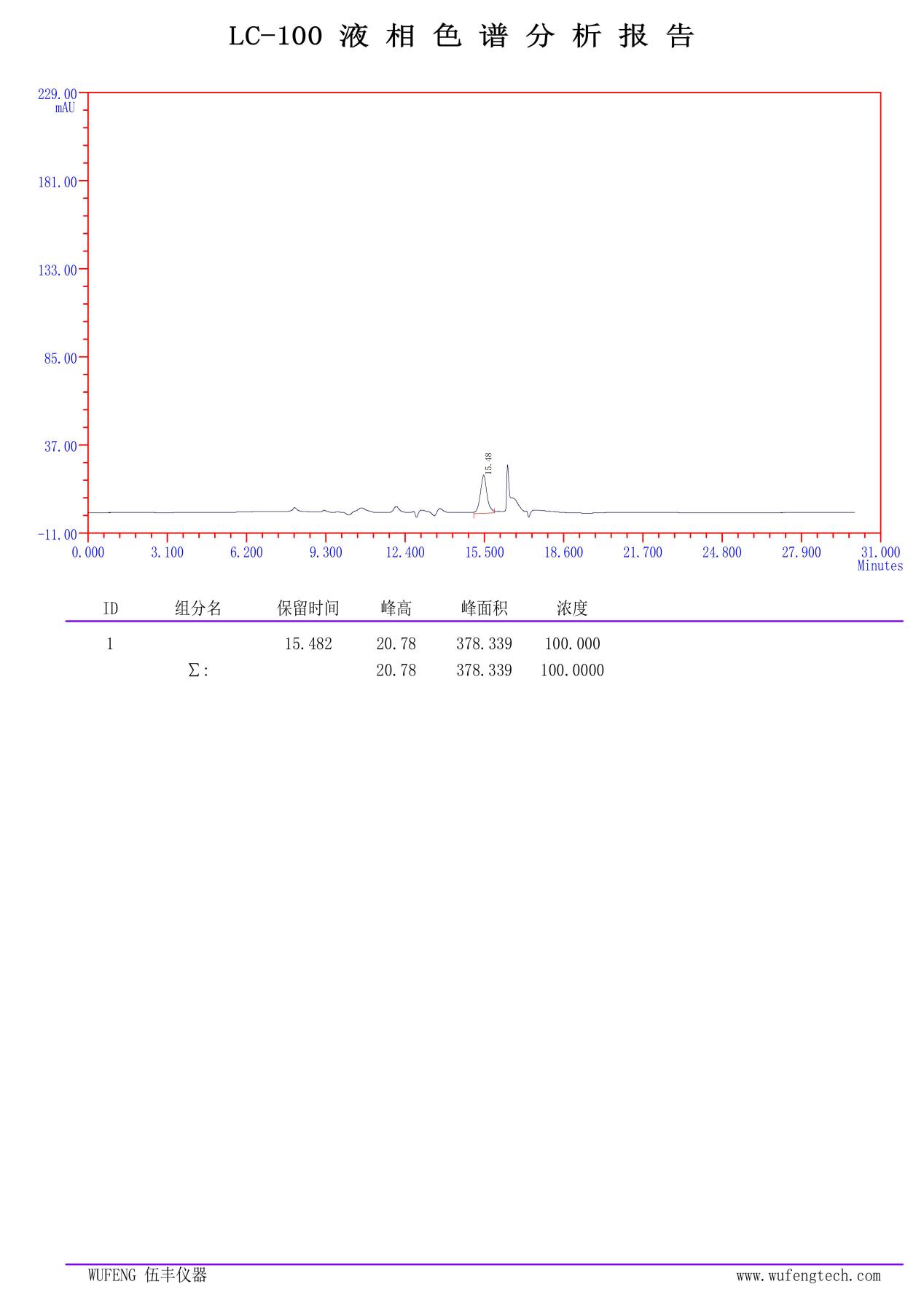


240d-ZCR


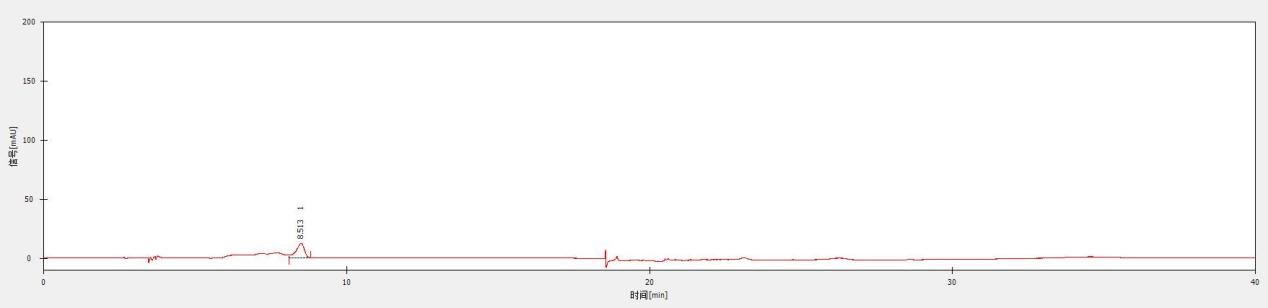


Standard 1


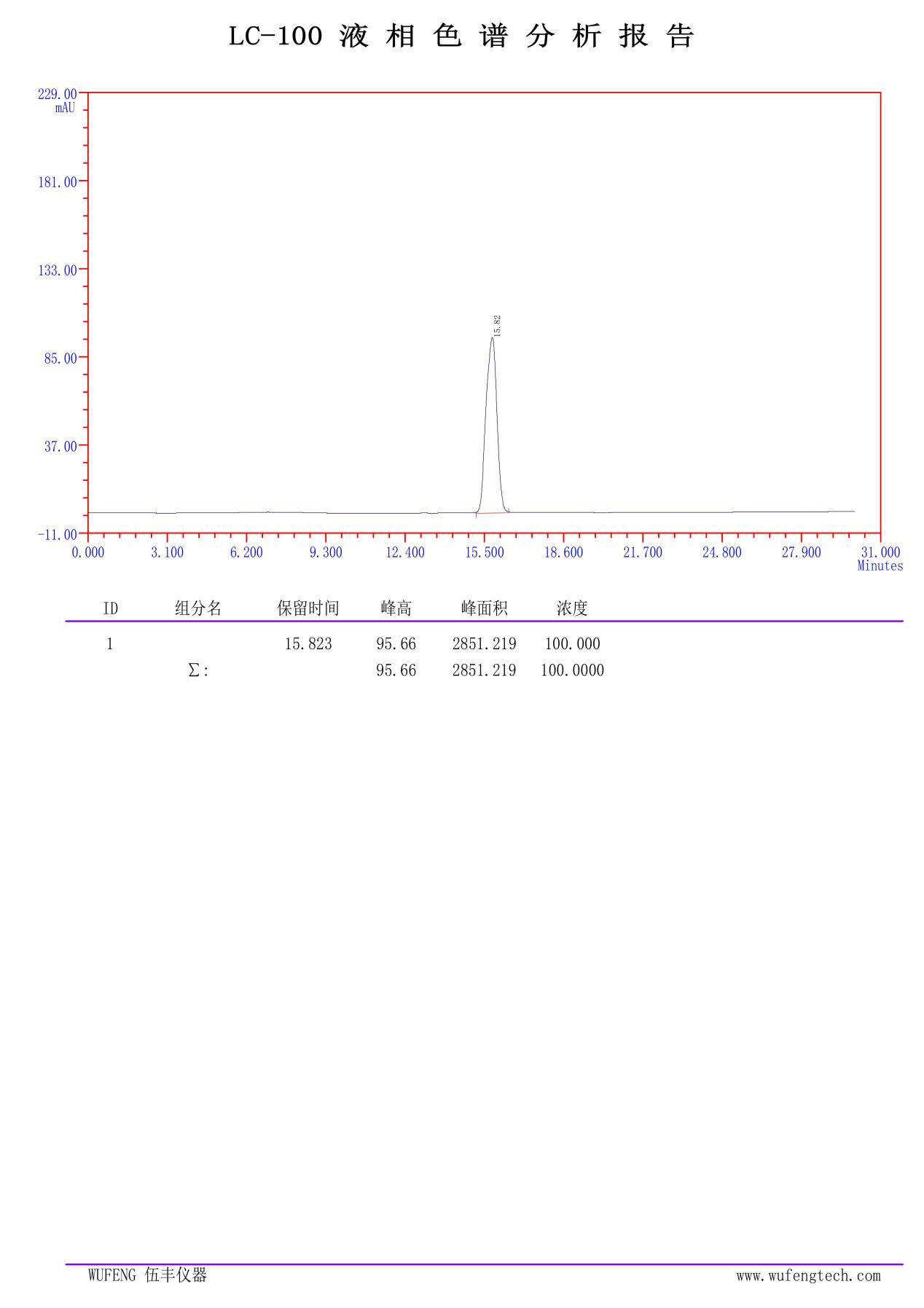


Standard 2

Fig S4 HPLC mass spectrometry of the effects of different rootstocks on the content of synephrine in 'Orah' fruit

Notes: Standard 1: standards for synephrine after 90-150 DAF; Standard 2: standards for synephrine after 180-240 DAF.
