# Supplementary material for: Effects of rootstocks and developmental time on the dynamic changes of main functional substances in ‘Orah’ (Citrus reticulata Blanco) by HPLC coupled with UV detection
Source: Front Plant Sci. 2024 Aug 27;15:1382768. doi: 10.3389/fpls.2024.1382768 (PMC11388320; doi:10.3389/fpls.2024.1382768)
Supplement: Supplementary file 6 [file Table4.docx]

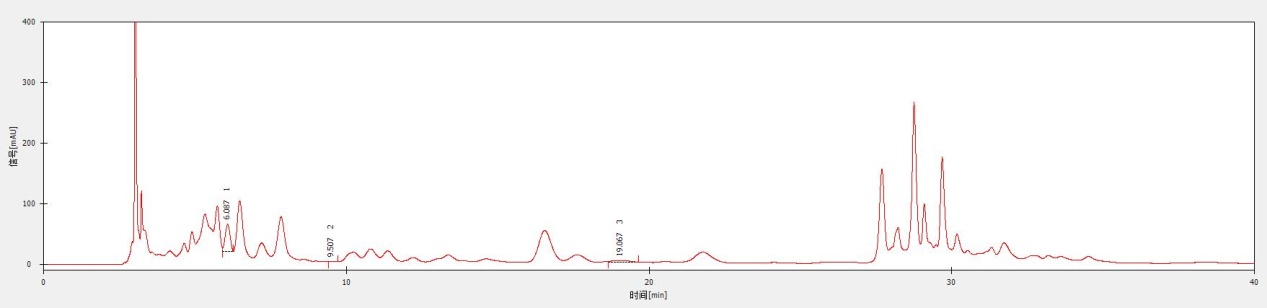


90d-HP


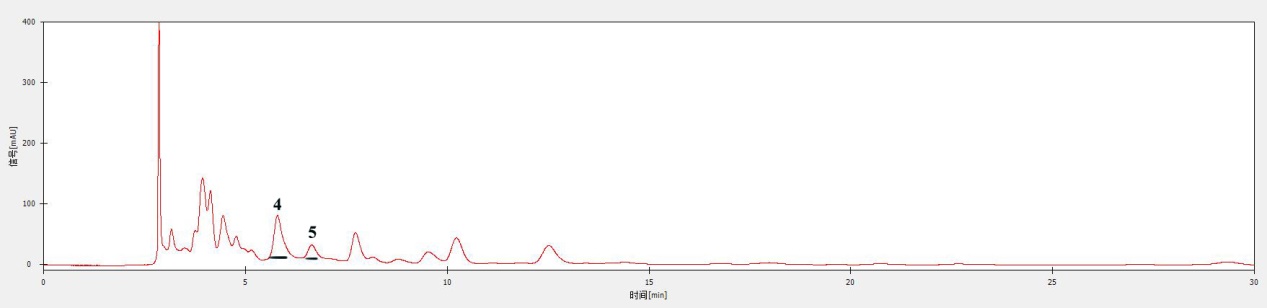


90d-HP


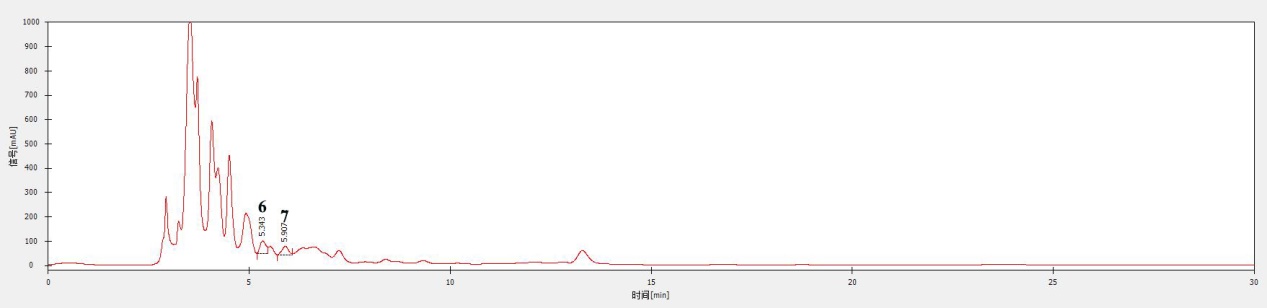


90d-HP


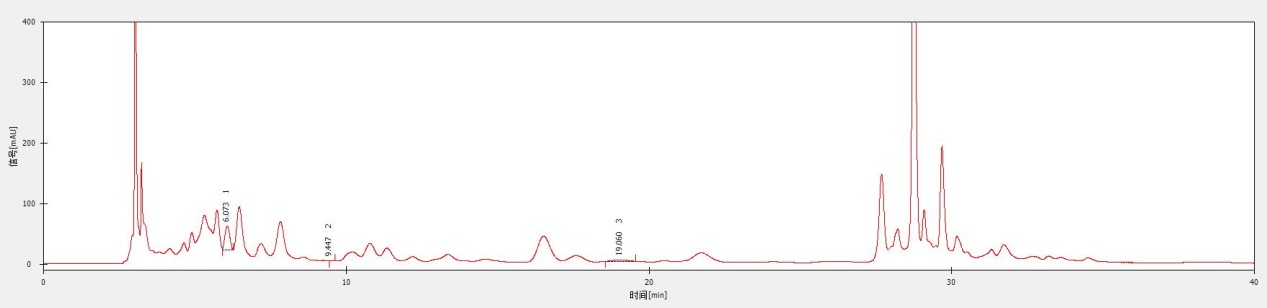


90d-HR


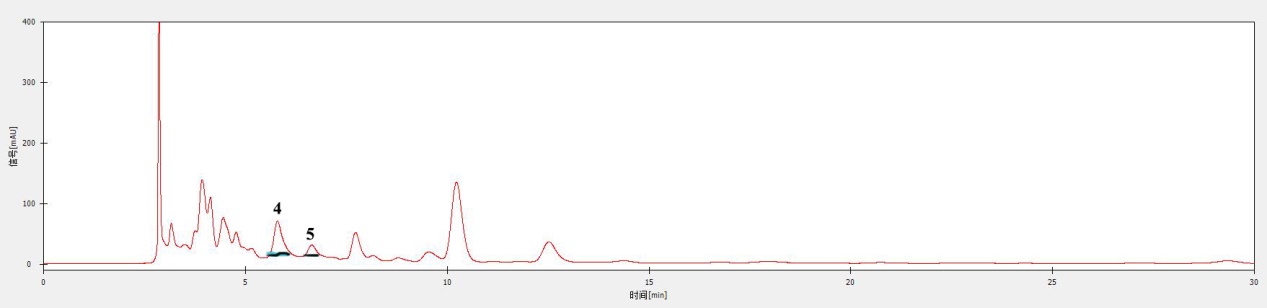


90d-HR


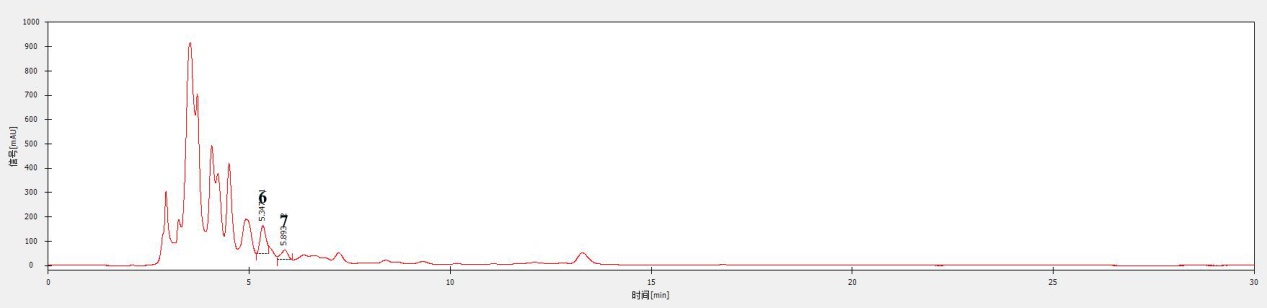


90d-HR


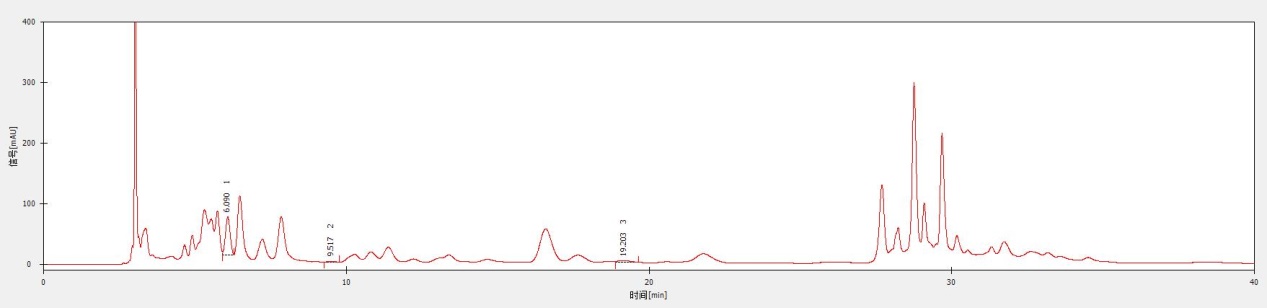


90d-XP

90d-XP


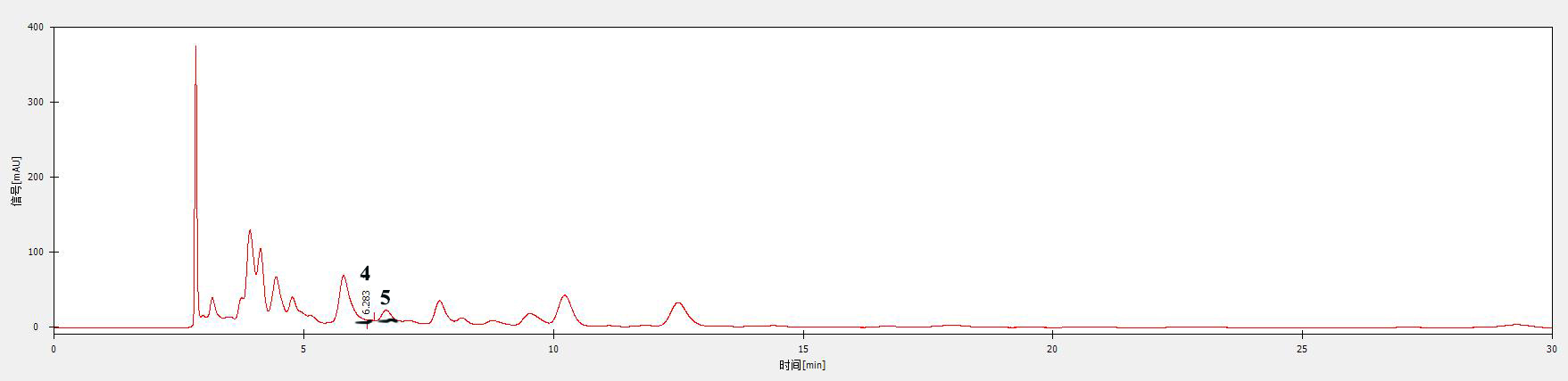


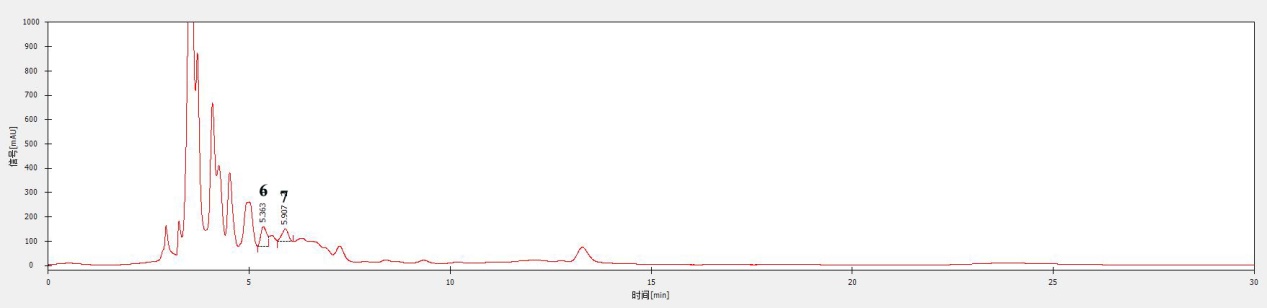


90d-XP


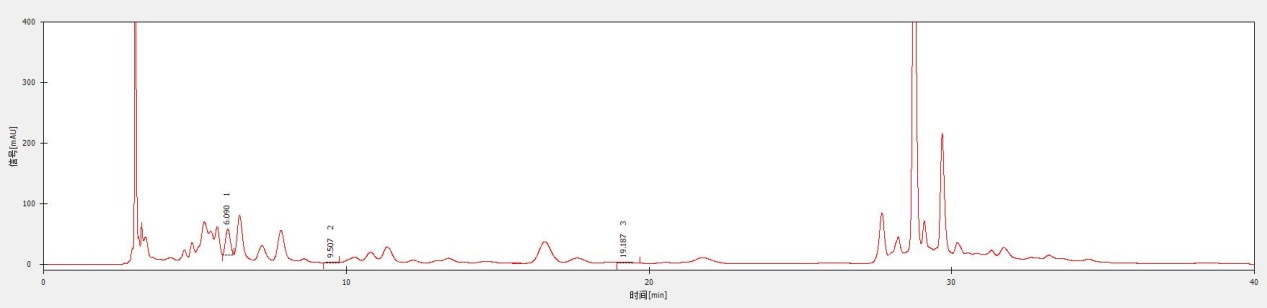


90d-XR


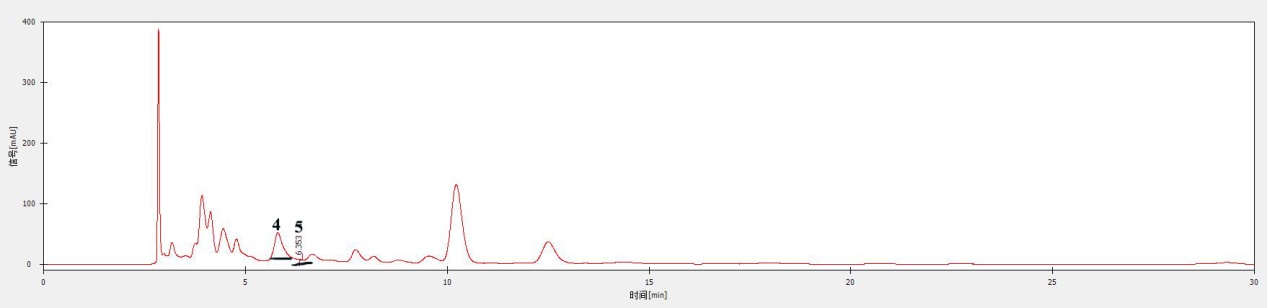


90d-XR


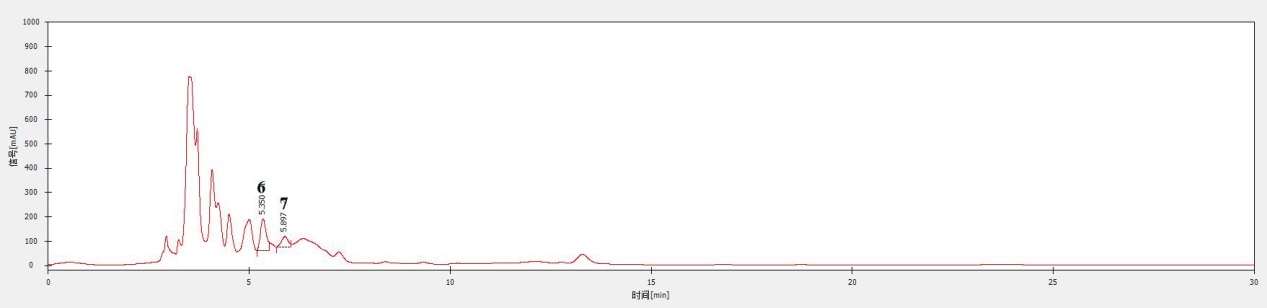


90d-XR


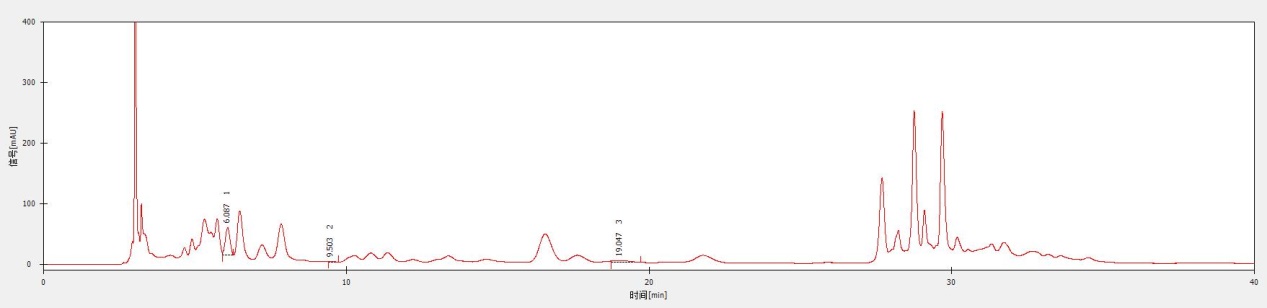


90d-ZP


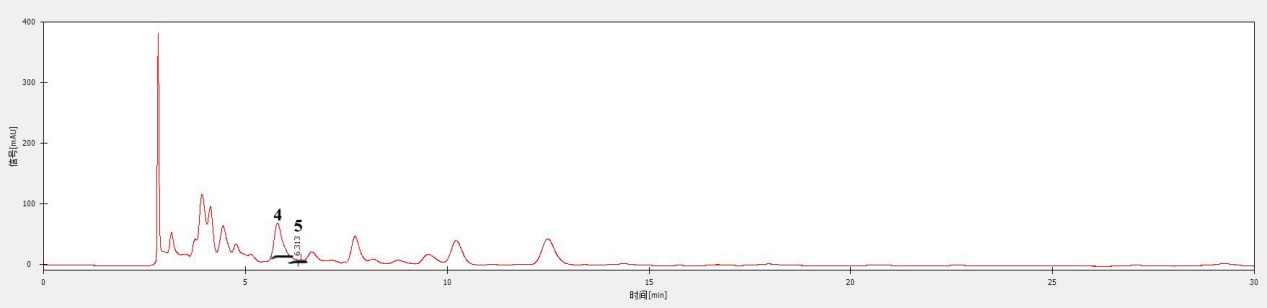


90d-ZP


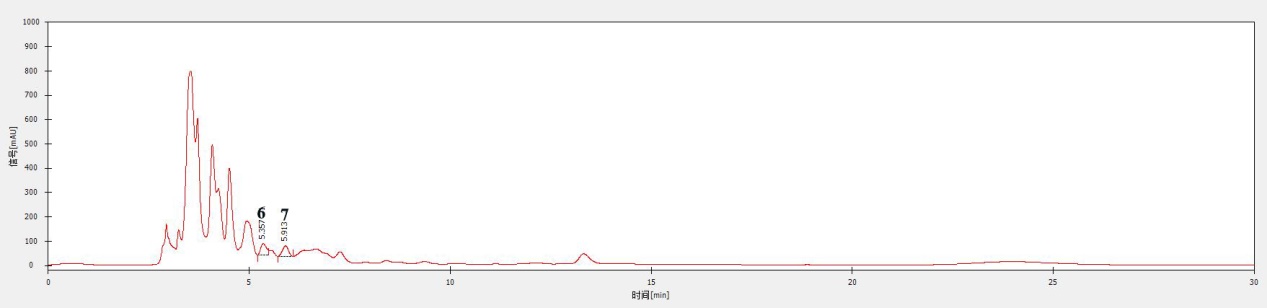


90d-ZP


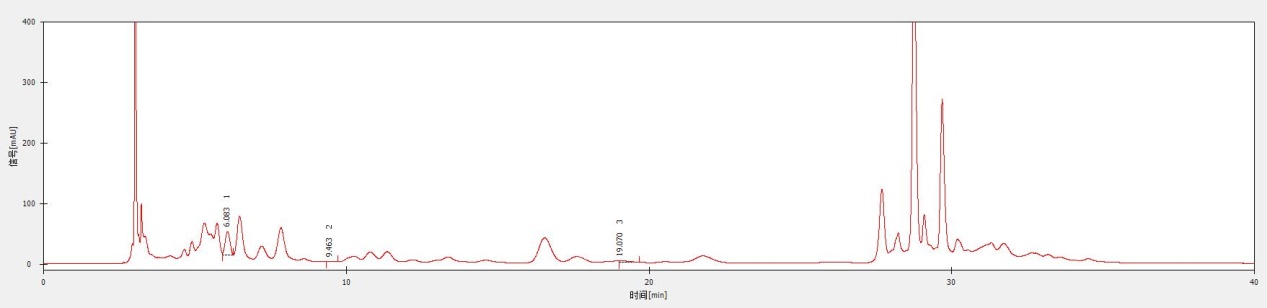


90d-ZR


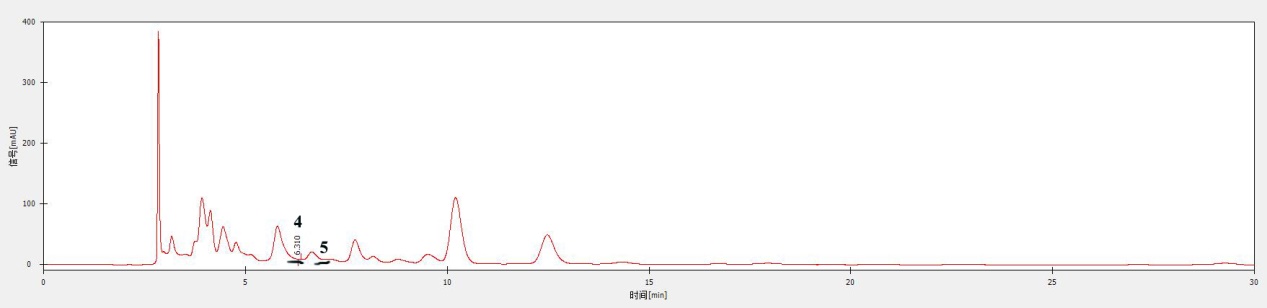


90d-ZR


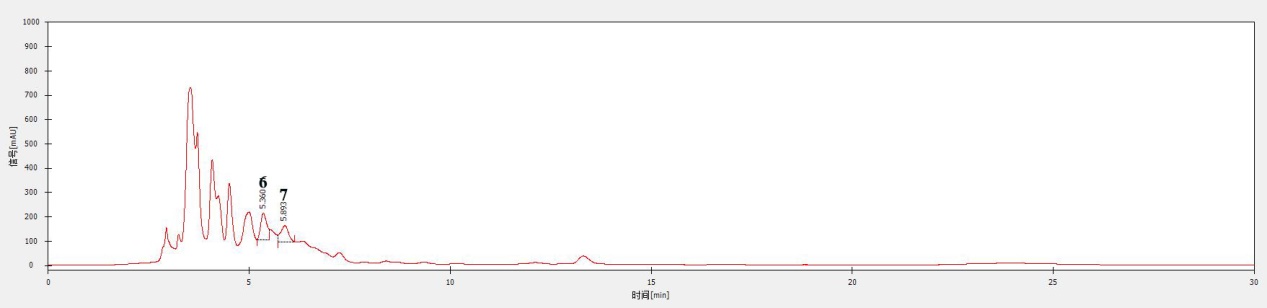


90d-ZR


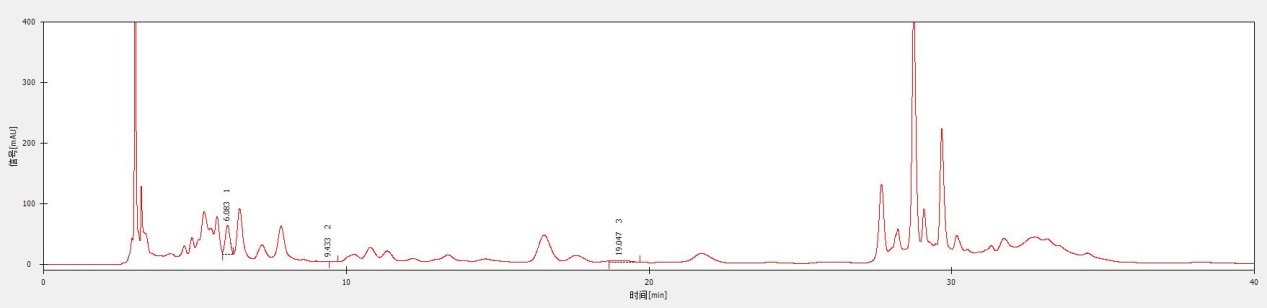


90d-ZCP


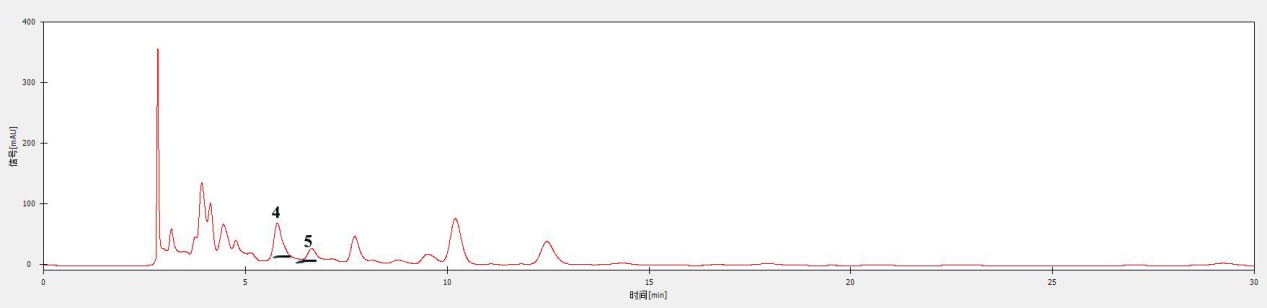


90d-ZCP


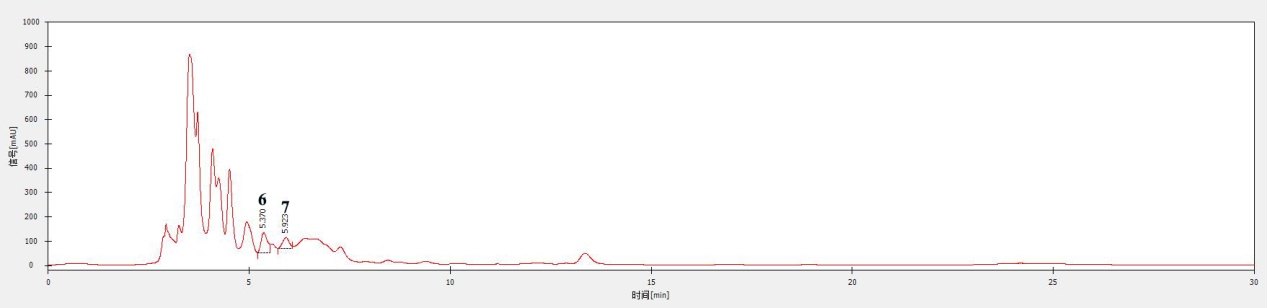


90d-ZCP


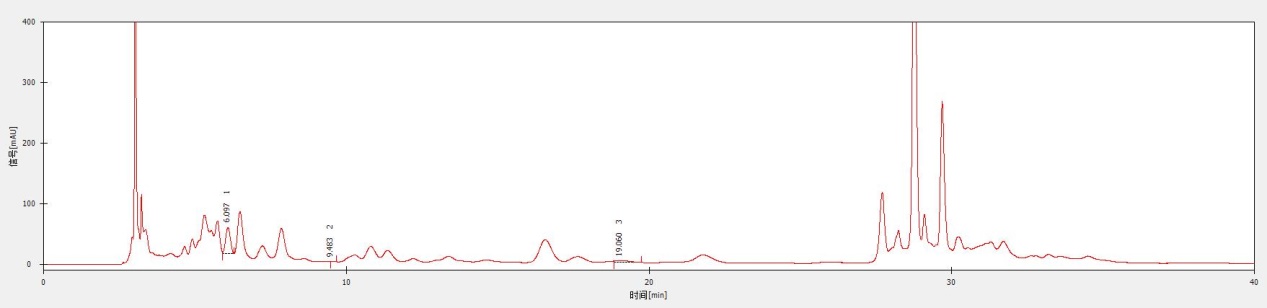


90d-ZCR


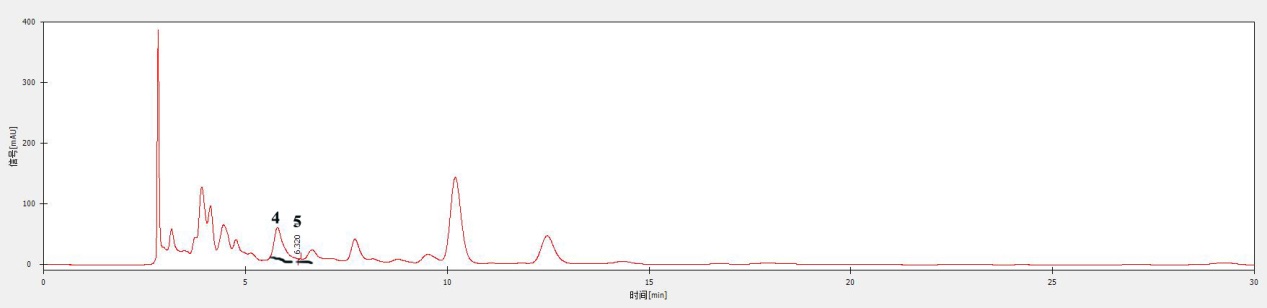


90d-ZCR


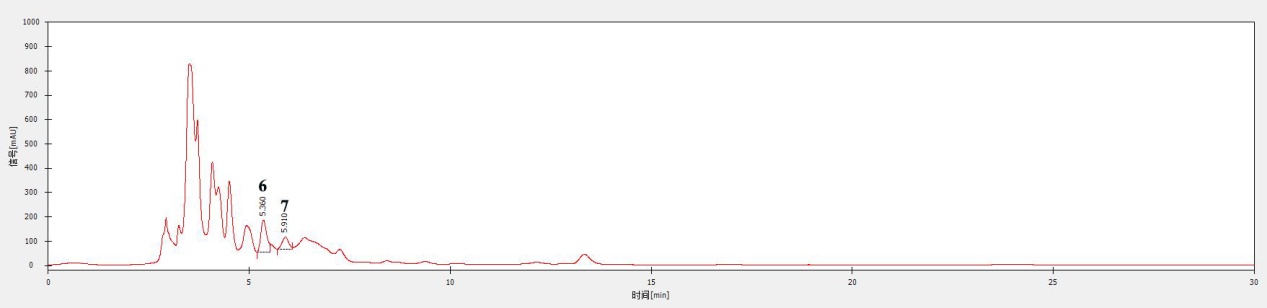


90d-ZCR


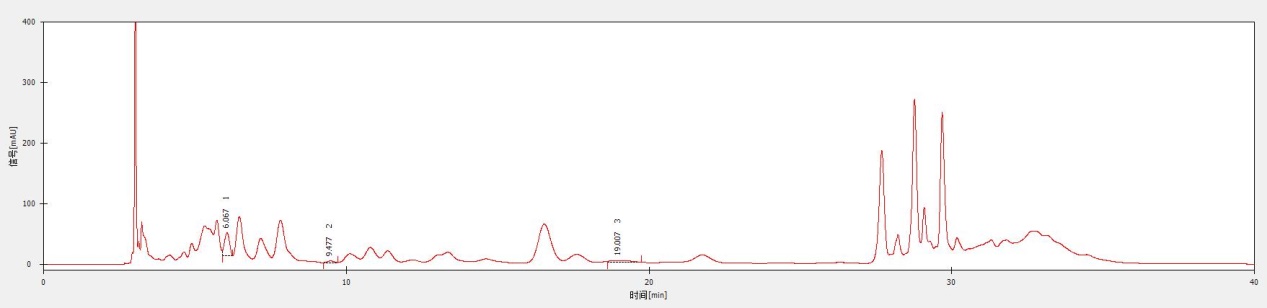


120d-HP


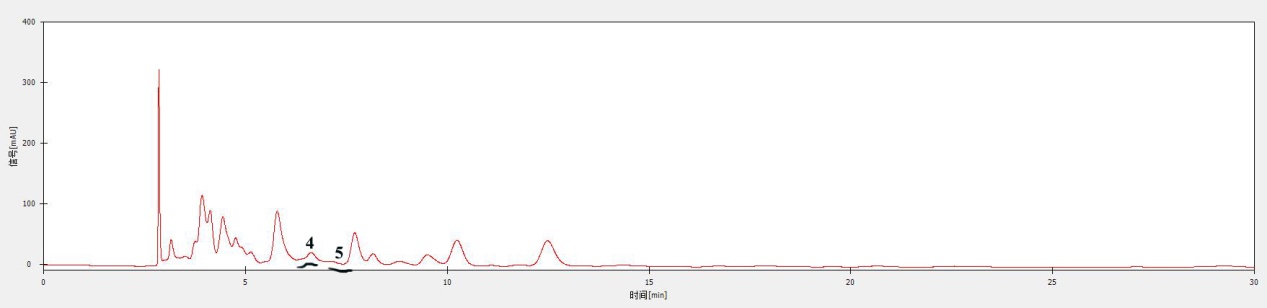


120d-HP


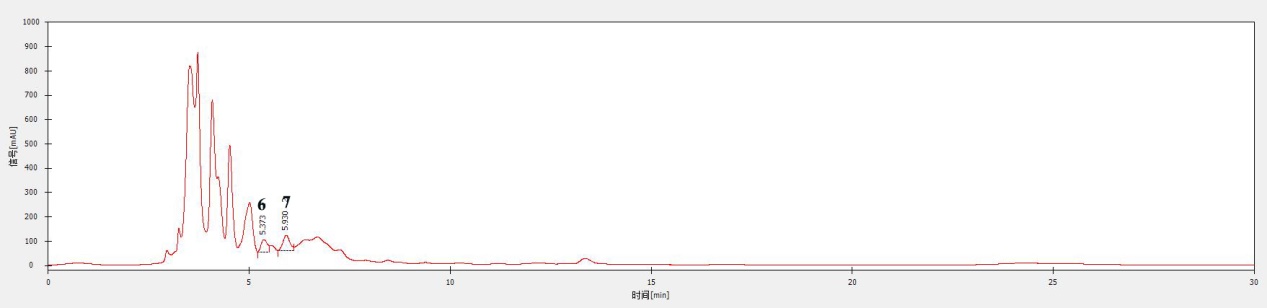


120d-HP


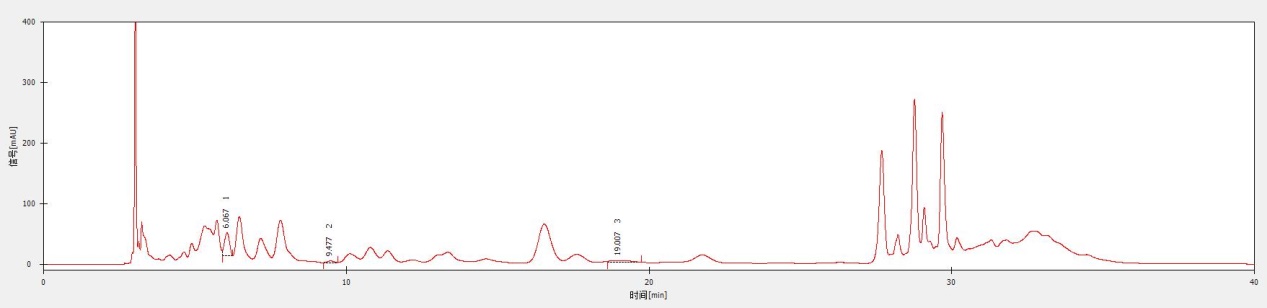


120d-HR


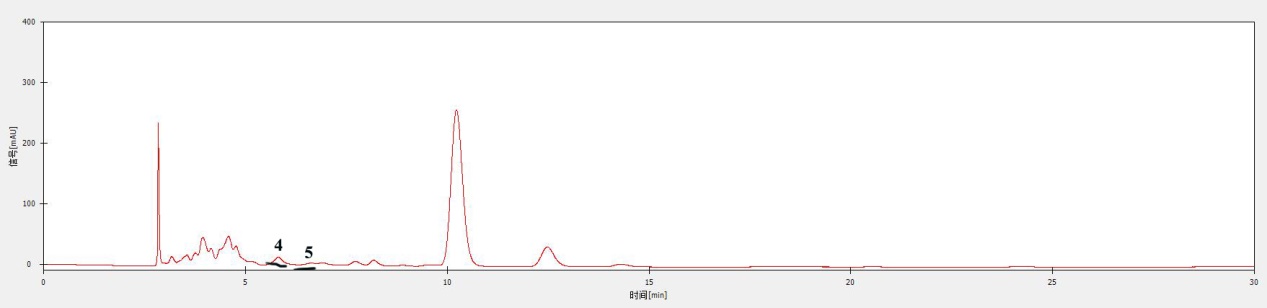


120d-HR


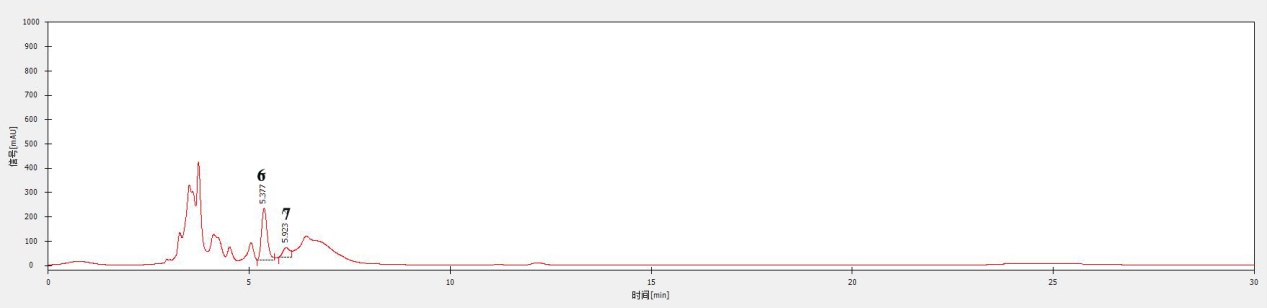


120d-HR


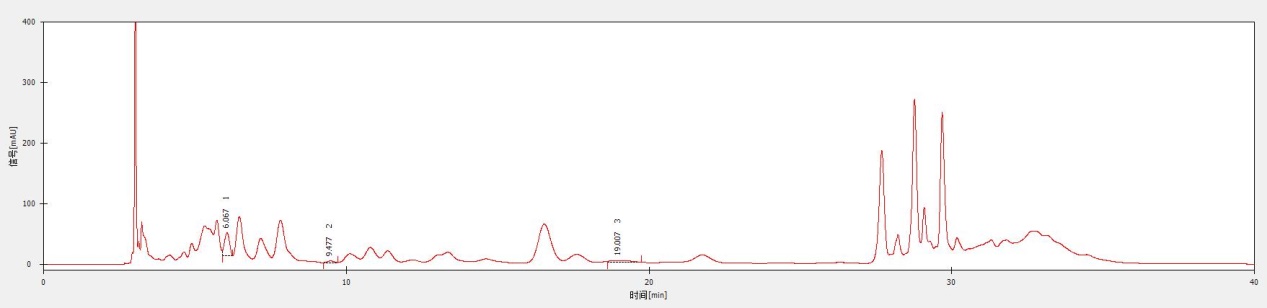


120d-XP


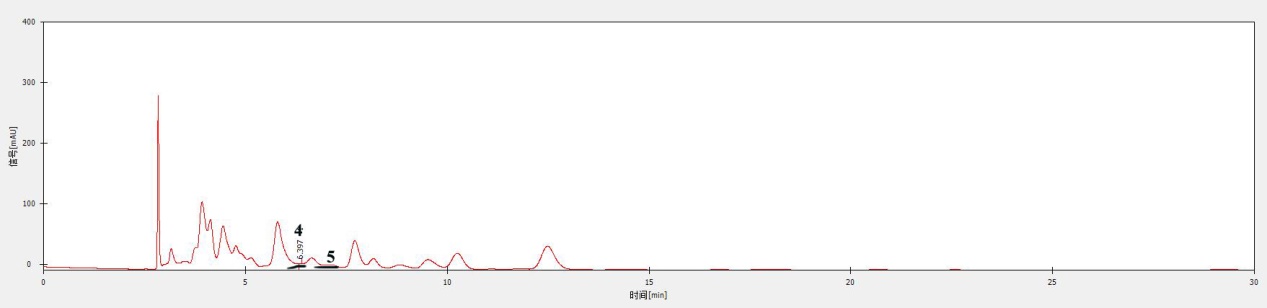


120d-XP


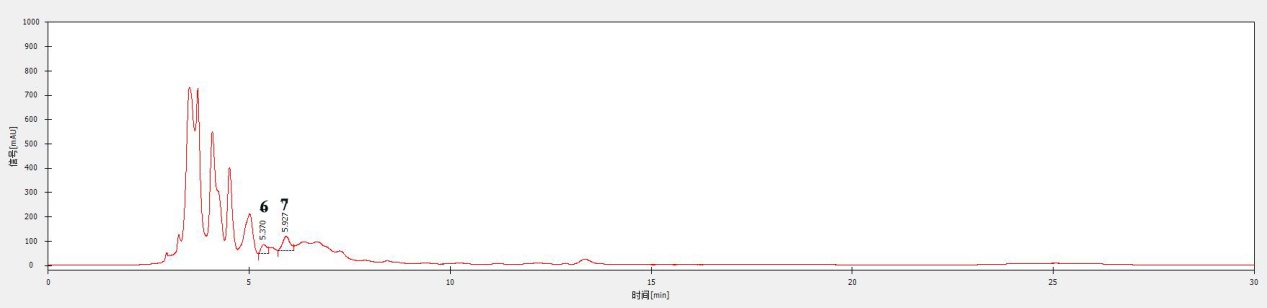


120d-XP


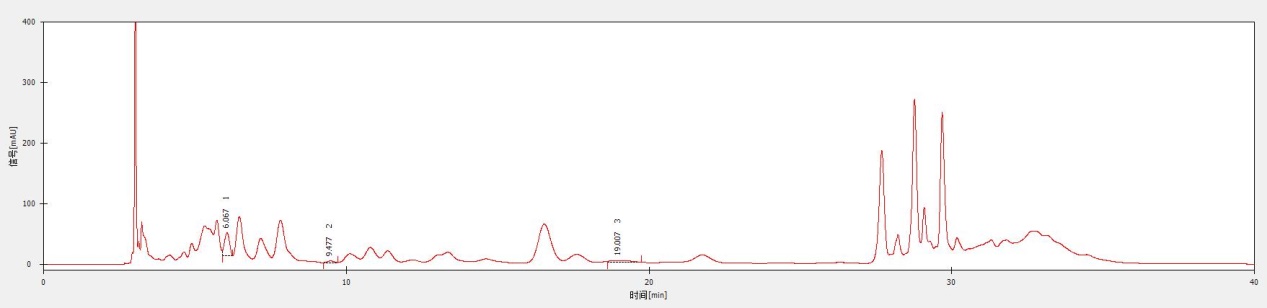


120d-XR


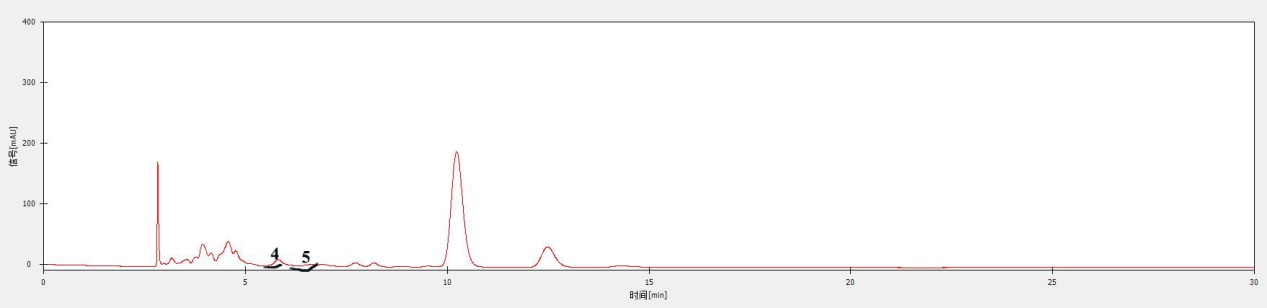


120d-XR


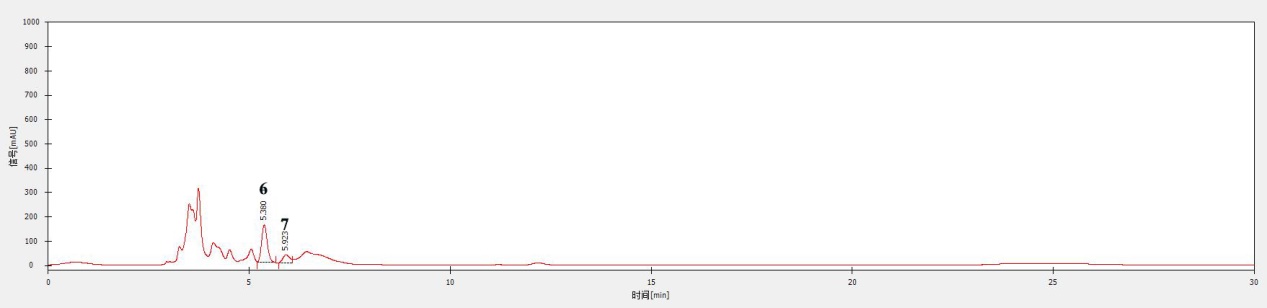


120d-XR


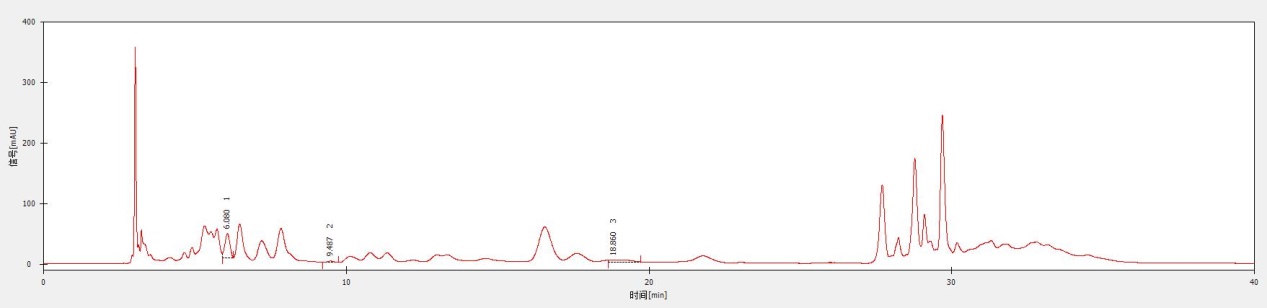


120d-ZP


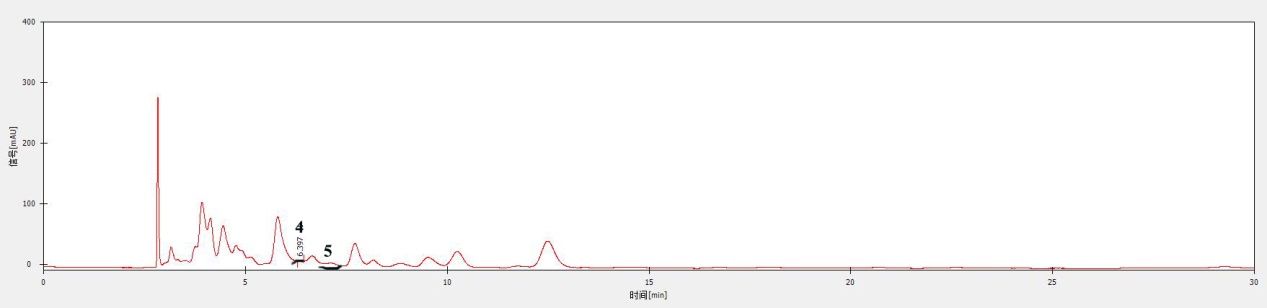


120d-ZP


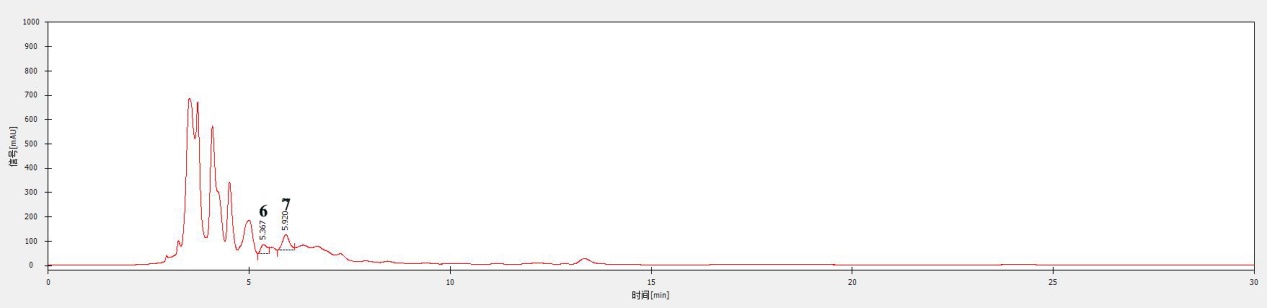


120d-ZP


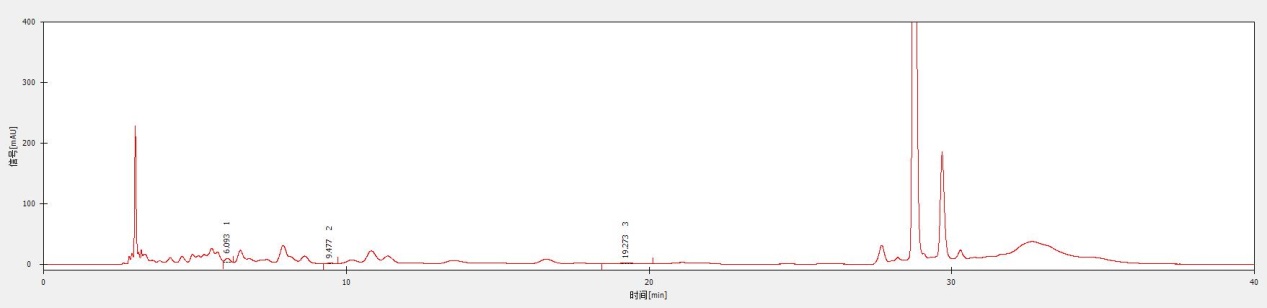


120d-ZR


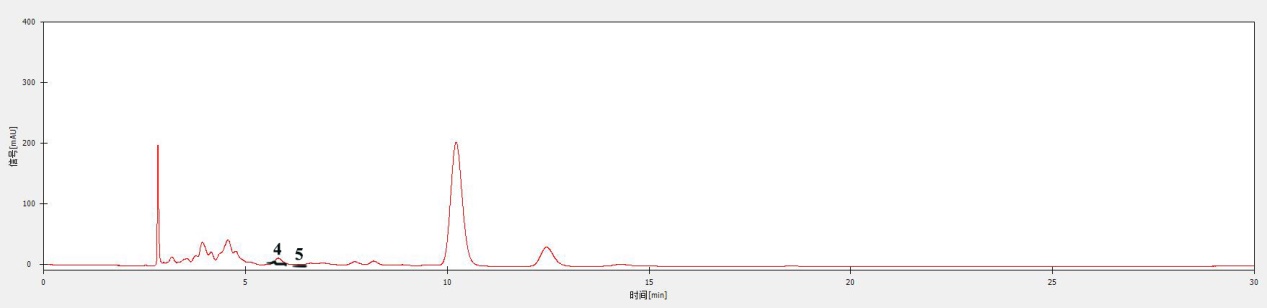


120d-ZR


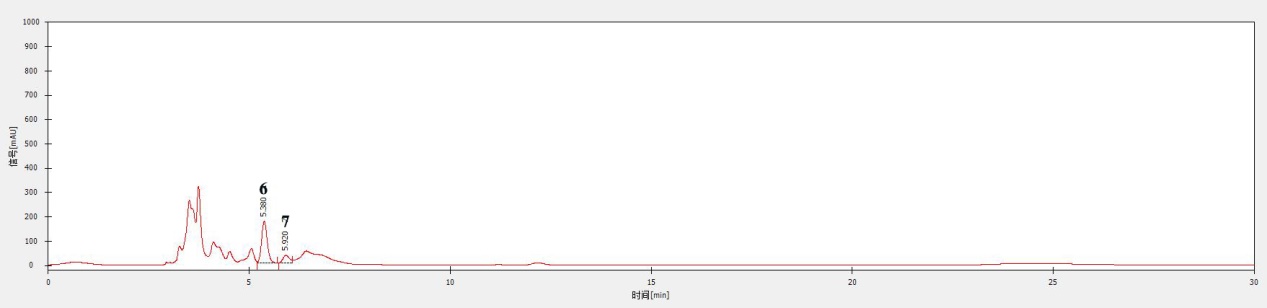


120d-ZR


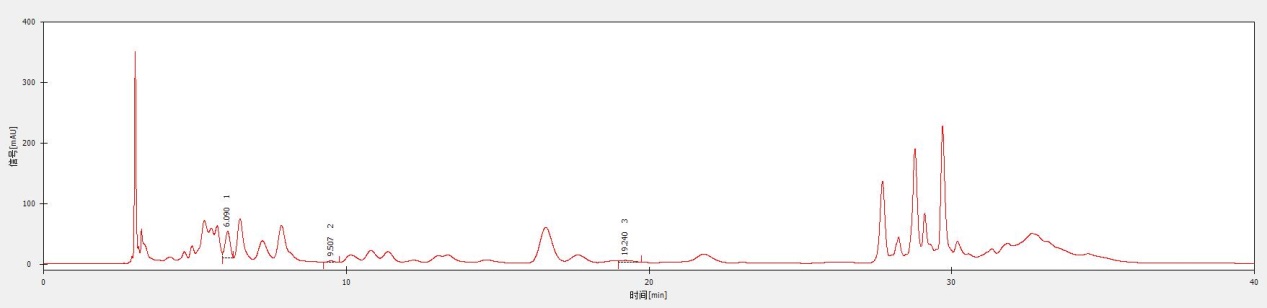


120d-ZCP


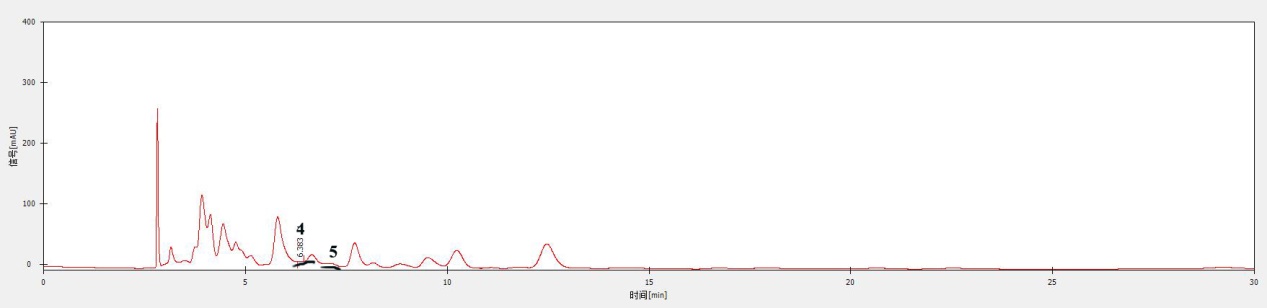


120d-ZCP


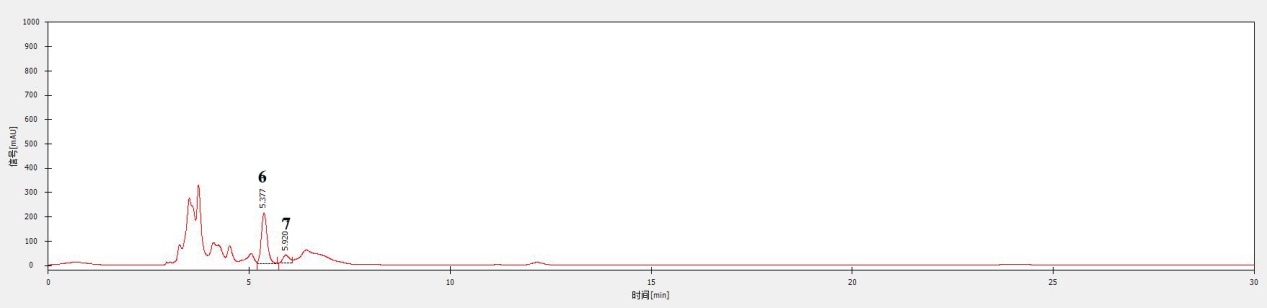


120d-ZCP


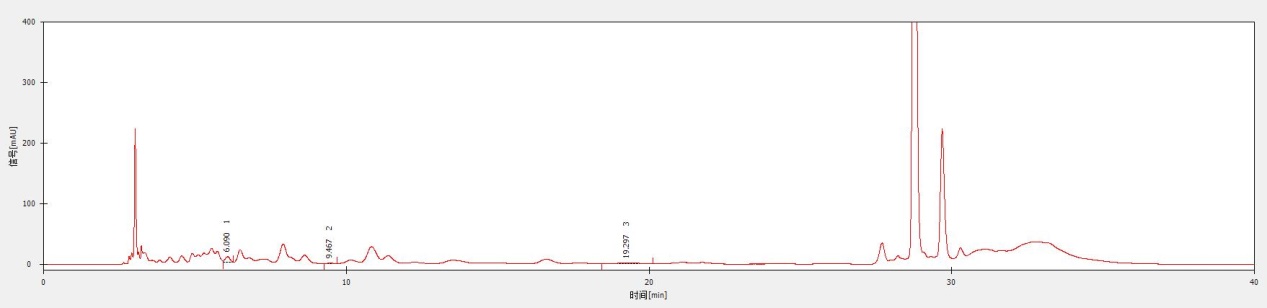


120d-ZCR


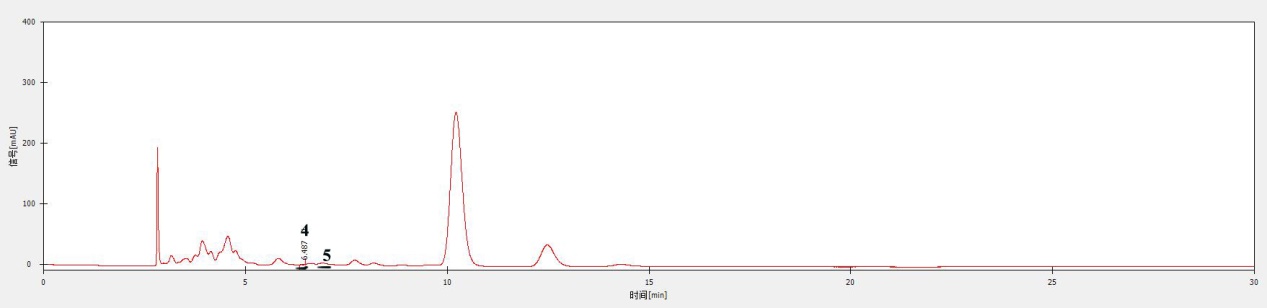


120d-ZCR


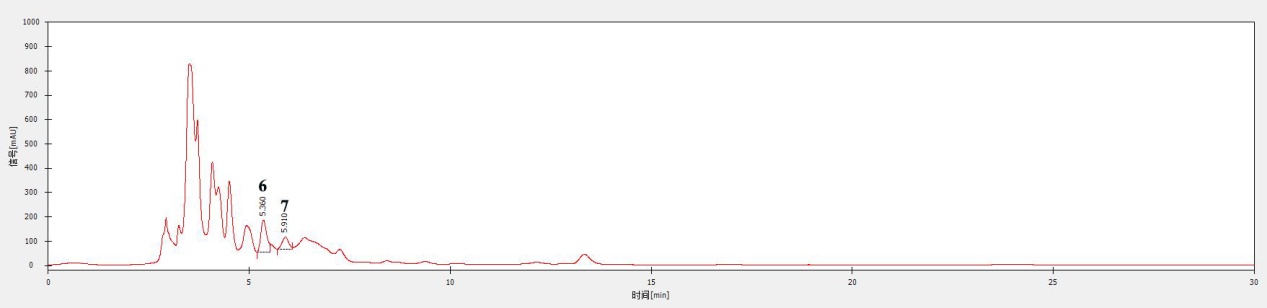


120d-ZCR


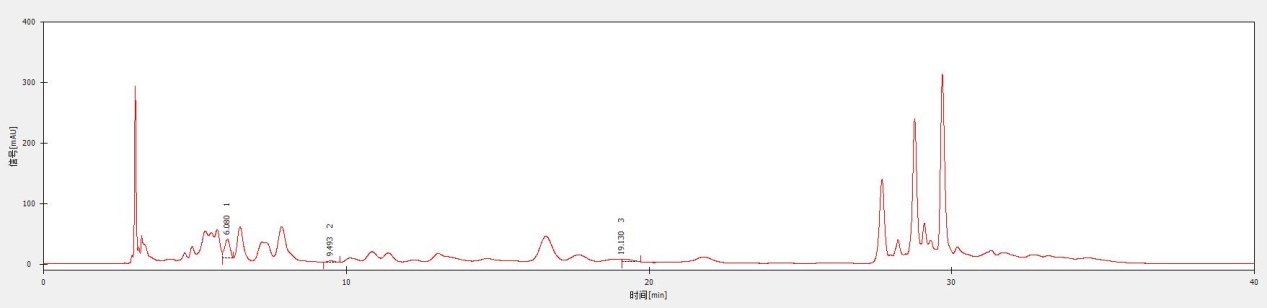


150d-HP


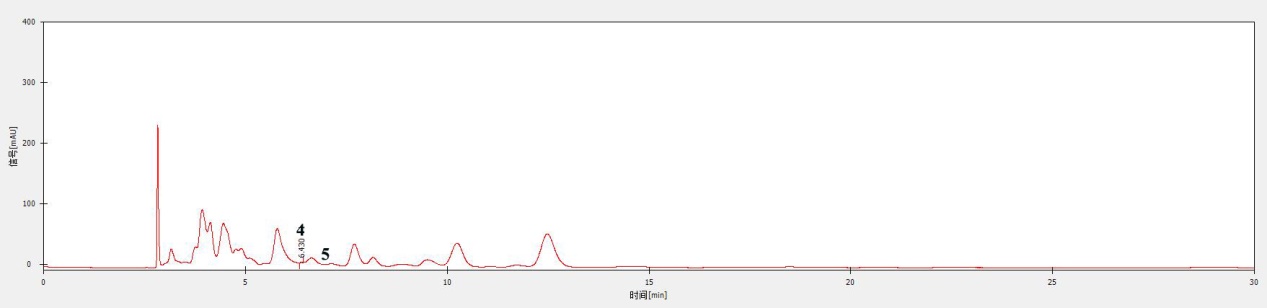


150d-HP


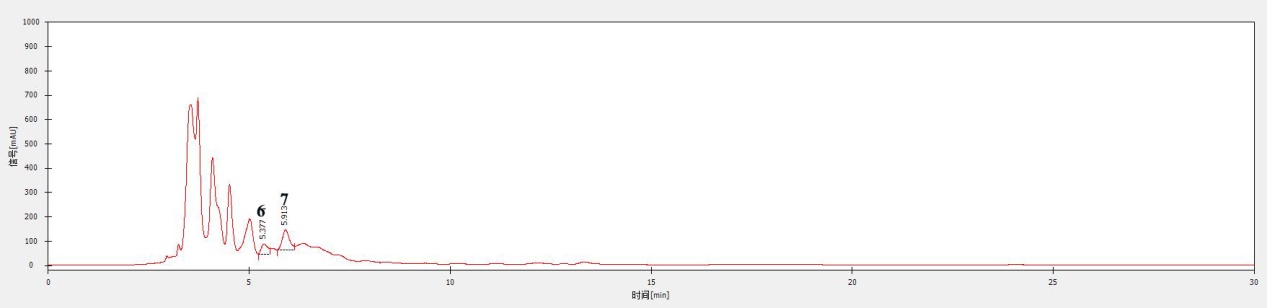


150d-HP


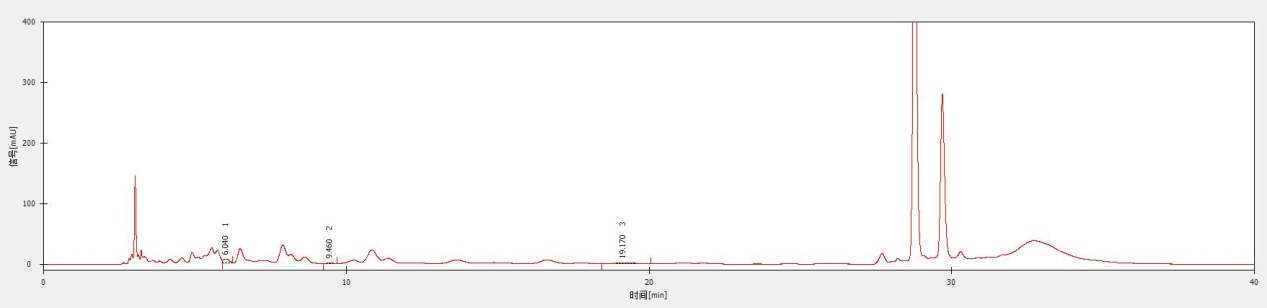


150d-HR


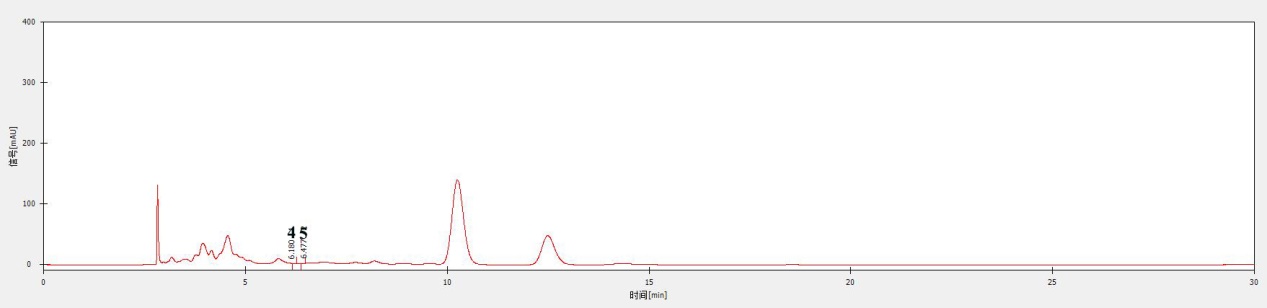


150d-HR


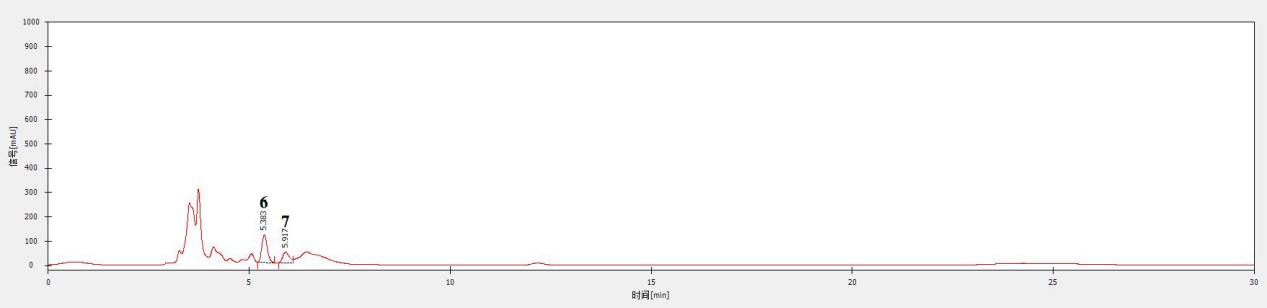


150d-HR


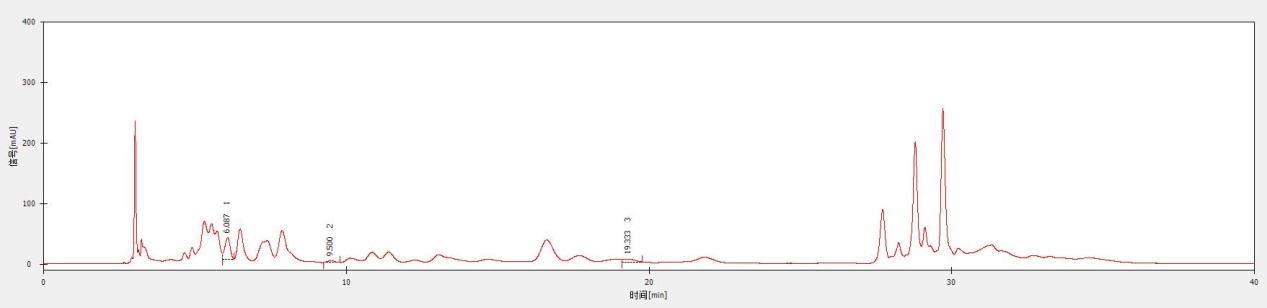


150d-XP


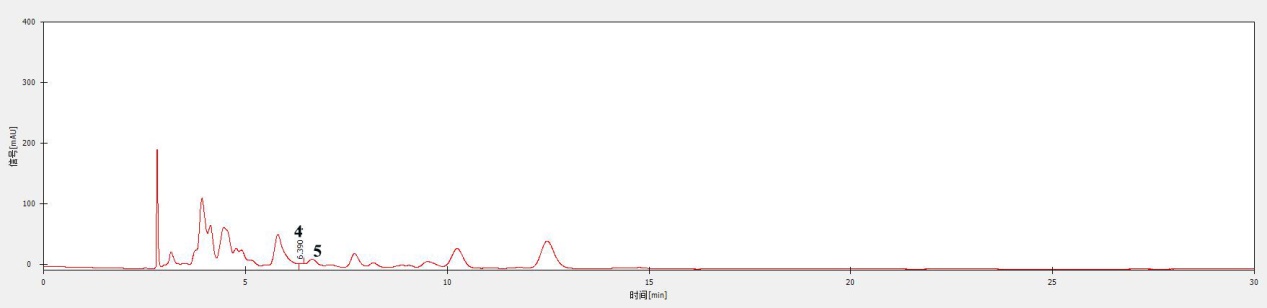


150d-XP


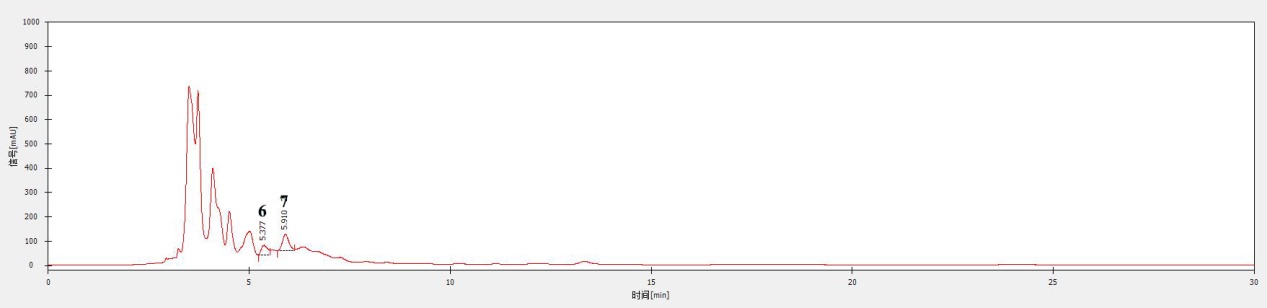


150d-XP


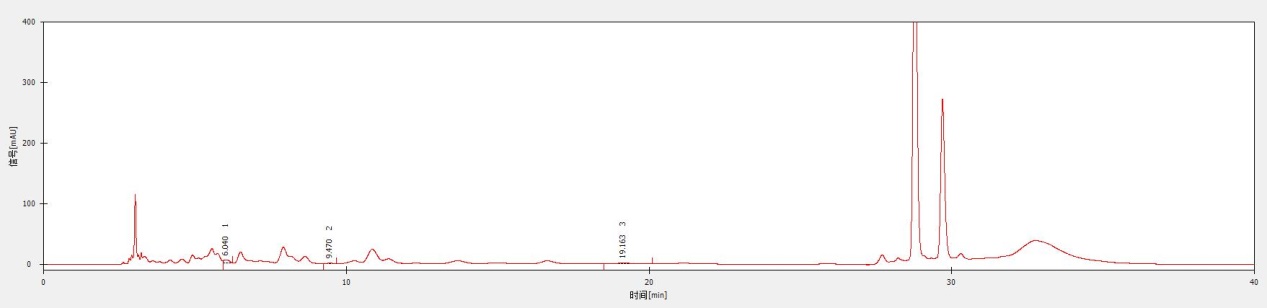


150d-XR


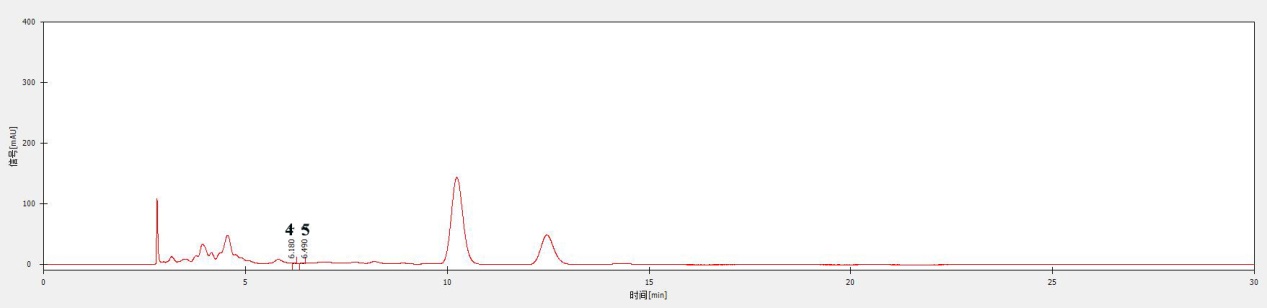


150d-XR


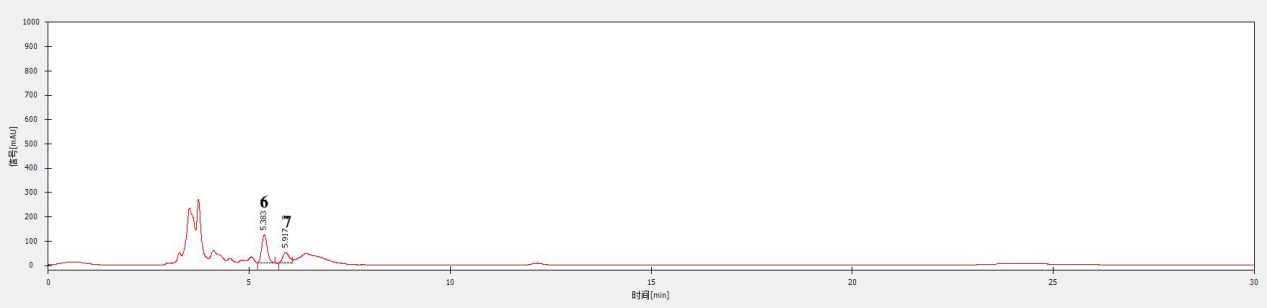


150d-XR


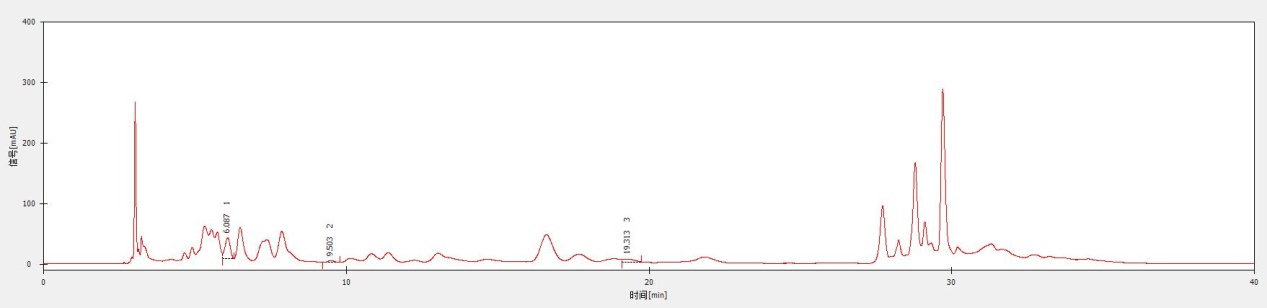


150d-ZP


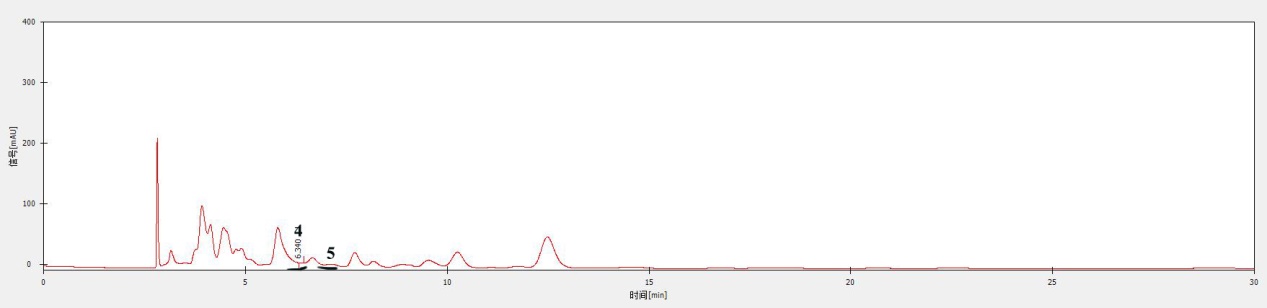


150d-ZP


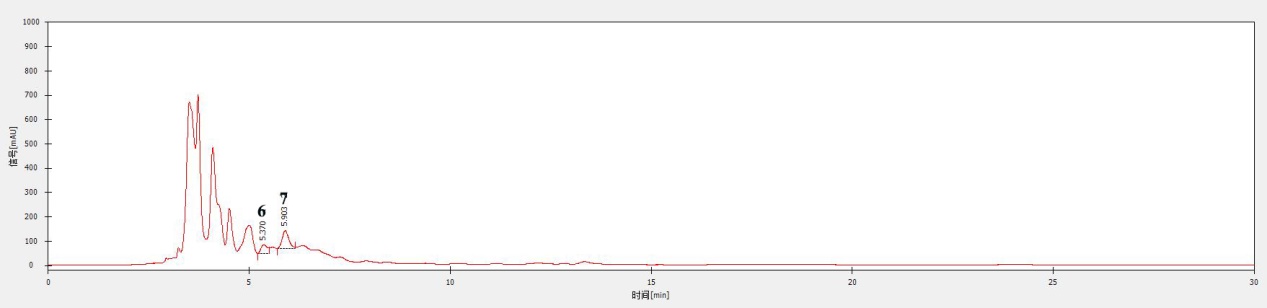


150d-ZP


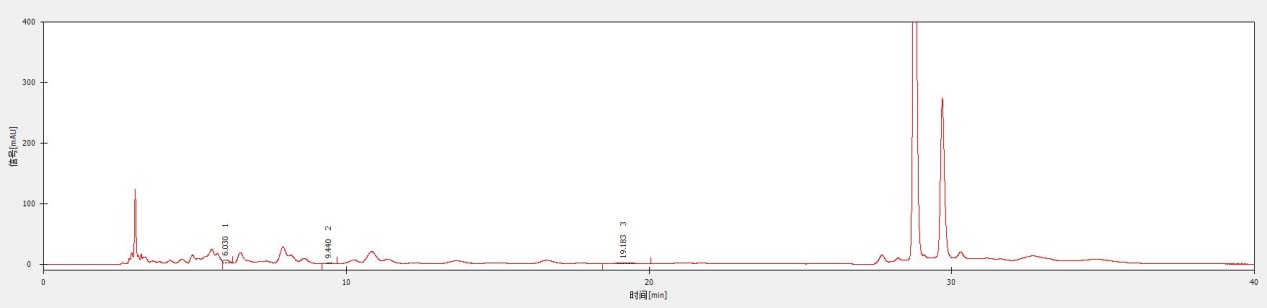


150d-ZR


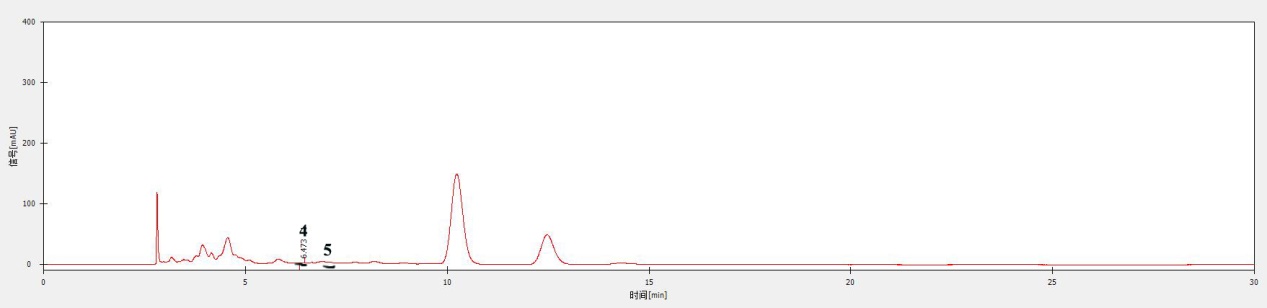


150d-ZR


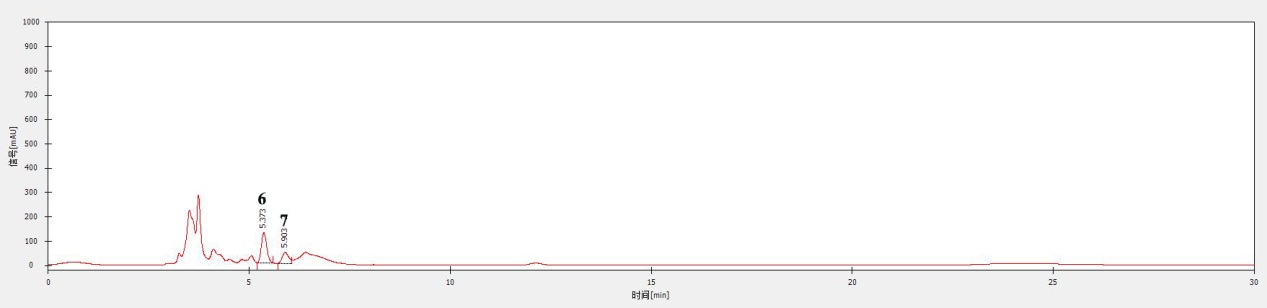


150d-ZR


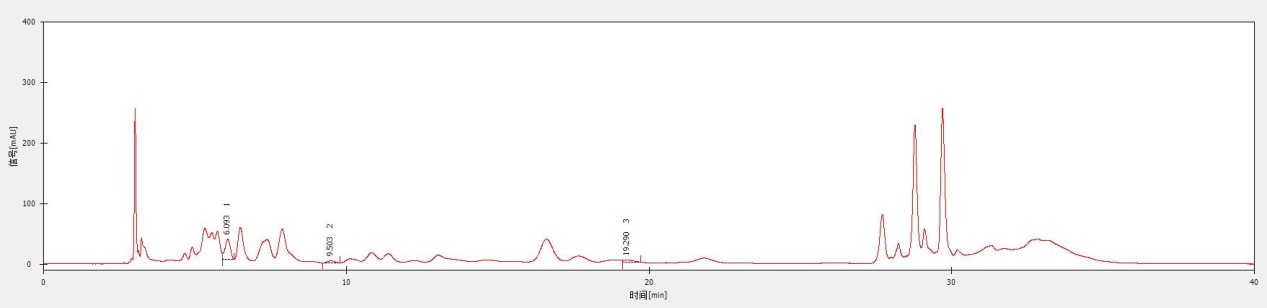


150d-ZCP


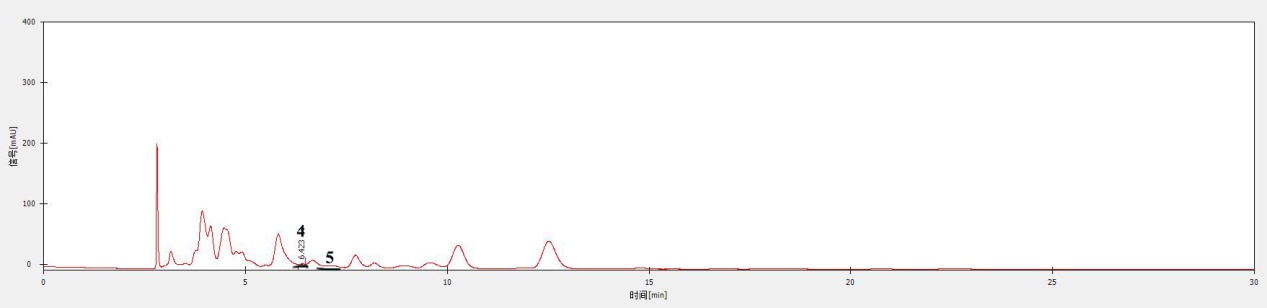


150d-ZCP


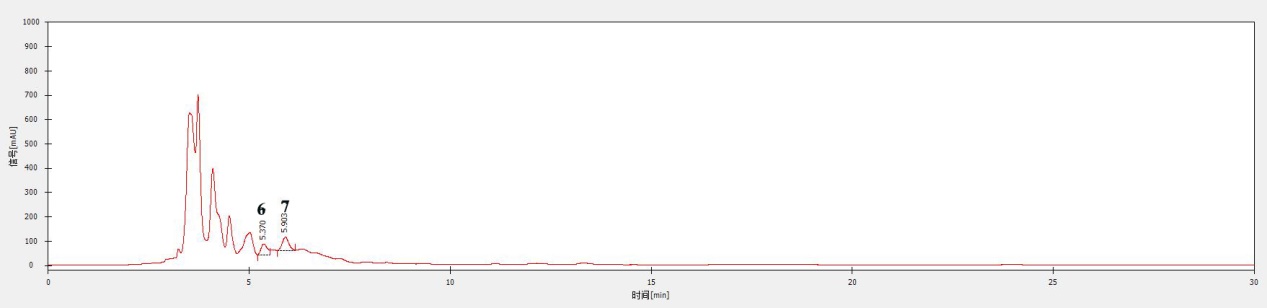


150d-ZCP


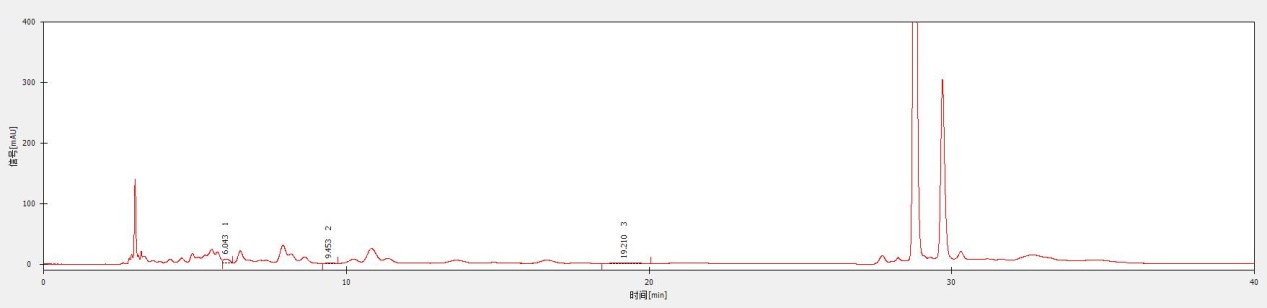


150d-ZCR

150d-ZCR


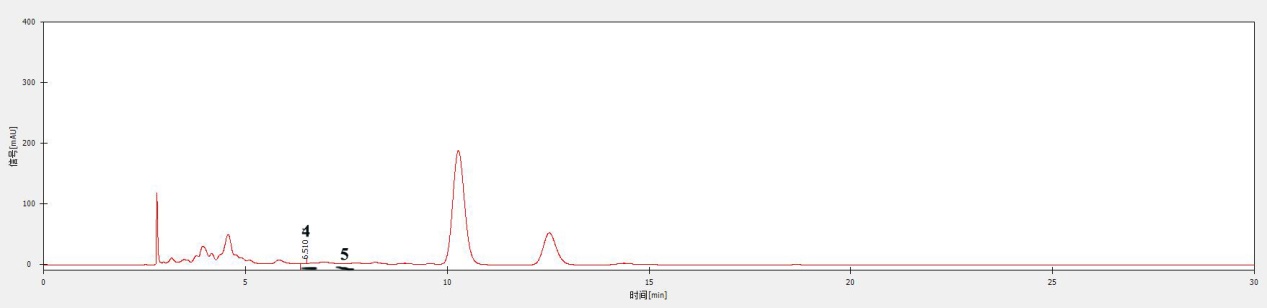


150d-ZCR


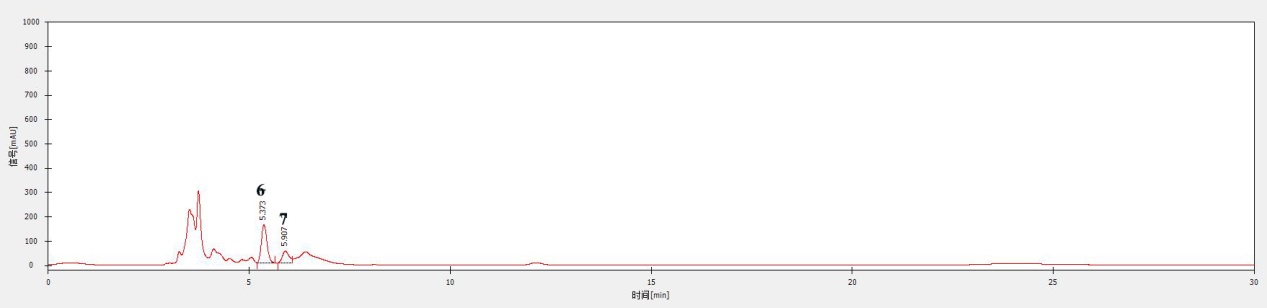


150d-ZCR


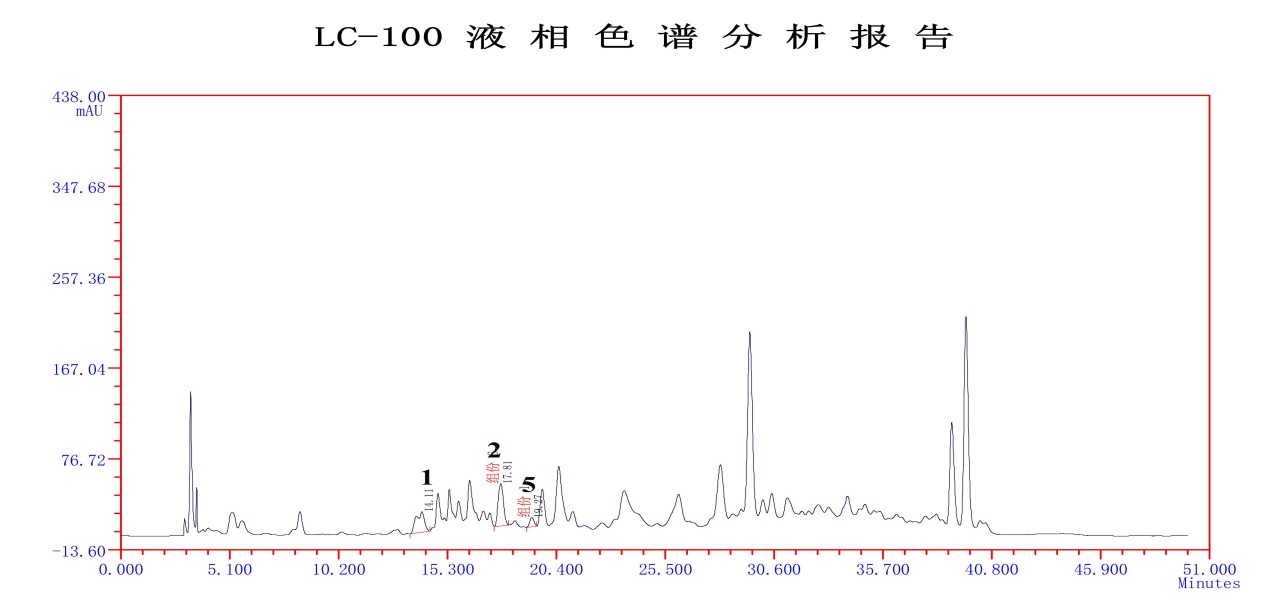


180d-HP


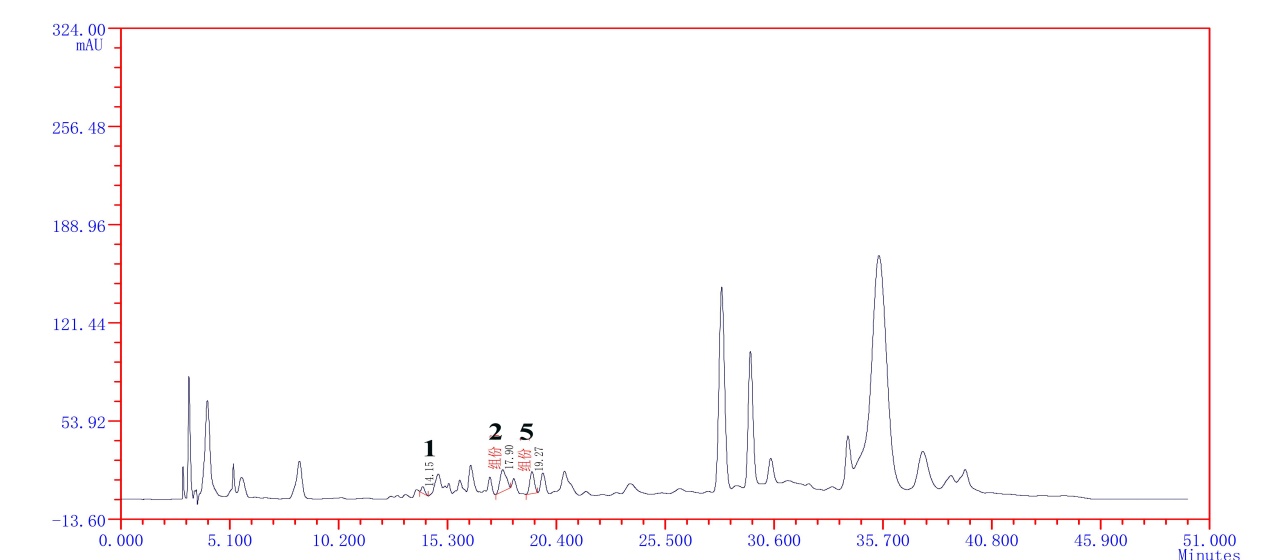


180d-HR


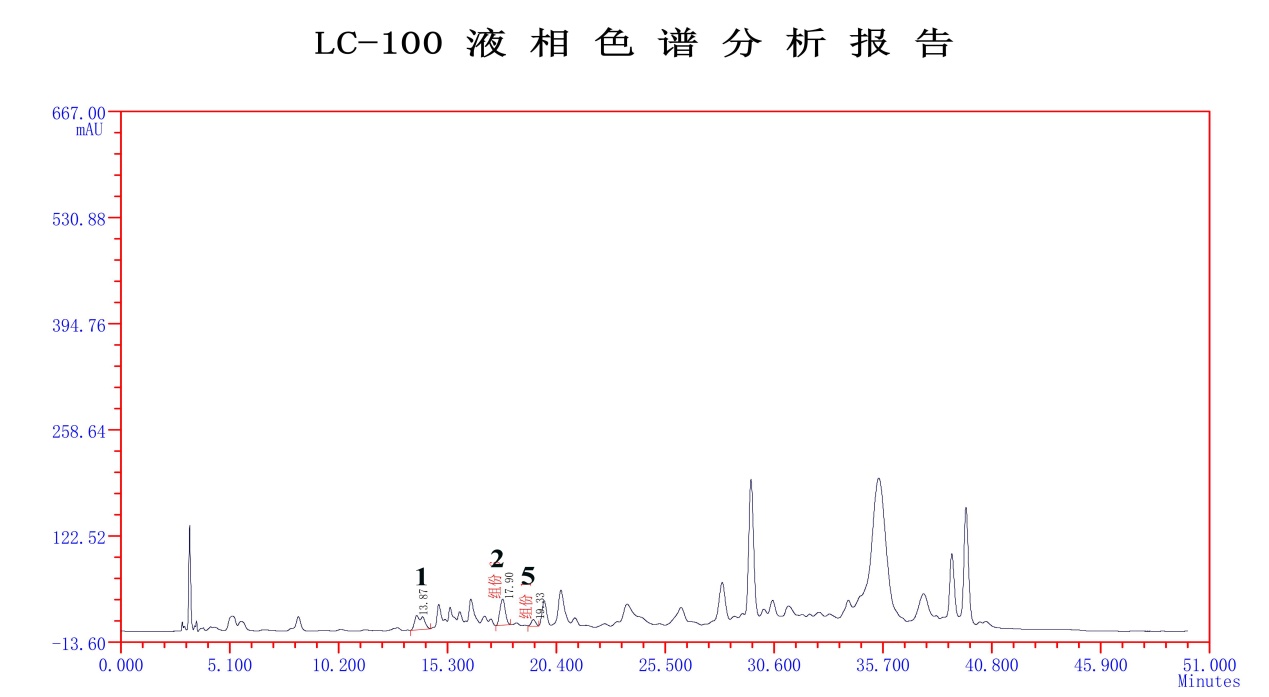


180d-XP


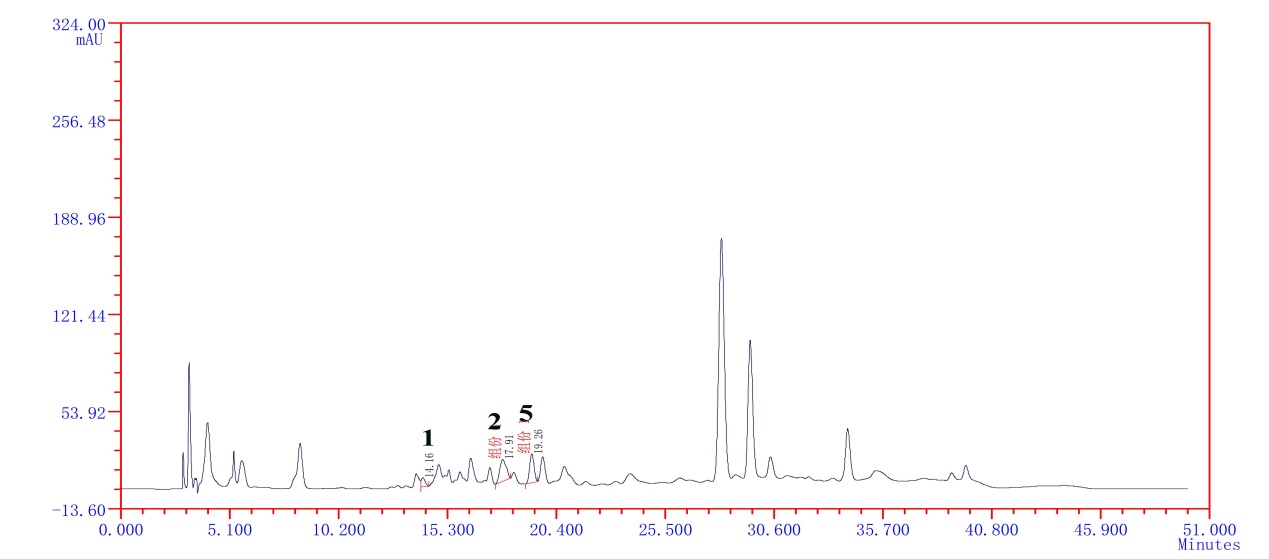


180d-XR


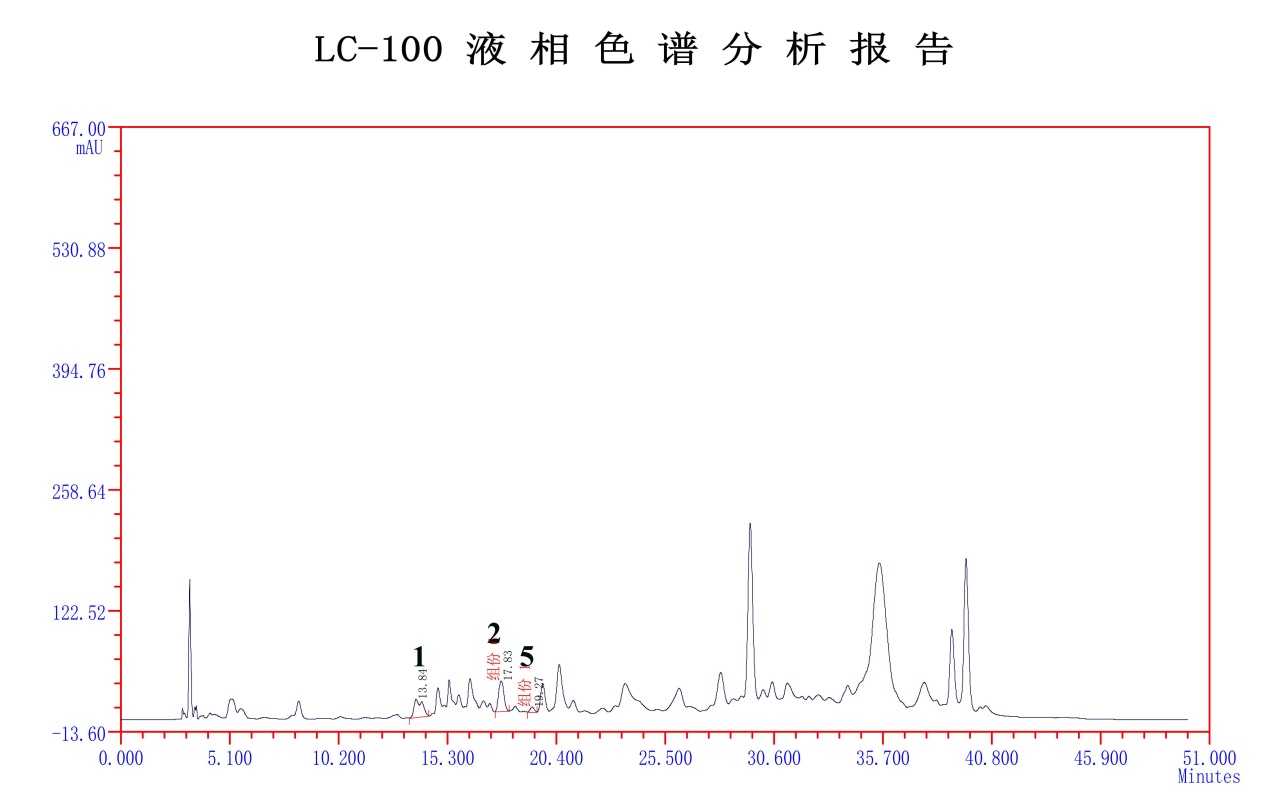


180d-ZP


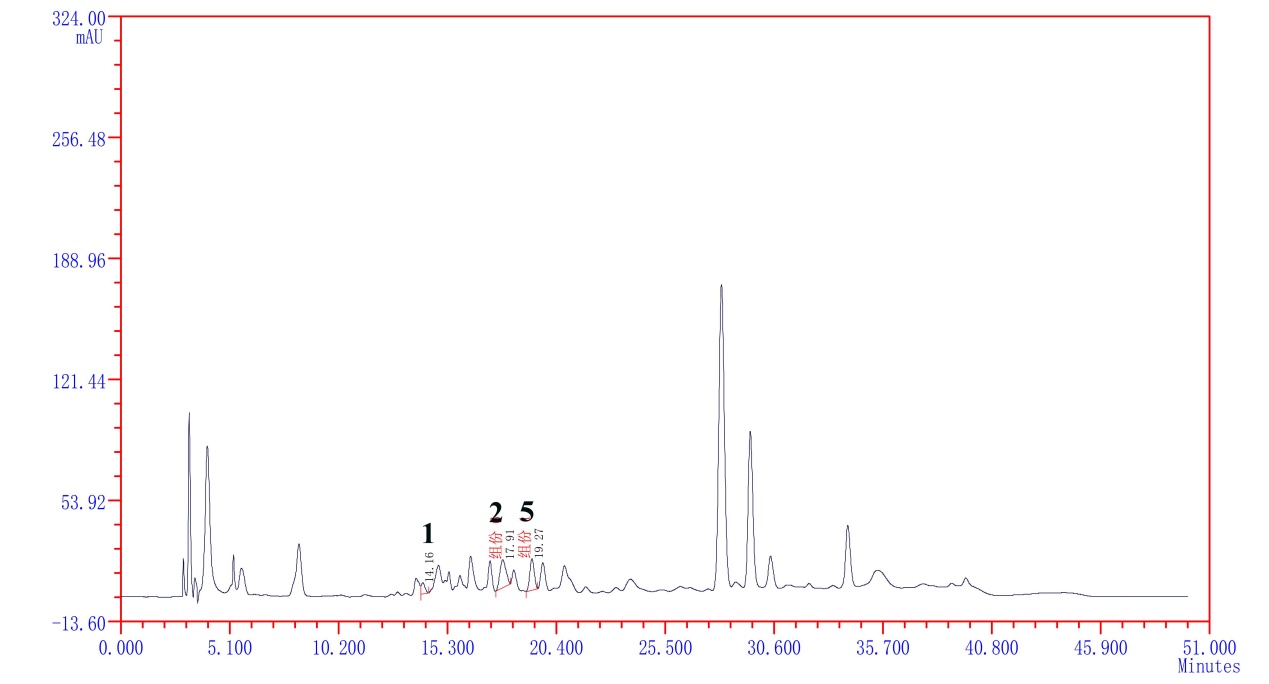


180d-ZR


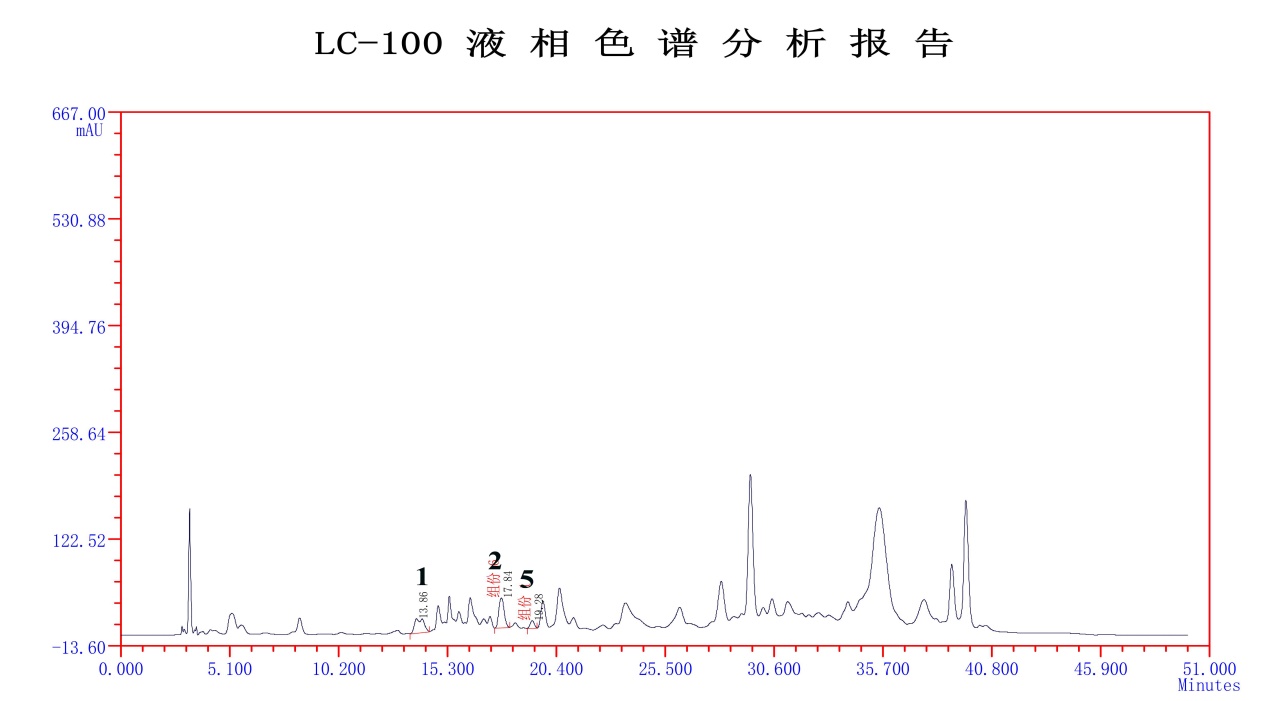


180d-ZCP


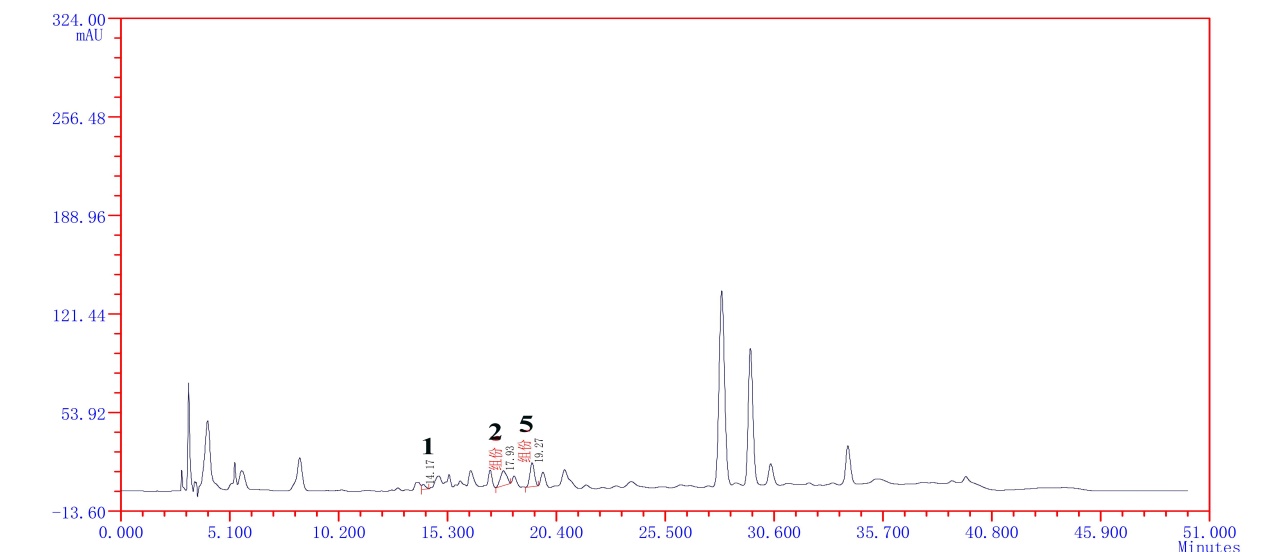


180d-ZCR


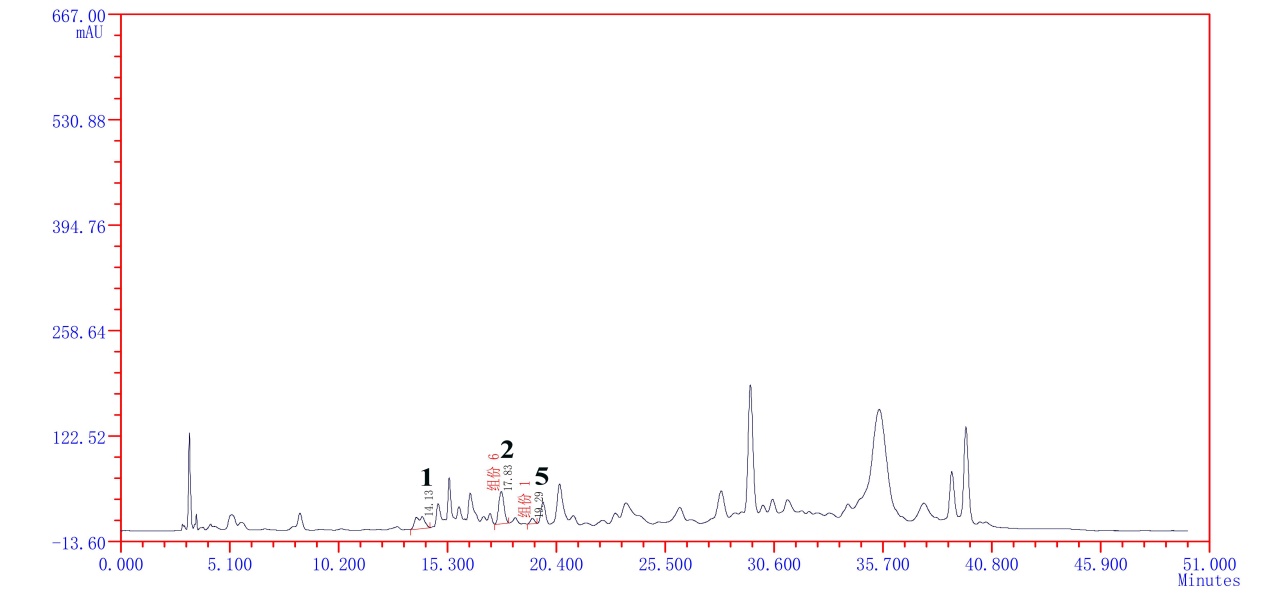


210d-HP


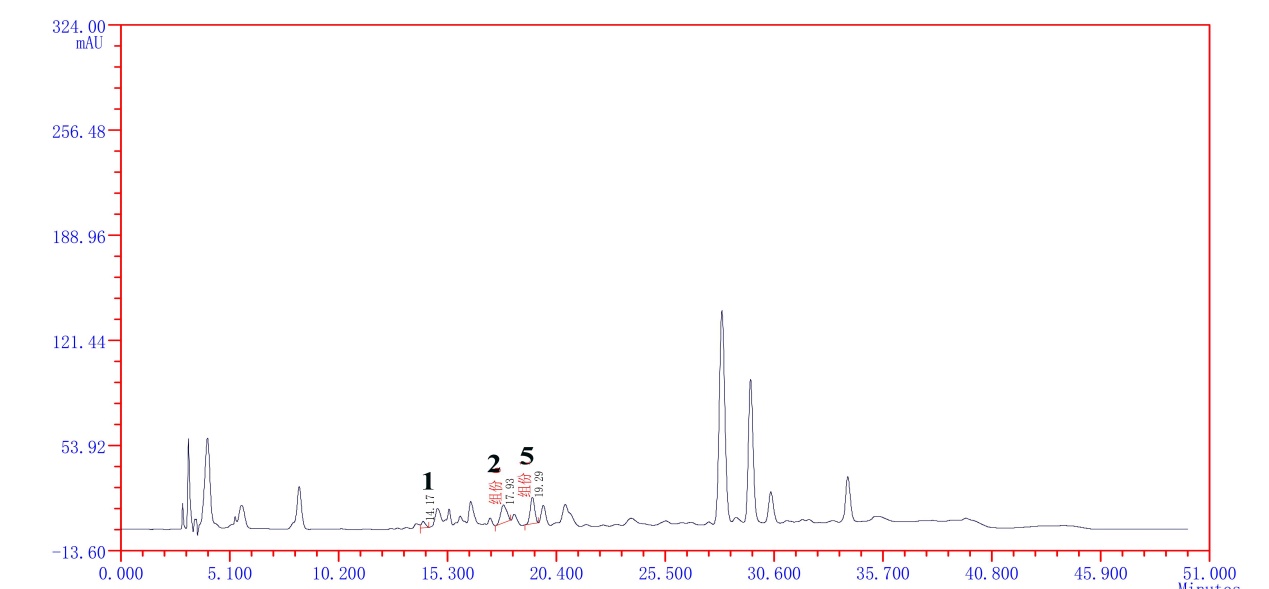


210d-HR


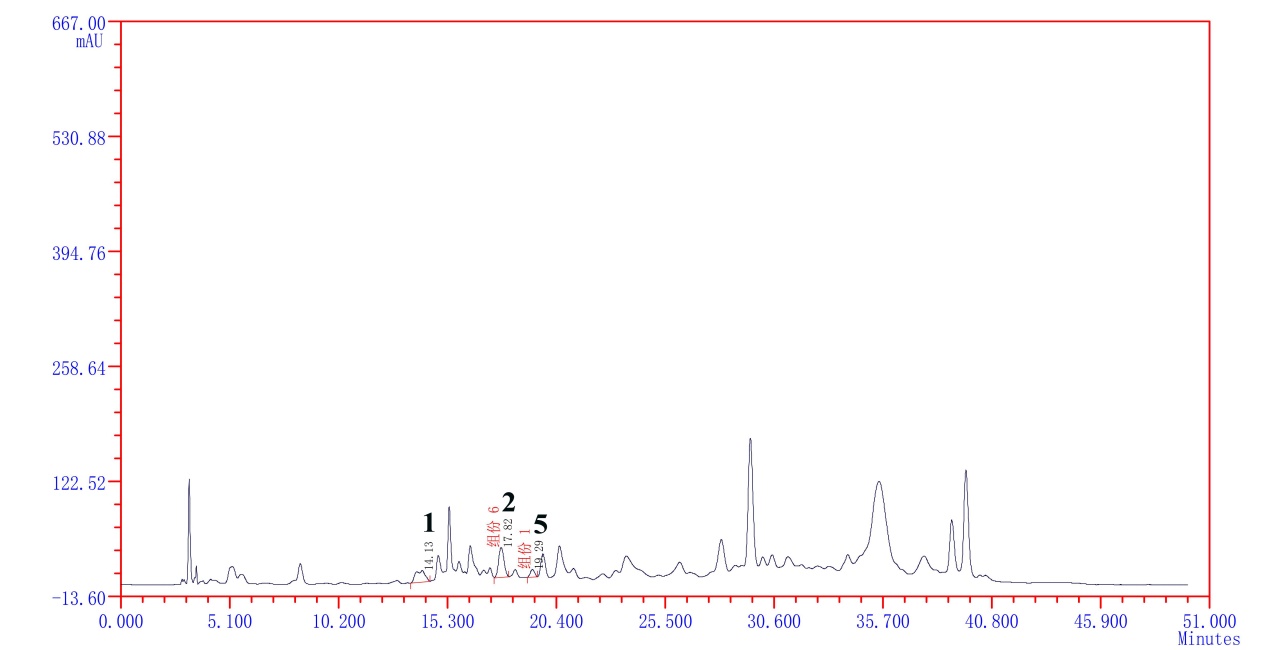


210d-XP


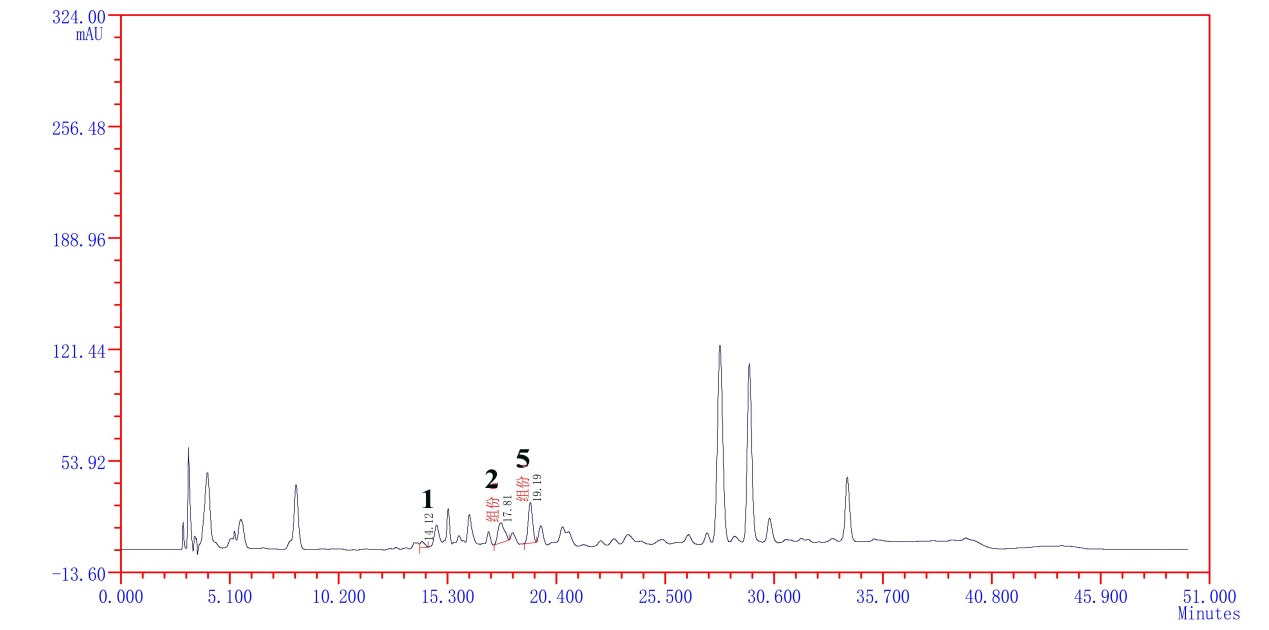


210d-XR


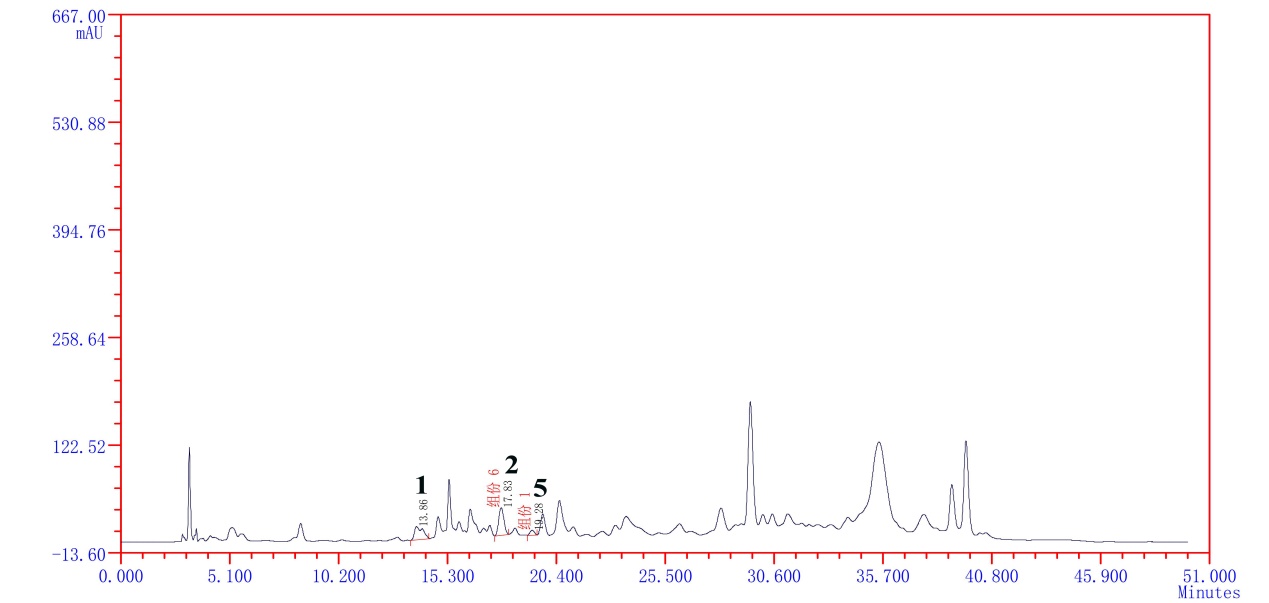


210d-ZP


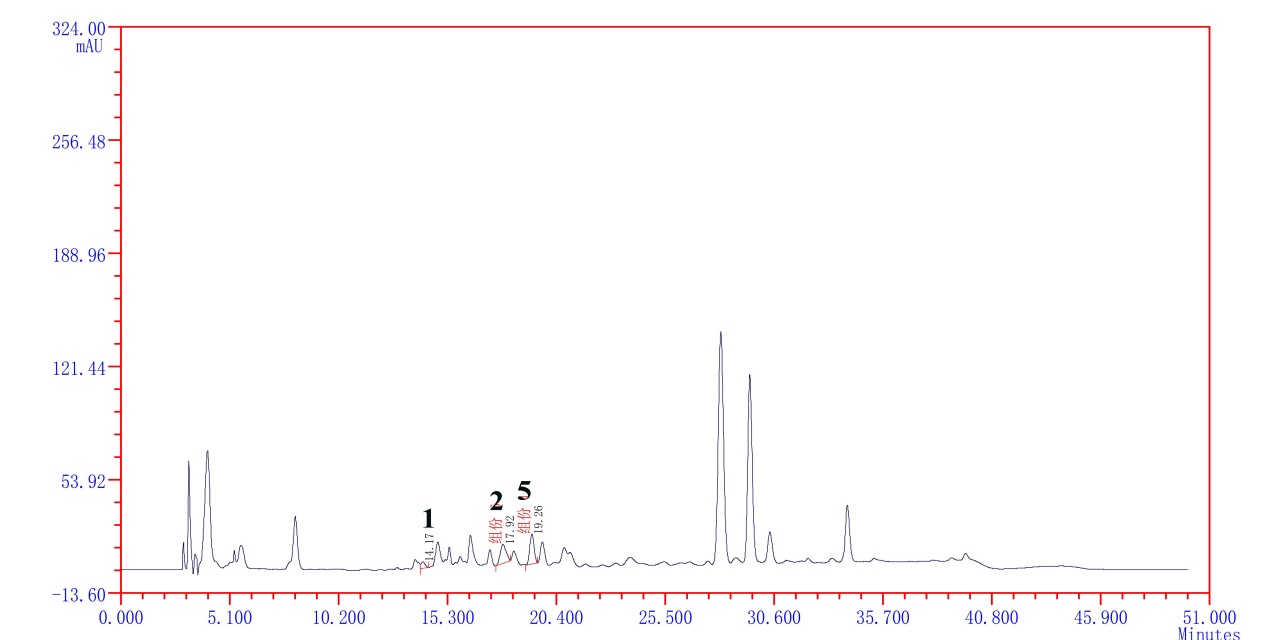


210d-ZR


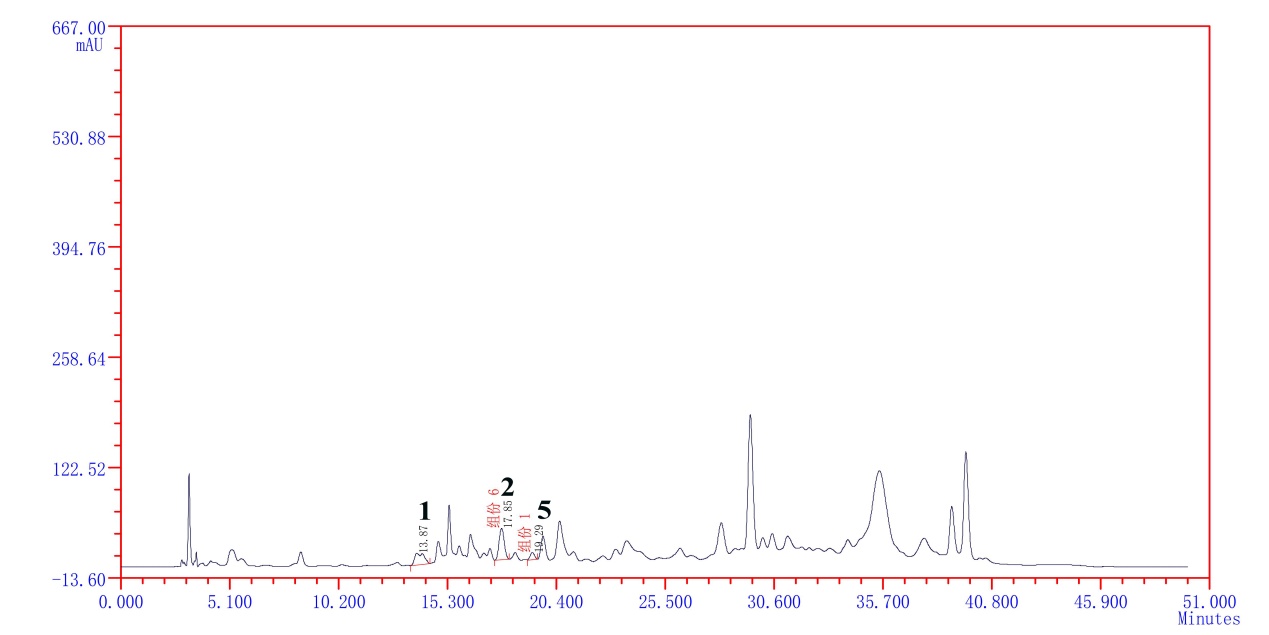


210d-ZCP


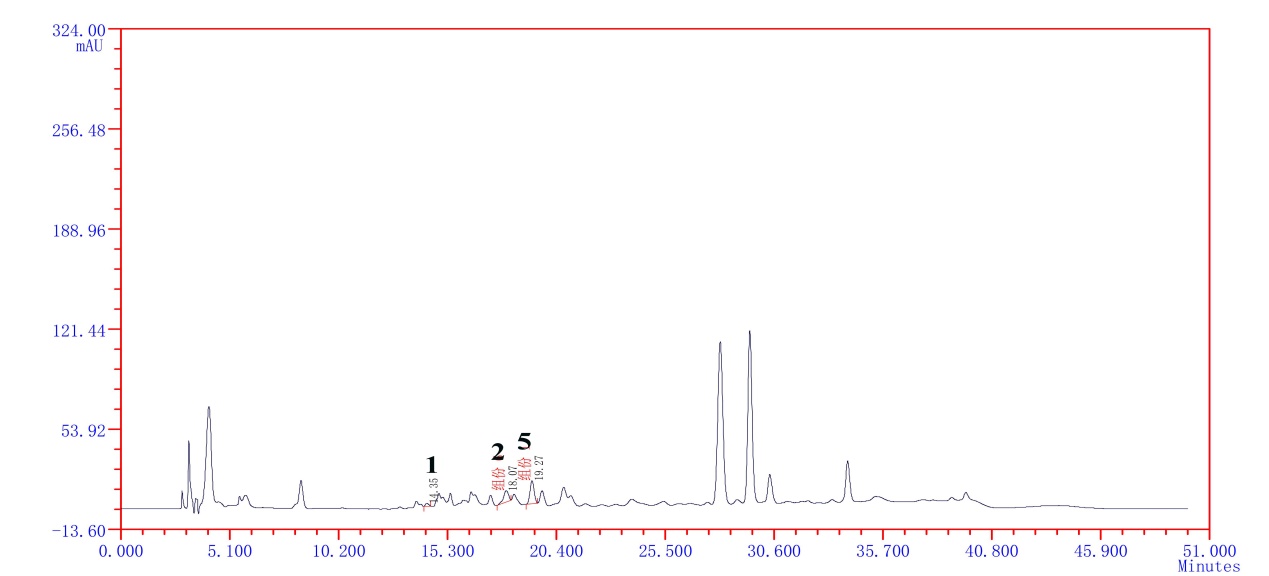


210d-ZCP


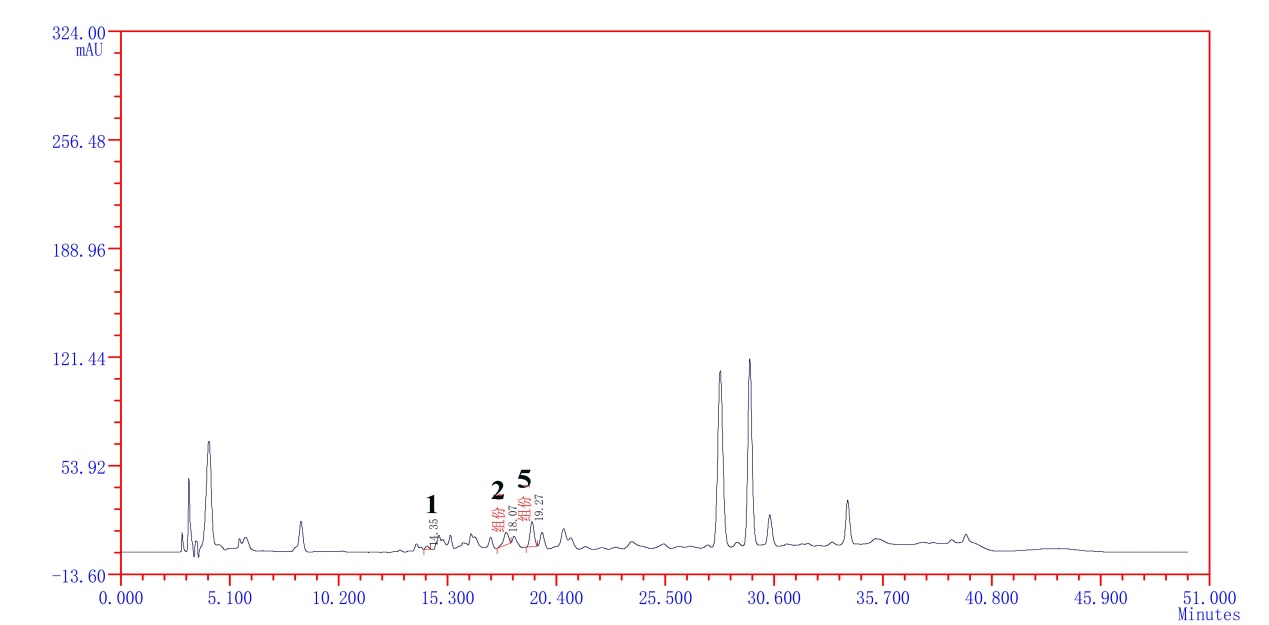


210d-ZCR


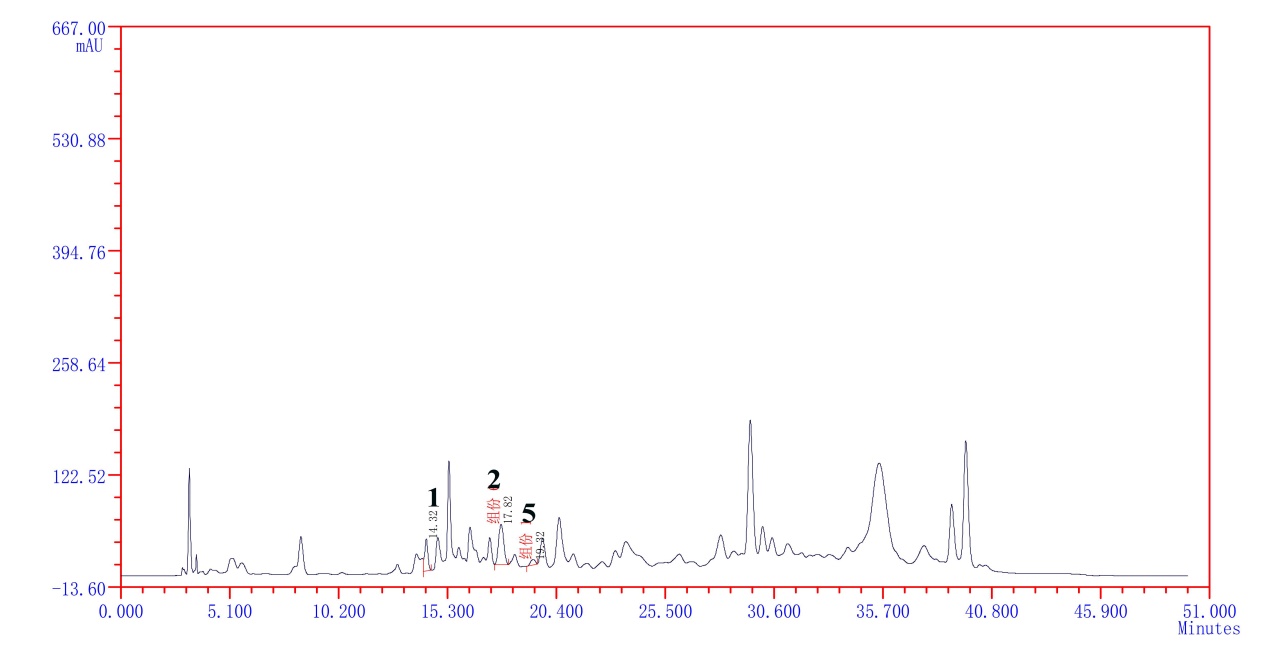


240d-HP


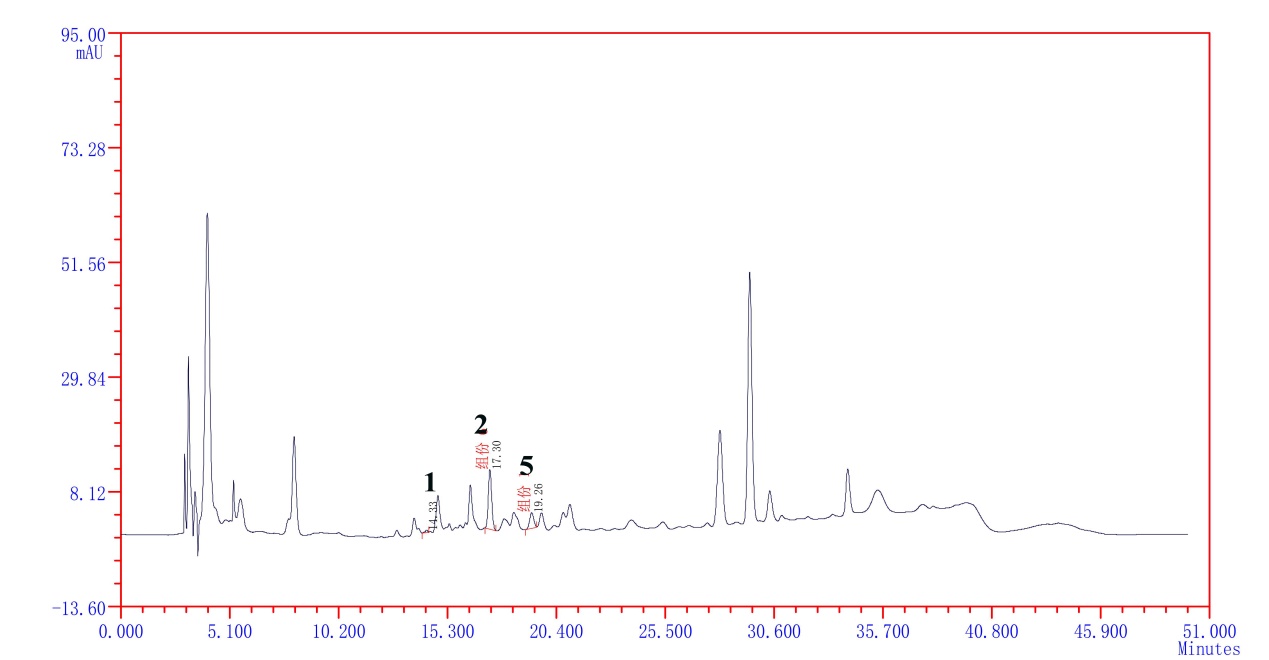


240d-HR


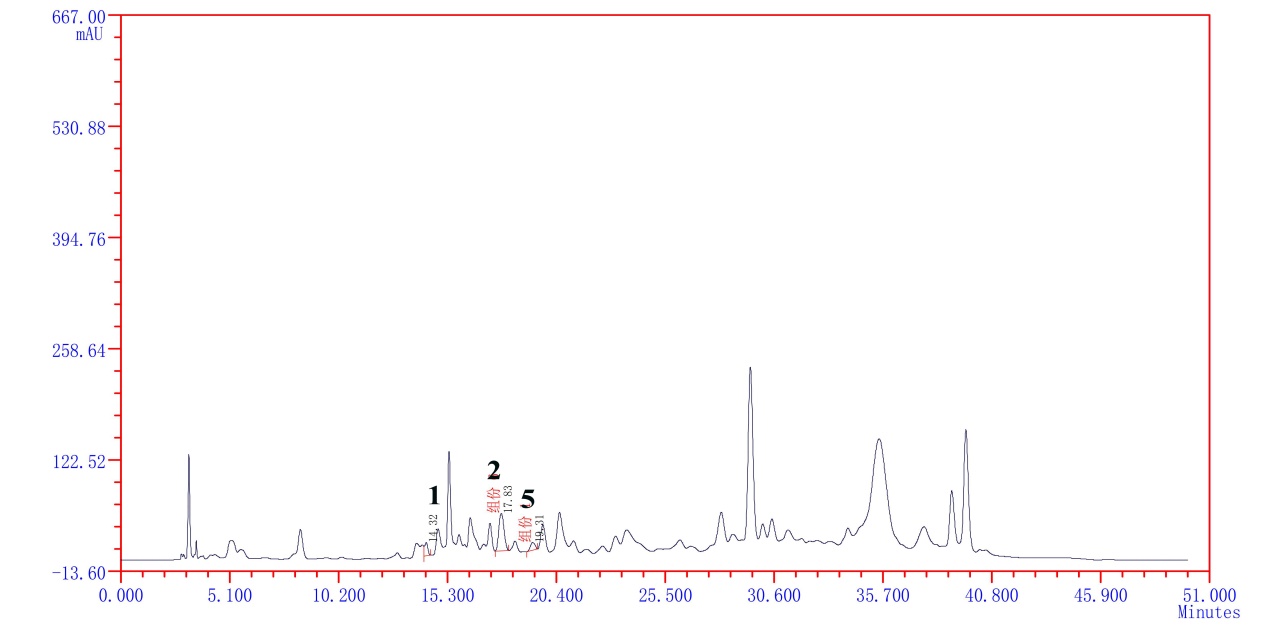


240d-XP


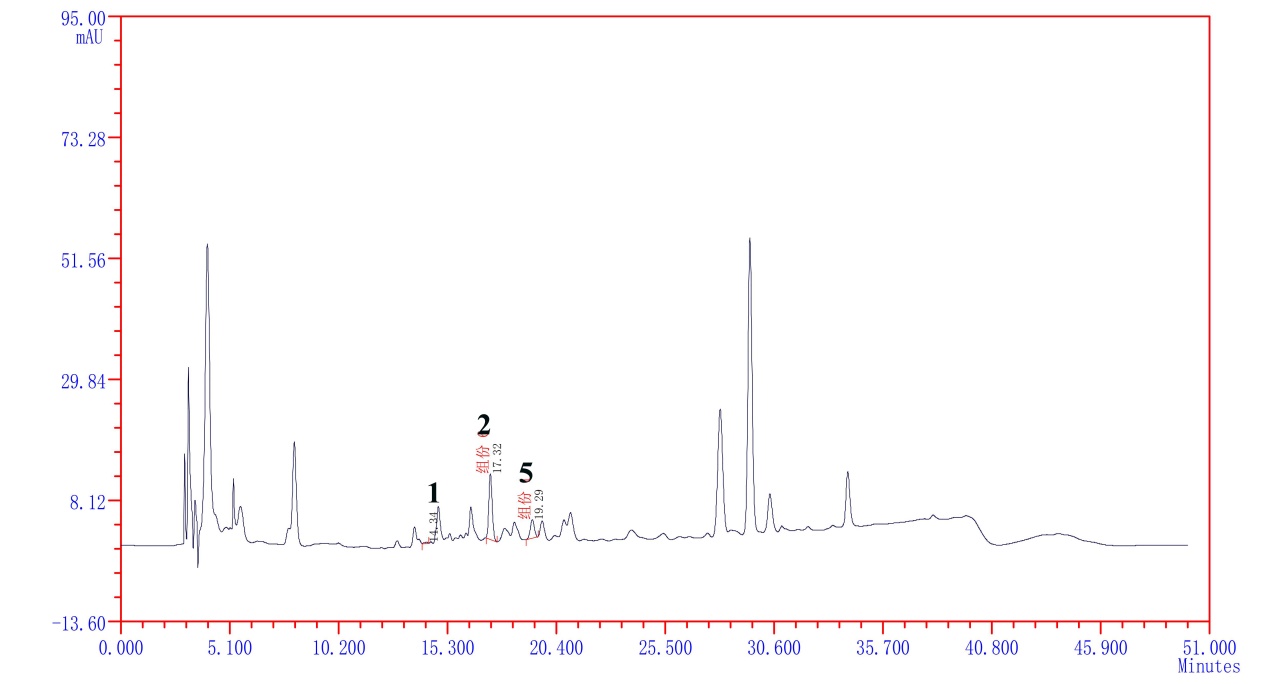


240d-XR


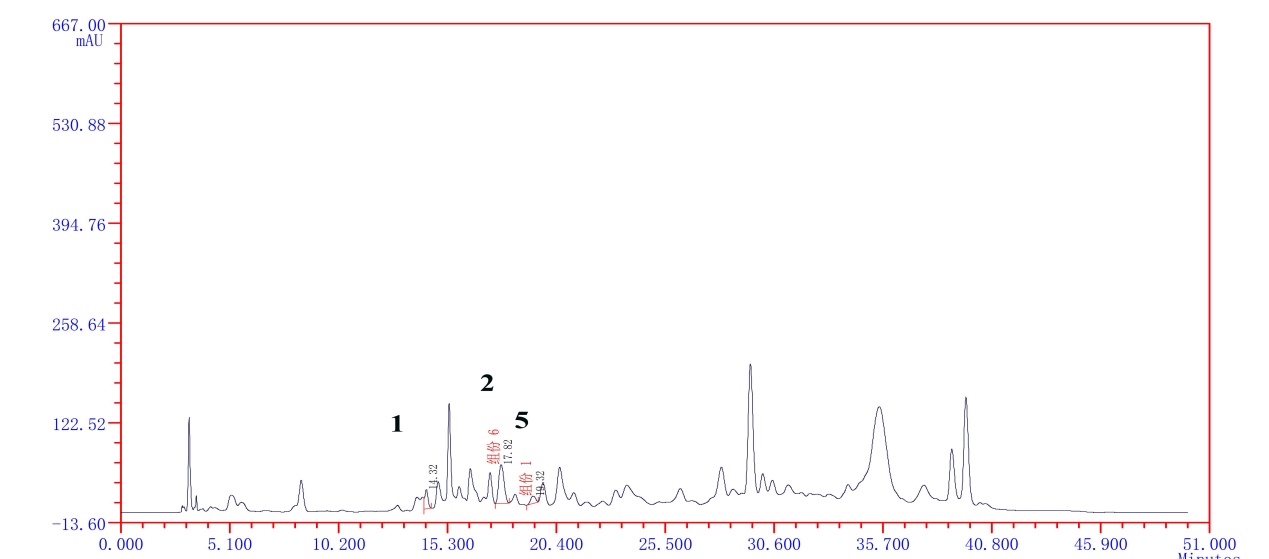


240d-ZP


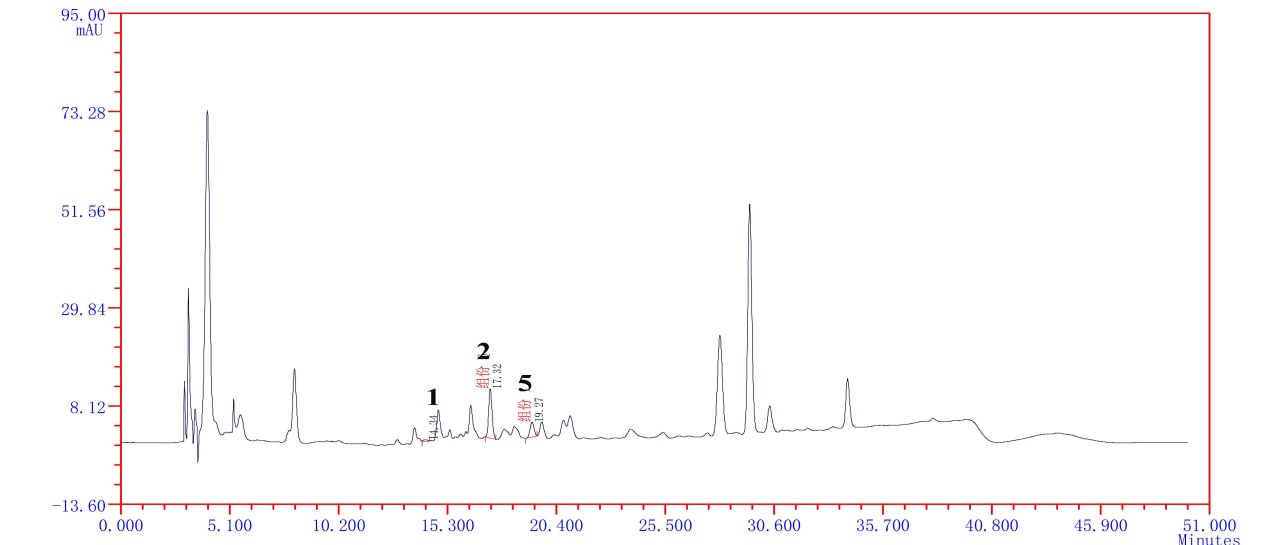


240d-ZR


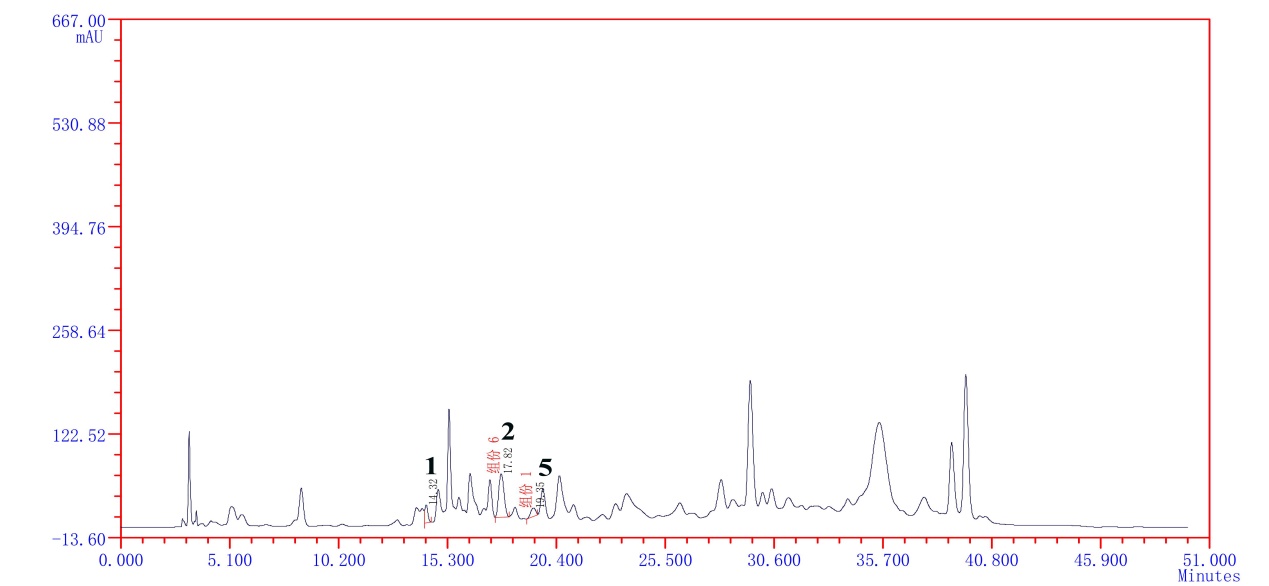


240d-ZCP


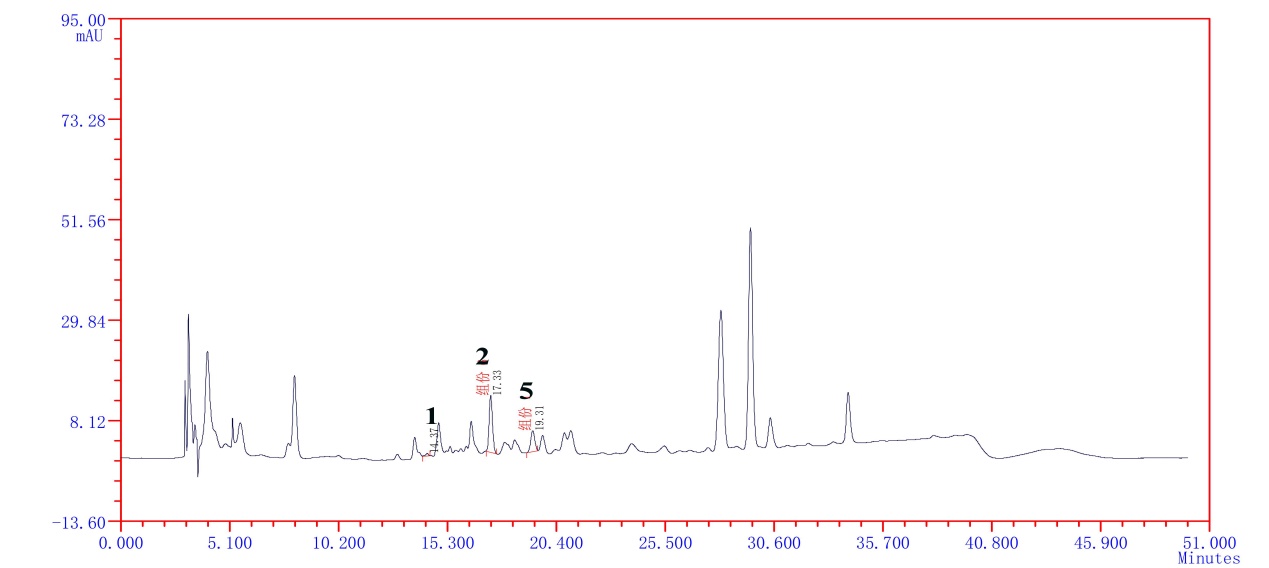


240d-ZCR


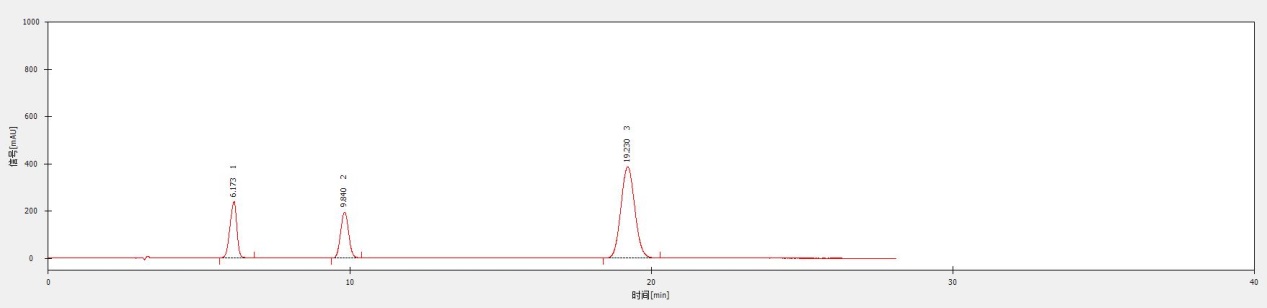


Standard 1


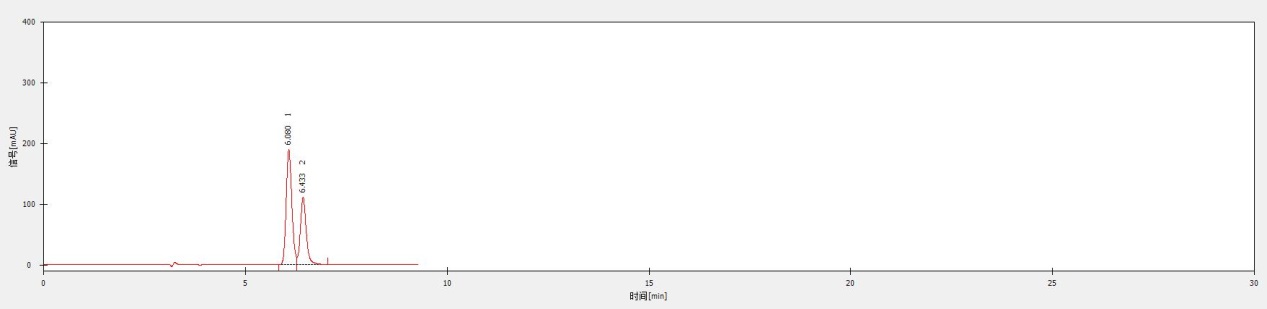


Standard 2


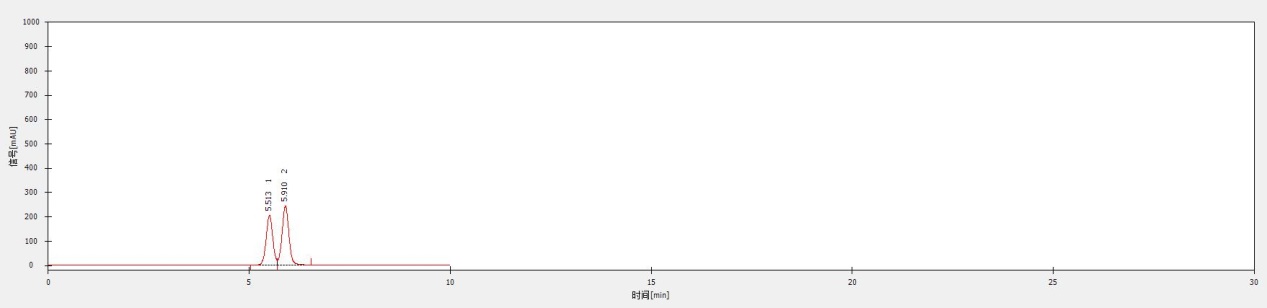


Standard 3

Standard 4

Standard 5

Fig S6 HPLC mass spectrometry of the effects of different rootstocks on the content of seven phenolic acids in 'Orah' fruit

Note: 1. protocatechuic acid; 2. p-hydroxybenzoic acid; 3. p-coumaric acid; 4. caffeic acid; 5. vanillic acid; 6. caprylic acid; 7. ferulic acid; Standard 1: standards for protocatechuic acid, p-hydroxybenzoic acid and p-coumaric acid after 90-150 DAF; Standard 2: standards for vanillic acid and caffeic acid; Standard 3: standards for caprylic acid and ferulic acid after 90-150 DAF; Standard 4: standards for protocatechuic acid, p-hydroxybenzoic acid, vanillic acid after 180-240 DAF; Standard 5: Standars for caffeic acid, caprylic acid, ferulic acid and p-coumaric acid after 180-240 DAF
